# Global burden and trends of high BMI-attributable chronic kidney disease: a comprehensive analysis from 1990 to 2021 and projections to 2035

**DOI:** 10.3389/fnut.2025.1611227

**Published:** 2025-07-22

**Authors:** Huifang Tan, Zhifu Liu, Yongjie Zhang, Kehao Yang, Yiming Zeng, Guoli Li, Zheng Xiao, Yuanwei Li, Yinyin Chen

**Affiliations:** ^1^Department of Nephrology and Laboratory of Kidney Disease, Hunan Provincial People’s Hospital, The First Affiliated Hospital of Hunan Normal University, Changsha, China; ^2^Department of Urology, Hunan Provincial People's Hospital, The First Affiliated Hospital of Hunan Normal University, Changsha, China

**Keywords:** CKD, high BMI, obesity, global burden, demographic and regional disparities, trend projections

## Abstract

**Background:**

High body mass index (BMI) is a major modifiable risk factor for chronic kidney disease (CKD), significantly contributing to its global burden. This study aimed to systematically evaluate the global burden of CKD attributable to high BMI from 1990 to 2021, identify demographic and regional variations, evaluate contributing risk factors, and project future trends through 2035.

**Methods:**

The population-based analysis utilized data from the Global Burden of Diseases, Injuries, and Risk Factors Study (GBD) 2021, encompassing 204 countries and territories across 21 GBD regions. Age-standardized mortality rate (ASMR) and age-standardized disability-adjusted life years rate (ASDR) were assessed using percentage change (PC) and estimated annual percentage change (EAPC). Decomposition analysis quantified drivers of mortality and DALYs changes, while Bayesian age-period-cohort models projected future trends.

**Findings:**

From 1990 and 2021, the ASMR of CKD attributable to high BMI increased from 2.69 (95% UI: 1.37–4.14) to 5.06 (95% UI: 2.7–7.5) per 100,000, and the ASDR rose from 69.13 (95% UI: 35.06–106) to 122.08 (95% UI: 66.25–180.18) per 100,000. Projections estimate continued to increase by 2035, with ASMR reaching 5.81 (95% UI: 3.55–8.07) and ASDR 149.42 (95% UI: 99.45–199.39) per 100,000. Sex and age disparities were evident: males showed higher ASR increases and earlier onset of disease burden, while older females experienced a higher overall burden. Regionally, low- and low-middle SDI areas exhibited the most rapid burden escalation, while some high-income countries achieved burden reduction. Among the attributable etiologies, hypertension and type 2 diabetes mellitus (T2DM) were predominant, with hypertension more common in males (42.05%) and T2DM in females (43.71%).

**Conclusion:**

The global burden of CKD attributable to high BMI has risen markedly over the past three decades, with widening disparities by geography, age, and sex. The COVID-19 pandemic may have further complicated risk dynamics and data quality. These findings highlight an urgent need for globally coordinated, locally adapted prevention strategies, particularly targeting younger males and populations in low-SDI regions.

## Introduction

Obesity, defined by a body mass index (BMI) ≥ 30 kg/m^2^, has emerged as one of the most urgent global public health challenges, currently affecting approximately one in eight individuals worldwide, with prevalence continuing to rise steadily (1, 2). As an independent risk factor for a wide range of non-communicable diseases, high BMI contributes substantially to global morbidity, mortality, and economic burden. According to data from 2019, high BMI was responsible for 5 million deaths from non-communicable diseases, placing immense pressure on healthcare systems and economies (3, 4). Related research projected that the global economic burden of high BMI will exceed $18 trillion between 2030 and 2060, further exacerbating global public health challenges (5–7).

Chronic kidney disease (CKD), which plays a critical role in the progression of end-stage renal disease (8), is a major obesity-related complication driven by mechanisms involving systemic inflammation, insulin resistance, glomerular hyperfiltration, and ectopic lipid deposition in renal tissues. According to the Global Burden of Diseases, Injuries, and Risk Factors Study (GBD) 2021, approximately 700 million individuals were living with CKD globally (9). Between 1990 and 2021, the age-standardized mortality rate (ASMR) of CKD increased by 22%, and projections suggest that by 2040, CKD will rank as the fifth leading cause of years of life lost worldwide (9, 10). While diabetes and hypertension are well-established mediators of obesity-related CKD, high BMI has increasingly been recognized as a direct contributor to kidney dysfunction (11, 12), particularly among younger adults (aged 20–60), who were traditionally considered at lower risk (13).

With the rising global burden of CKD, the lack of robust surveillance systems for CKD and its associated risk factors has led to substantial data gaps, hindering the development of evidence-based prevention strategies and public health policies (14). Furthermore, the prevalence and burden of CKD vary significantly across countries and regions, reflecting disparities in healthcare resources, economic development, and demographic profiles (15). In particular, CKD has emerged as a major public health challenge in low- and middle-income countries, where it impedes economic growth and contributes to the dual burden of malnutrition and health inequities (16). In recognition of this challenge, the Lancet Commission has called for urgent interventions to address the unmet health needs in low- and middle-income countries and to narrow the gap in CKD management between lower- and higher-income settings (17).

Although previous GBD studies have documented the global burden of CKD, few have specifically quantified the impact of high BMI as a modifiable risk factor. This is particularly true for variation across geographic regions, sexes, and sociodemographic development index (SDI) levels. Moreover, existing analyses have largely lacked projections of future disease burden, limiting their utility in long-term health policy planning. Early identification of CKD in high-risk populations, based on demographic characteristics and exposure to key risk factors, is essential for anticipating future trends and informing timely preventive strategies.

To address these gaps, this study used the latest GBD 2021 data (published in 2024) to systematically assess global, regional, and national trends in the burden of CKD attributable to high BMI in 204 countries and territories from 1990 to 2021. It also provided projections for 2035, providing timely and actionable evidence for health system planning. By highlighting geographic, sex, and socioeconomic disparities in the burden of CKD attributable to high BMI, this study aims to inform targeted prevention efforts, optimize resource allocation, and support global public health efforts, particularly in low- and middle-income countries, where CKD remains under-recognized and under-treated.

## Methods

### Data source and disease definition

The CKD data analyzed in this study are derived from the GBD 2021, which highlights health disparities across various age, sex, geographic, and socioeconomic groups. Our research specifically investigates the burden of CKD associated with high BMI across 21 GBD regions and 204 countries and territories from 1990 to 2021. This comprehensive dataset is freely accessible through the Global Health Data Exchange^1^ (18). In GBD 2021, CKD is classified as a Level 3 cause, comprising five Level 4 subcategories, including type 1 and type 2 diabetes mellitus, hypertension, glomerulonephritis, and other or unspecified causes. The primary outcome measures in our study were mortality and DALYs related to CKD attributable to high BMI. DALYs are defined as the total number of healthy life years lost from disease onset to death, serving as a standardized metric for assessing disease burden.

In this analysis, CKD was defined as an estimated glomerular filtration rate (eGFR) less than 60 mL/min/1.73 m^2^ or a urinary albumin-to-creatinine ratio (uACR) greater than 30 mg/g, consistent with criteria commonly used in epidemiological studies (18). While CKD is clinically classified by staging based on both eGFR and uACR, this simplified definition reflects an increased risk of disease progression and aligns with the GBD analytical framework (18). This definition is consistent with the International Statistical Classification of Diseases and Related Health Problems, 11th Revision (ICD-11), and aligns with corresponding code GB61. Additionally, high BMI in adults (ages 20 and older) is defined as a BMI greater than 23 kg/m^2^, along with the Methods Web Portal for risk factors specific definitions of GBD (2). Some results are presented stratified by the Socio-Demographic Index (SDI), a composite measure that includes lag-distributed income per capita, average years of education, and fertility rates among females under 25 years of age (19). Countries were classified into five categories based on the SDI: low SDI (<0.47), low-middle SDI (0.47–0.62), middle SDI (0.62–0.71), high-middle SDI (0.71–0.81), and high SDI (>0.81).

### Statistical analysis

Age standardization was applied to adjust mortality and DALY rates, facilitating meaningful comparisons across diverse global populations. ASR was calculated based on the GBD standard population. A descriptive analysis was conducted to comprehensively assess the burden of CKD attributable to high BMI. Global case numbers and age-standardized rates (ASRs) of mortality and DALYs for CKD attributable to high BMI in 1990 and 2021 were compared and visualized at multiple levels: globally, across 21 GBD regions, 204 countries and territories, and five SDI quintiles (high, high-middle, middle, low-middle, and low). Trends in global case numbers and ASRs of mortality and DALYs by sex (male and female, physiological distinction) were analyzed from 1990 to 2021, with detailed breakdowns by year and age distribution. The influence of high BMI on CKD burden from various etiologies was evaluated by analyzing changes in mortality and DALY numbers and their proportional contributions by cause over time, stratified by sex. Furthermore, we analyzed the relationship between the SDI and the CKD burden attributable to high BMI across different locations and periods.

In this study, the percentage change (PC) was used to quantify differences in case numbers, mortality rates, and DALYs between 2021 and 1990, calculated as: *PC = (Cases or rate 2021 – Cases or rate 1990)/Cases or rate 1990*. To assess the overall trends in CKD burden attributable to high BMI from 1990 to 2021, we used the EAPC. A linear regression model was used to examine the relationship between time and the natural logarithm (ln) of the ASR: *y = α + βx + 𝜖*, where y denotes ln (ASR), *x* represents the calendar year, and 𝜖 is the error term. The EAPC was calculated using the formula *EAPC = 100 × [exp(β) – 1]*, with its 95% confidence interval (CI) derived from the regression model.

To better understand the factors driving changes in mortality and DALYs for CKD attributable to high BMI from 1990 to 2021, we performed decomposition analyses stratified by sex. These analyses evaluated the contributions of aging, population growth, and epidemiological changes to the observed shifts in CKD burden attributable to high BMI over the past 32 years (20, 21). Mortality and DALY numbers for each location were calculated using the following formula: $\text{Numbe}r_{\mathrm{ay},\mathrm{py},\mathrm{ey}}=\sum_{i=1}^{n}(a_{i,y}\cdot p_{y}\cdot e_{i,y})$ , where $\text{Numbe}r_{\mathrm{ay},\mathrm{py},\mathrm{ey}}\;$ represents the mortality or DALY count attributable to age structure, population size, and corresponding rates in year *y*; $a_{i,y}\;$ is the proportion of the population in age category *i* (out of *n* age categories) in year *y*; $p_{y}\;$ is the total population in year *y*; and $e_{i,y}\;$ is the specific rate for age category *i* in year *y*. The contribution of each factor to changes in mortality and DALYs from 1990 to 2021 was assessed by isolating the effect of each factor while keeping the others constant.

A projection of future CKD burden attributable to high BMI was performed for the coming decades. The Bayesian age-period-cohort (BAPC) model, incorporating integrated nested Laplace approximation (INLA) (22, 23), was applied for this analysis due to its superior coverage and precision over the traditional APC model. This approach generated global predictions of disease burden up to 2035. Parameter uncertainty was addressed at each modeling step by randomly sampling 1,000 values from the distribution of each parameter and propagating this uncertainty through subsequent steps of the analysis. To quantify uncertainty in the final estimates, 95% uncertainty intervals (UIs) were derived by generating 1,000 random samples from the distribution of the estimates and identifying the 2.5th and 97.5th percentiles. Data cleaning, computation, and graph plotting in this study were conducted using R software (version 4.4.2), available at https://cran.r-project.org/.

### Ethics statement

The institutional review board exempted this study from ethical approval since it utilized anonymized, publicly available epidemiological data. As a result, patient-informed consent forms were not required for downloading the data from the database.

## Results

### Global burden of CKD attributable to high BMI in 2021

**Figure 1:**
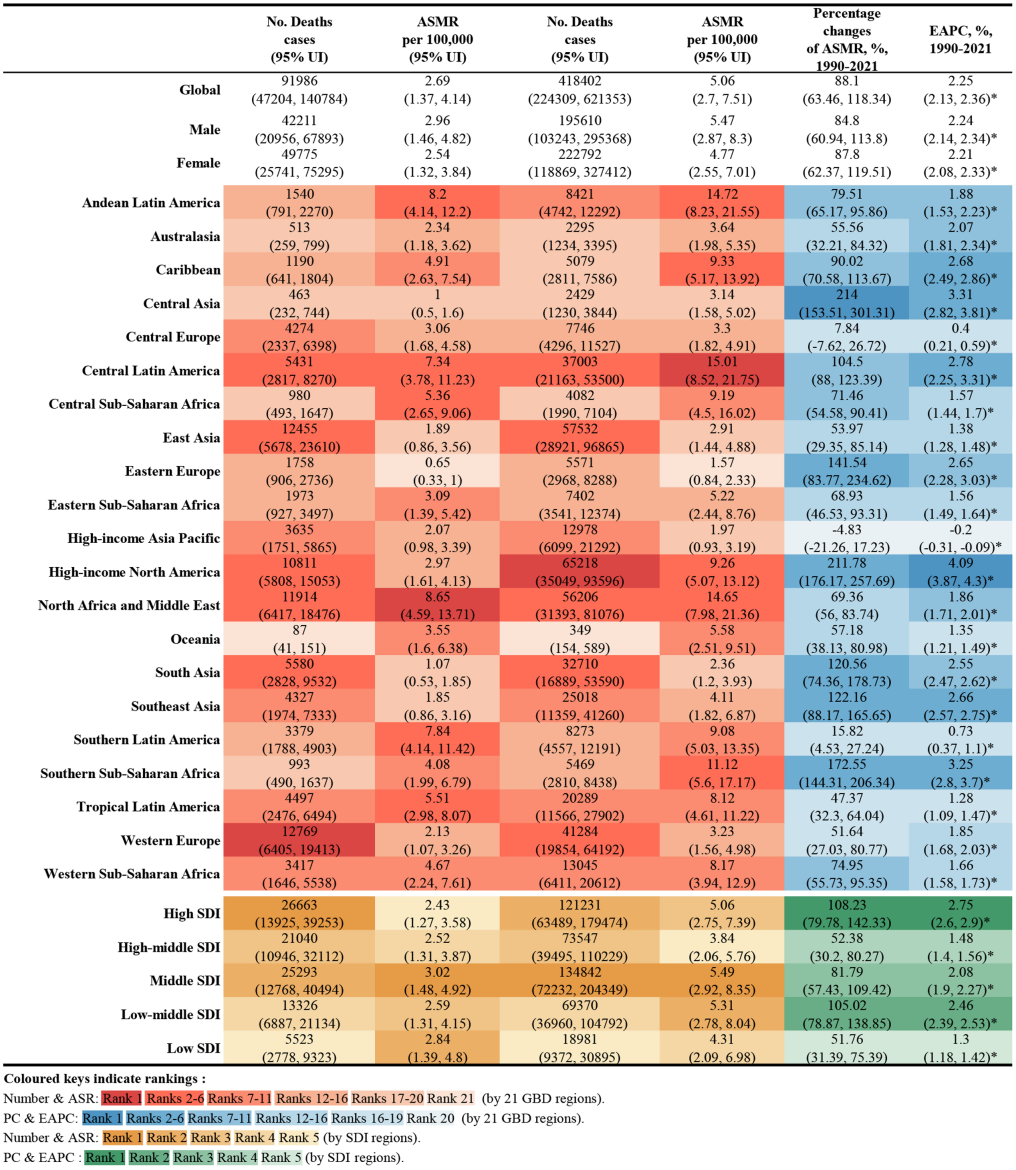
Number of deaths and ASMR of high BMI-attributable CKD in 1990 and 2021, and the percentage changes and EAPC of ASMR from 1990 to 2021, by sex and location. BMI, body mass index; CKD, chronic kidney disease; ASMR, age-standardized mortality rate; EAPC, estimated annual percentage change.

**Figure 2:**
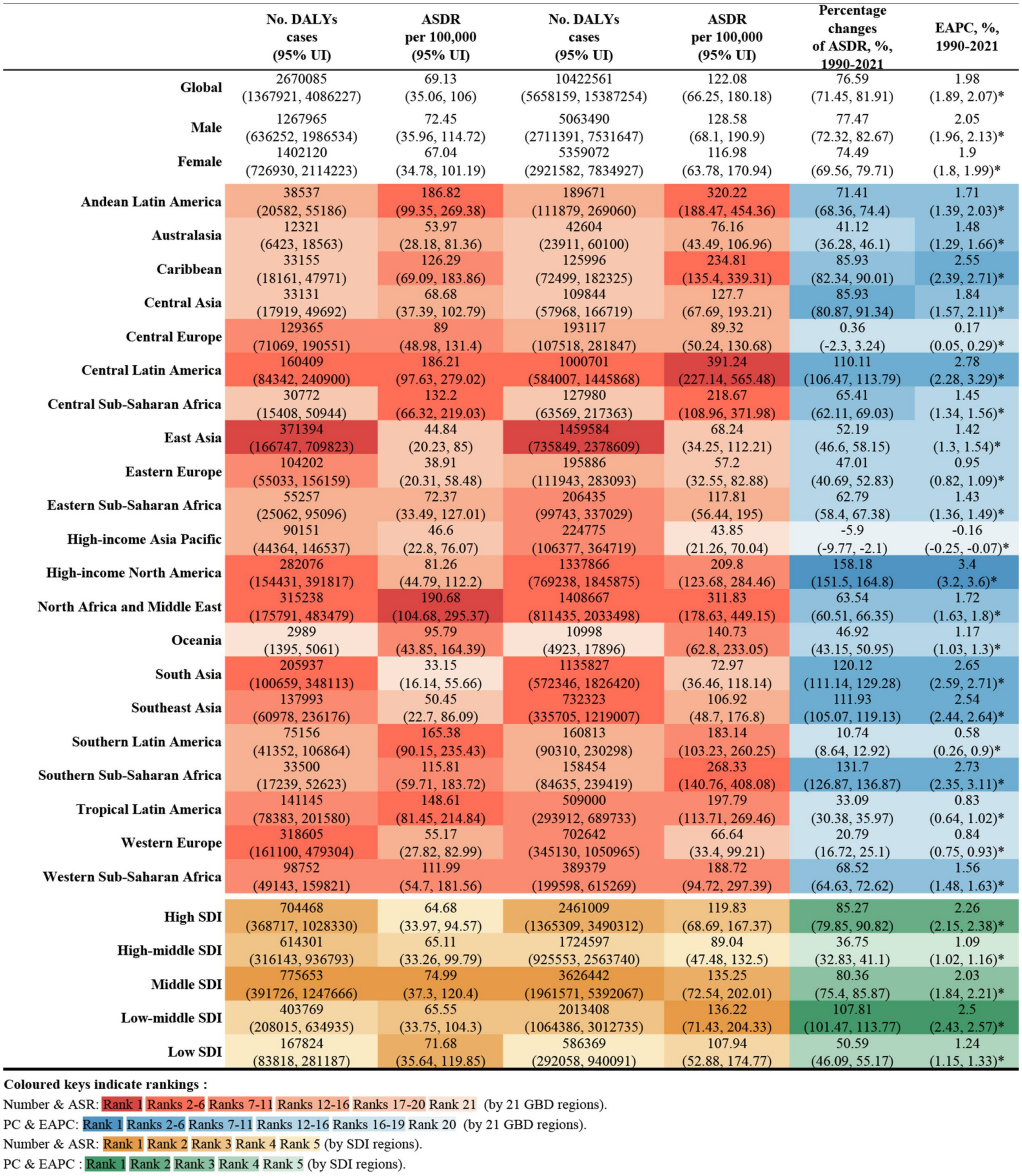
Number of DALYs and ASDR of high BMI-attributable CKD in 1990 and 2021, and the percentage changes and EAPC of ASDR from 1990 to 2021, by sex and location. BMI, body mass index; CKD, chronic kidney disease; DALYs, deaths and disability-adjusted life years; ASDR, age-standardized DALY rate; EAPC, estimated annual percentage change.

In 2021, global deaths from CKD attributed to high BMI were 418,400 (95% UI: 224300–621,300), with 195,600 deaths in males (95% UI: 103200–295,400) and 222,800 deaths in females (95% UI: 118900–327,400) (Figure 1). The global DALYs for CKD caused by high BMI in the same year were 10,422,600 (95% UI: 5658200–15,387,300), with 5,063,500 DALYs in males (95% UI: 2711400–7,531,600) and 5,359,100 DALYs in female (95% UI: 2921600–7,834,900) (Figure 2).

The ASMR for CKD attributable to high BMI in 2021 was 5.06 per 100,000 population (95% UI: 2.70–7.51) globally, with 5.47 per 100,000 for males (95% UI: 2.87–8.30) and 4.77 per 100,000 for female (95% UI: 2.55–7.01). The ASDR was 122.08 per 100,000 population (95% UI: 66.25–180.18), with 128.58 per 100,000 for males (95% UI: 68.10–190.90) and 116.98 per 100,000 for females (95% UI: 63.78–170.94) (Figures 1, 2).

**Figure 3:**
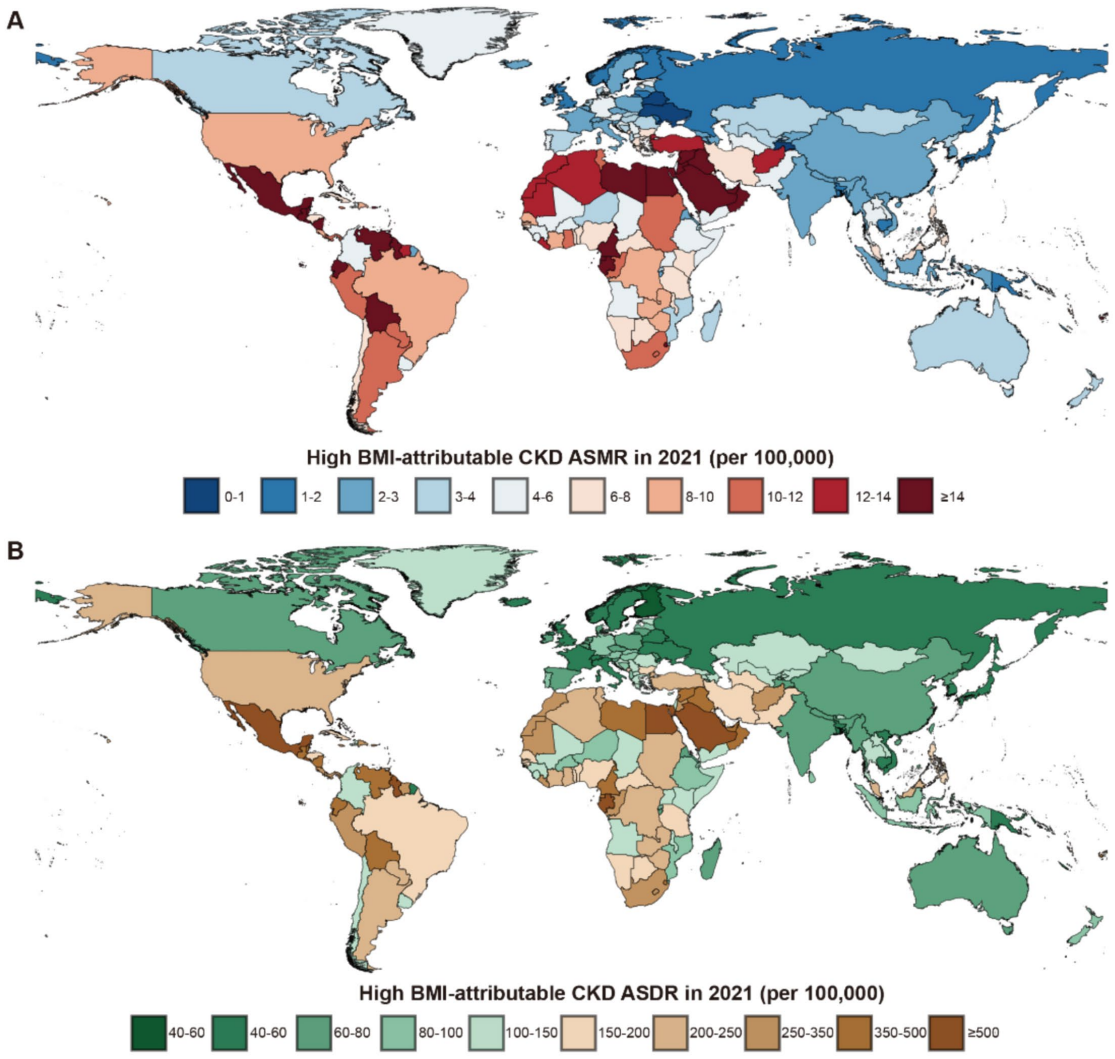
Global burden of high BMI-attributable CKD across 204 countries and territories in 2021. **(A)** The high BMI-attributable CKD ASMR. **(B)** The high BMI-attributable CKD ASDR. BMI, body mass index; CKD, chronic kidney disease; ASMR, age-standardized mortality rate; ASDR, age-standardized DALY rate.

Among the 21 GBD regions in 2021, Central Latin America and Andean Latin America had the highest ASMR and ASDR, indicating the regions with the highest CKD burden attributable to high BMI. In contrast, Eastern Europe and the high-income Asia Pacific exhibited the lowest ASMR and ASDR, reflecting the lightest burden of BMI-attributable CKD (Figure 3).

In the 204 countries of the GBD, a significant correlation was identified between their ASMR and ASDR with SDI (Supplementary Figure S1). The three countries with the highest ASMR were Saudi Arabia in the High SDI group (35.62 per 100,000 population, 95% UI: 19.80–53.06), Egypt in the Low-Middle SDI group (30.74 per 100,000 population, 95% UI: 16.23–45.36), and American Samoa in the High-Middle SDI group (29.53 per 100,000 population, 95% UI: 13.18–48.50). The ASDR for these countries were as follows: Saudi Arabia, 764.26 (95% UI: 444.94–1,126.37); Egypt, 668.90 (95% UI: 321.39–1,029.32); and American Samoa, 611.76 (95% UI: 335.91–896.36) (Supplementary Tables S1, S2).

The notably low ASMR was observed in Ukraine (0.56, 95% UI: 0.27–0.96) in the High-Middle SDI group, while Finland had the lowest ASDR in the High SDI group with 39.78 (95% UI: 19.59–61.12). In China, the ASMR for CKD caused by high BMI in 2021 was 2.83 per 100,000 population (95% UI: 0.72–1.40), while the ASDR was 66.97 per 100,000 population (95% UI: 33.82–109.54) (Supplementary Tables S1, S2).

### Trends in global burden of CKD attributable to high BMI from 1990 to 2021

From 1990 to 2021, the global deaths from CKD attributable to high BMI increased from 92,000 (95% UI: 47200–140,800) to 418,400 (95% UI: 224,300–621,300), while DALYs increased from 2.67 million (95% UI: 1.37–4.09 million) to 10.42 million (95% UI: 5.66–15.39 million), representing an approximate fourfold increase. During this period, the EAPC for global deaths was 2.25% (95% UI: 2.13–2.35), and for DALYs was 1.98% (95% UI: 1.89–2.07) (Figures 1, 2).

**Figure 4:**
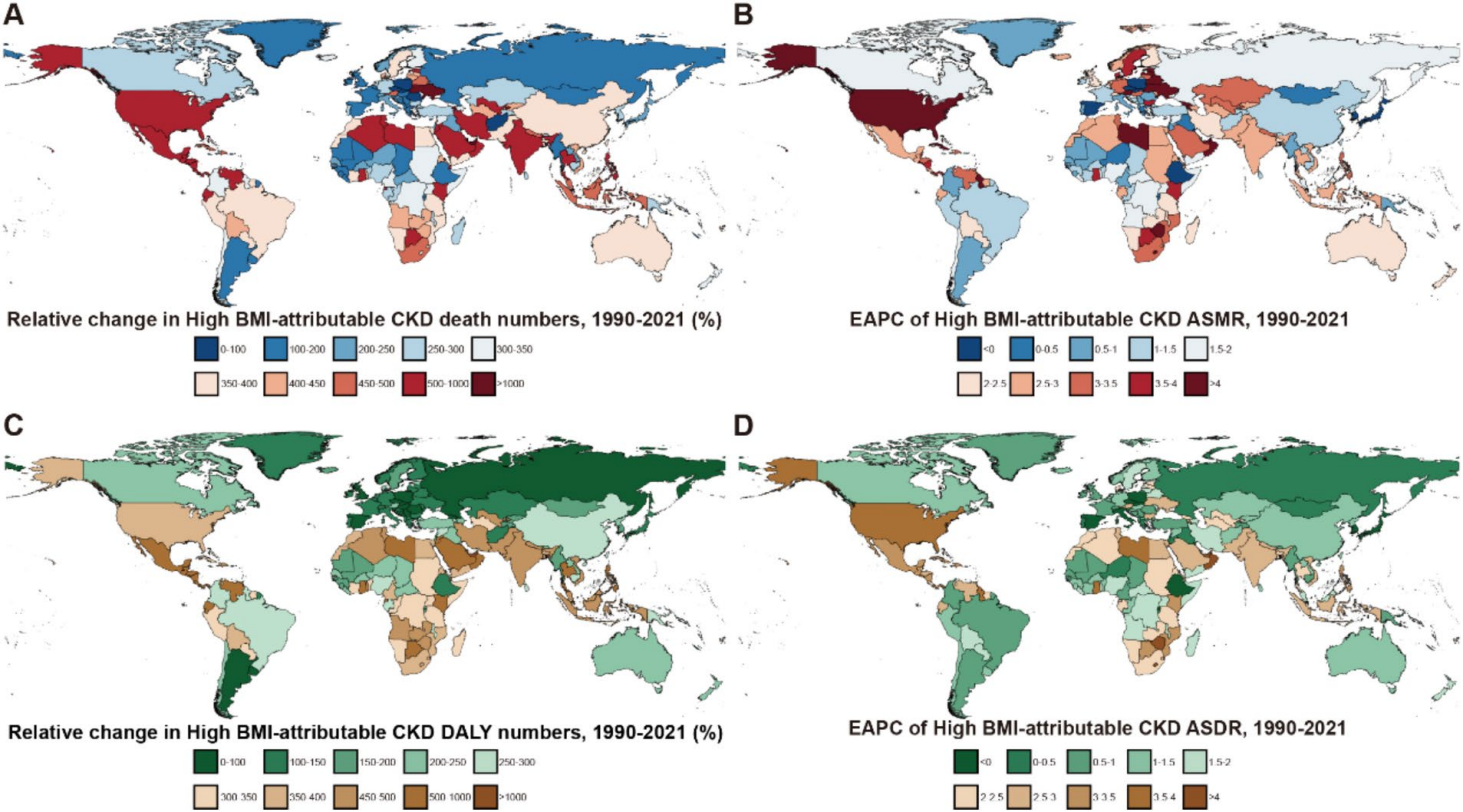
Global burden of high BMI-attributable CKD across 204 countries and territories from 1990 to 2021. **(A)** Relative change in the number of deaths for high BMI-attributable CKD. **(B)** EAPC in ASMR for high BMI-attributable CKD. **(C)** Relative change in DALYs to high BMI-attributable CKD. **(D)** EAPC in ASDR for high BMI-attributable CKD; BMI, body mass index; CKD, chronic kidney disease; ASMR, age-standardized mortality rate; EAPC, estimated annual percentage change; DALYs, disability-adjusted life years; ASDR, age-standardized DALY rates.

Across the 21 GBD regions, both the ASMR and ASDR attributable to high BMI showed an increasing trend (Figure 4). Regions where the EAPC for mortality exceeded the global average (2.25%) include High-Income North America (4.09%), Central Asia (3.31%), Southern sub-Saharan Africa (3.25%), Central Latin America (2.78%), the Caribbean (2.68%), South-East Asia (2.66%), Eastern Europe (2.65%) and South Africa (2.55%). For DALYs, regions with an EAPC above the global average include High-income North America (3.4%), Central Latin America (2.78%), Southern sub-Saharan Africa (2.73%), South Asia (2.65%), the Caribbean (2.55%) and Southeast Asia (2.55%). Notably, High-income Asia Pacific exhibited a negative EAPC of −0.16% (95% UI: −0.25 to 0.07). While Central Asia and Eastern Europe had higher EAPCs for mortality, their EAPCs for DALYs were lower, at 1.84 and 0.95%, respectively (Figures 1, 2).

Trends in deaths and DALYs attributable to high BMI varied among the 204 countries included in the GBD, the countries with the fastest increase in mortality were Ukraine (High-Middle SDI, EAPC: 14.26, 95% UI: 12.24–16.31) and Armenia (Middle SDI, EAPC: 8.7, 95% UI: 7.37–10.06). In contrast, Poland (High SDI) and the Maldives (Middle SDI) showed a significant decreasing trend in mortality, with EAPCs of −1.5% (95% UI: −1.89 – −1.11) and −0.98% (95% UI: −1.17 – −0.78), respectively (Supplementary Tables S3, S4).

Regarding DALY EAPC, the countries with the fastest increases were Lesotho (5.1, 95% UI: 4.58–5.61) in the Low-Middle SDI regions and the United Arab Emirates (4.48, 95% UI: 3.89–5.07) in the High SDI. In contrast, Poland (−1.5, 95% UI: −1.89 – −1.11) and the Maldives (−0.98, 95% UI: −1.17 – −0.78) exhibited a significant decreasing trend in the burden of CKD attributable to the high BMI by 2021. As a representative country of the High-Middle SDI, China showed an EAPC of 1.41% (95% UI: 1.31–1.51) for mortality and 1.44% (95% UI: 1.32–1.56) for DALYs from 1990 to 2021 (Supplementary Table S4).

### Age-specific and sex trends in CKD burden with high BMI

**Figure 5:**
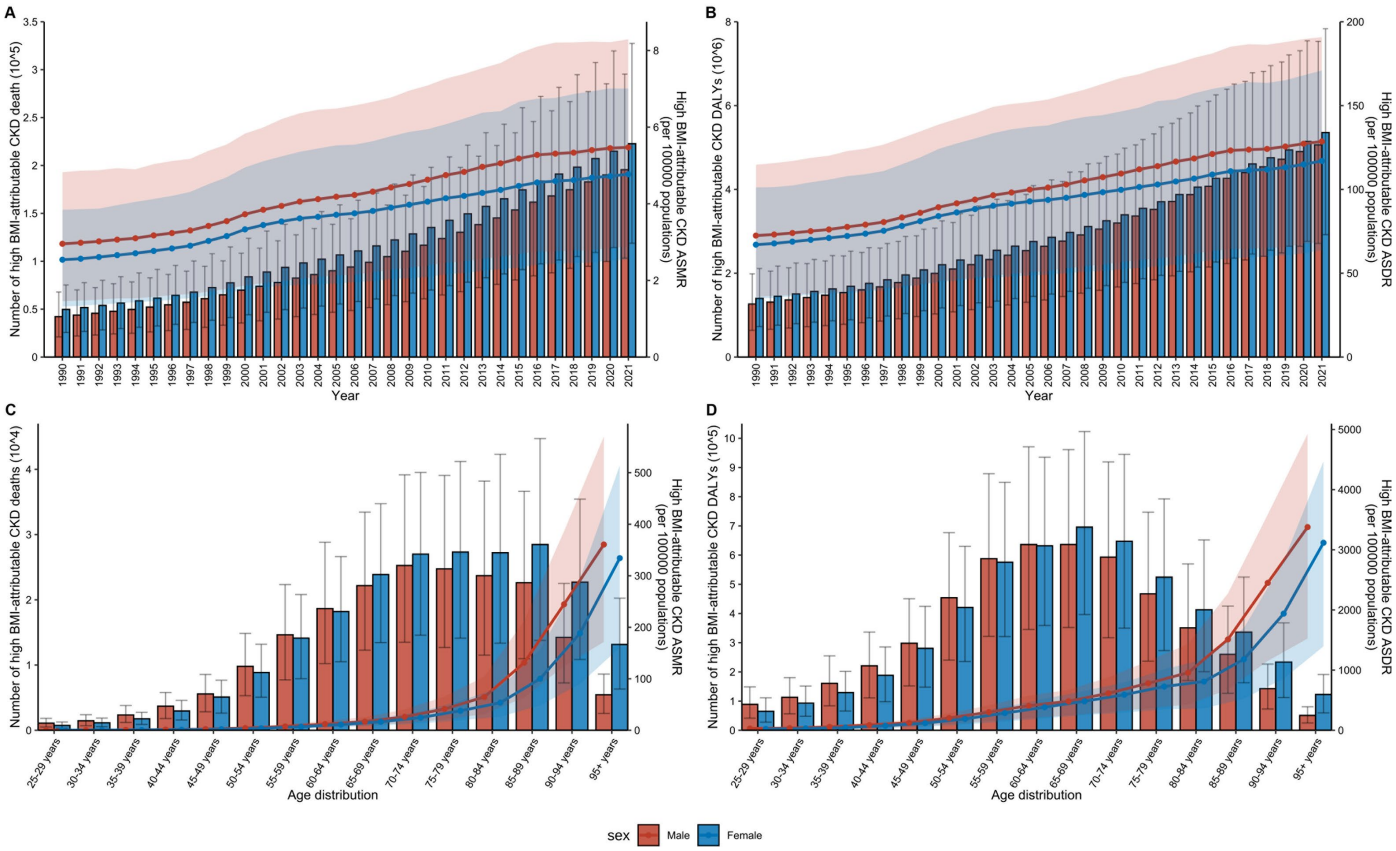
Impact of age and sex on the global burden of high BMI-attributable CKD from 1990 to 2021. **(A)** Temporal trends in the number of deaths and ASMR by sex from 1990 to 2021. **(B)** Temporal trends in the number of DALYs and ASDR by sex from 1990 to 2021. **(C)** Distribution of deaths and ASMR by sex and age group in 2021. **(D)** Distribution of DALYs and ASDR by sex and age group in 2021; BMI, body mass index; CKD, chronic kidney disease; ASMR, age-standardized mortality rate; DALYs, disability-adjusted life years; ASDR, age-standardized DALY rates. The error bars represent 95% uncertainty intervals. The dots represent observed values from the GBD dataset. The solid line represents the mean value. The shaded area denotes the 95% uncertainty interval.

From 1990 to 2021, there was a notable increase in the number of global deaths and DALYs attributable to CKD in individuals with high BMI across all age groups (Figure 5). Throughout the 30 years, mortality and DALY rates were consistently lower among males than females across all age groups, though their ASR for males remained higher than that for females each year (Supplementary Table S5).

In 2021, an analysis of CKD attributable to high BMI across age groups from 25 to over 95 years revealed that both mortality and DALY rates increased with age (Figure 5). The highest number of deaths was in the 70–74 age group, while the highest DALY rate was in the 65–69 age group (Supplementary Table S6). Among females, the highest number of deaths occurred in the 85–89 age group, while the highest DALY was concentrated in the 65–69 age group. In males, the peak number of deaths occurred in the 70–74 age group, while the highest DALY was concentrated in the 60–64 age group (Supplementary Table S6). Overall, CKD attributable to high BMI affected males at a younger age than females globally.

### Impact of high BMI on CKD caused by different etiologies

**Figure 6:**
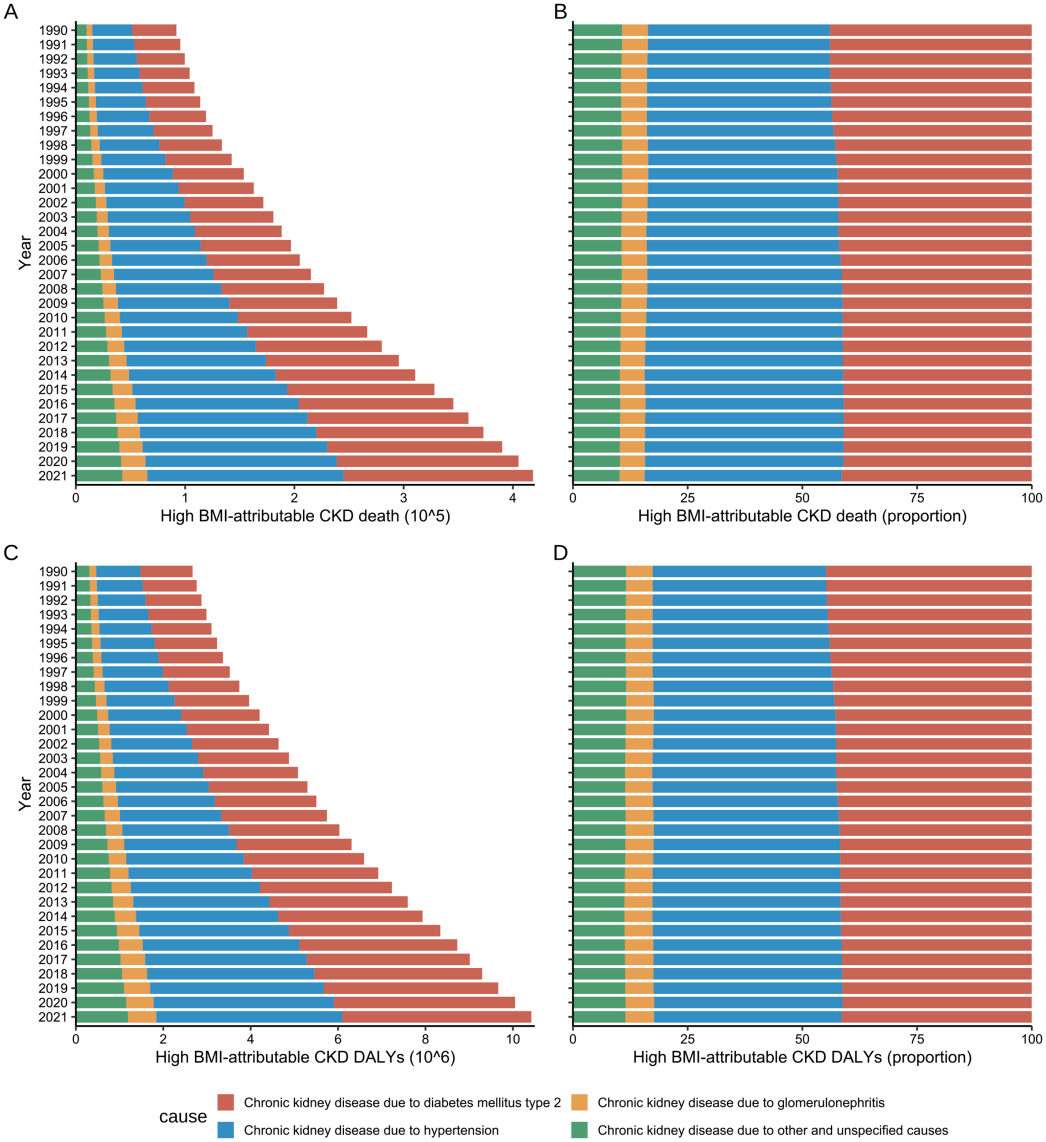
Global number and rate of deaths and DALY for High BMI-attributable CKD by underlying causes from 1990 to 2021. **(A,B)** Temporal trends in the burden of deaths by underlying causes. **(C,D)** Temporal trends in the burden of DALYs by underlying causes; BMI, body mass index; CKD, chronic kidney disease; DALYs, disability-adjusted life years.

From 1990 to 2021, type 2 diabetes mellitus (T2DM), hypertension, and glomerulonephritis were the primary factors contributing to the development of CKD attributable to high BMI (Figure 6). In 2021, globally, T2DM-related CKD was the cause of 179,800 mortalities and 4.32 million DALYs, while hypertension-related CKD caused 173,300 and 4.26 million, respectively (Supplementary Tables S7, S8). Sex differences were evident in the data, with an analysis of global trends across all age groups indicating that from 1990 to 2021, hypertension and T2DM were the leading causes of CKD mortality attributable to high BMI in males (Supplementary Figure S2) and females (Supplementary Figure S3). Notably, the proportion of deaths resulting from these conditions in high BMI-attributable CKD has undergone a shift over the past 30 years.

Before 1994, hypertension and T2DM were the primary causes of CKD mortality attributable to high BMI in males, causing 21,300 (95% UI: 9,700–35,700) and 21,200 (95% UI: 8,900–36,500) mortalities, respectively, representing approximately 42% of the total deaths. Subsequently, following 1995, the proportion of deaths attributed to hypertension in males gradually increased. By 2021, hypertension became the primary cause of CKD mortality attributable to high BMI in males, while accounting for 88,900 (95% UI: 44200–131,300) deaths, surpassing T2DM (79,600, 95% UI: 33900–137,100), and constituted 45.47% of total deaths (Supplementary Table S7).

A comparable trend was identified in the females. In 1990, hypertension and T2DM caused 18,600 (95% UI: 9,100–29,000) and 22,300 (95% UI: 9900–35,900) mortalities, respectively, accounting for 37.51 and 44.88% of the total CKD deaths attributable to high BMI. By 2021, deaths attributed to hypertension had risen to 90,800 (95% UI: 47300–131,900), with its proportion increasing to 40.78%. Conversely, deaths caused by T2DM increased to 93,600 (95% UI: 42100–150,900), although its proportion decreased to 42.03%. Notwithstanding this decline, T2DM remained the primary cause of CKD mortality attributable to high BMI in females (Supplementary Table S7).

Both hypertension and T2DM were found to contribute to the escalating DALY burden of CKD attributable to high BMI during the study period, with trends in DALY exhibiting congruence to those observed in mortality rates. From 1990 to 2021, hypertension was responsible for the greatest DALY burden in males across all age groups (42.05%), while in females, T2DM accounted for the greatest burden (43.71%) (Supplementary Table S8).

### Correlation and decomposition analysis of CKD burden attributable to high BMI in different SDI regions

**Figure 7:**
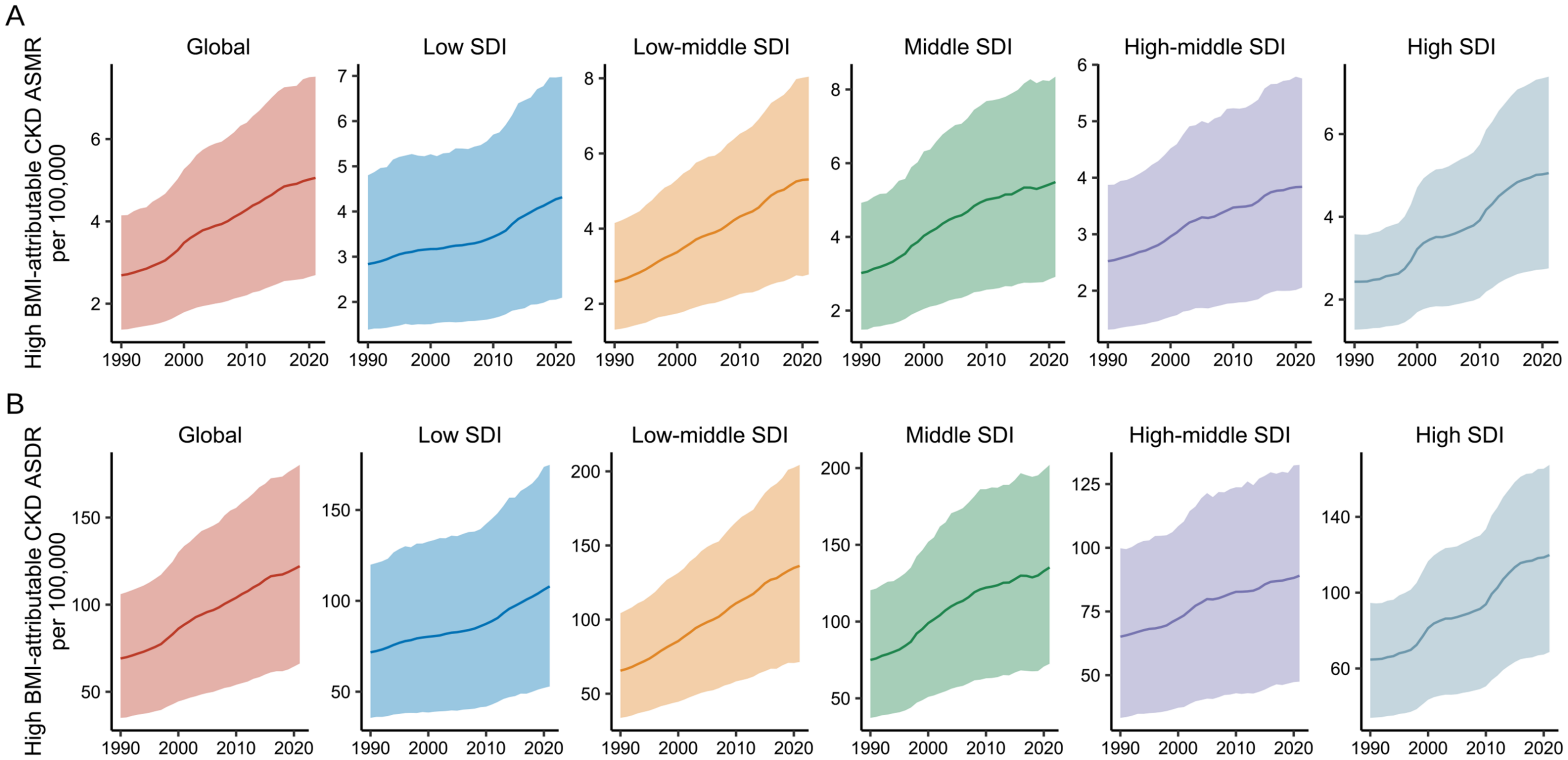
Global burden of high BMI-attributable CKD by SDI from 1990 to 2021. **(A)** Temporal trends in ASMR by SDI from 1990 to 2021. **(B)** Temporal trends in ASDR by SDI from 1990 to 2021. BMI, body mass index; CKD, chronic kidney disease; ASMR, age-standardized mortality rate; ASDR, age-standardized DALY rate; SDI, socio-demographic index. The solid line represents the mean value. The shaded area denotes the 95% uncertainty interval.

At the regional level, both ASMR and ASDR for CKD attributable to high BMI exhibited an increasing trend from 1990 to 2021 (Figure 7). Throughout the study period, the regions with the highest EAPC for CKD attributable to high BMI were High SDI (2.75, 95% UI: 2.6–2.9) and Low-Middle SDI (2.46, 95% UI: 2.39–2.53), with corresponding increases in DALYs of 85.27% (95% UI: 79.85–90.82%) and 107.81% (95% UI: 101.47–113.77%), respectively. In contrast, regions with High-Middle SDI and Low SDI showed the slowest growth in the CKD burden attributable to high BMI over the past 30 years, with the smallest mortality and DALY EAPCs (Figures 1, 2).

**Figure 8:**
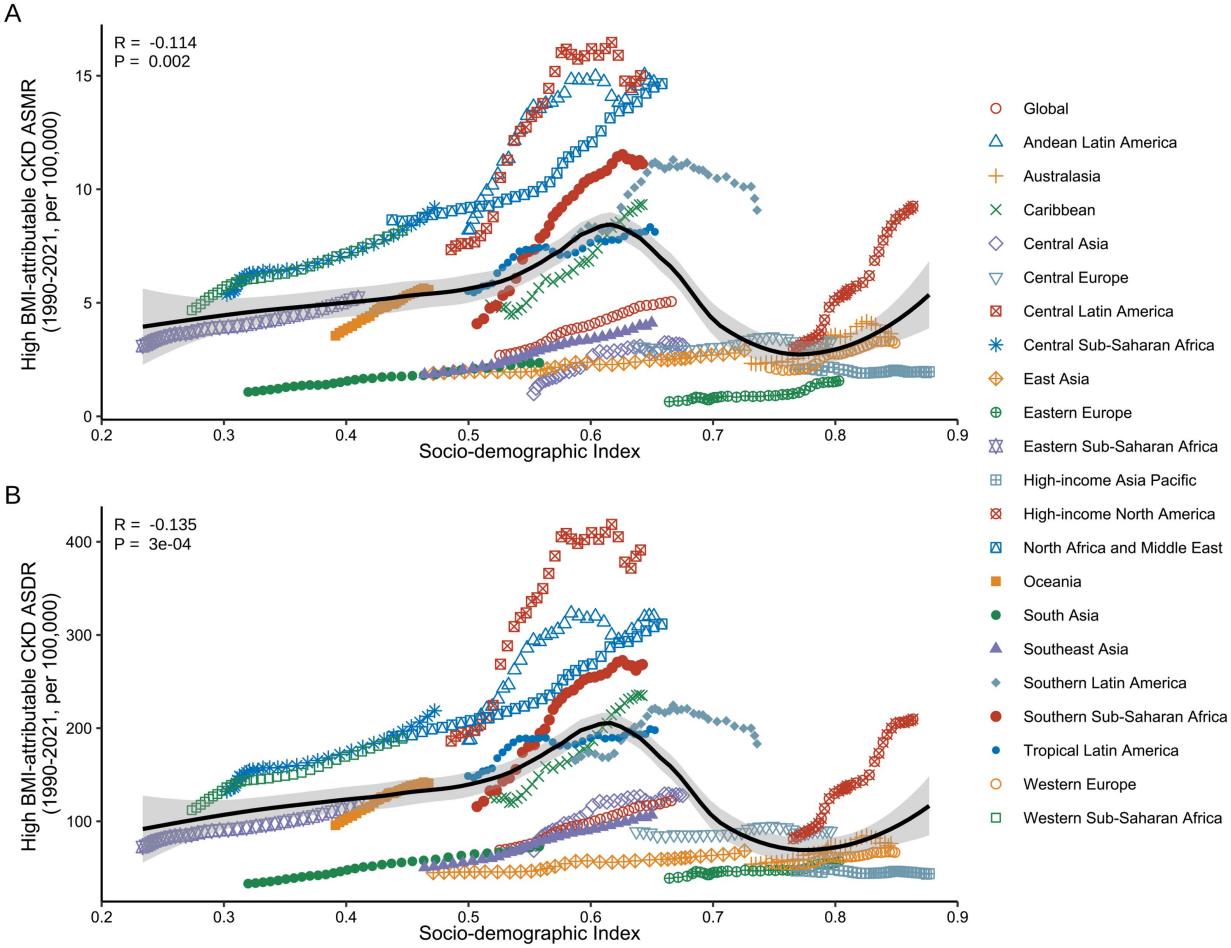
Correlation between ASMR/ASDR for high BMI-attributable CKD and SDI across the 21 GBD regions from 1990 to 2021. **(A)** Association between ASMR of high BMI-attributable CKD and country-level SDI from 1990 to 2021. **(B)** Association between ASDR of high BMI-attributable CKD and country-level SDI from 1990 to 2021. BMI, body mass index; CKD, chronic kidney disease; ASMR, age-standardized mortality rate; ASDR, age-standardized DALY rate; SDI, socio-demographic index. The dots represent observed values from the GBD dataset. The solid line represents the fitted curve from the correlation analysis, while the shaded area indicates the 95% confidence interval.

During the study period, a significant non-linear correlation was observed between ASMR/ASDR and SDI in the 21 GBD regions (Figure 8). In Low SDI (0.2 < SDI < 0.4), ASMR and ASDR remained relatively stable over the 30 years. In the Low-Middle SDI (0.4 < SDI < 0.6) and High SDI (SDI > 0.8), both ASMR and ASDR exhibited an upward trend with increasing SDI. In contrast, in the Middle SDI and High-Middle SDI (0.6 < SDI < 0.8), ASMR and ASDR showed a decreasing trend, with an almost linear decline as SDI improved. Notably, regions such as Central Sub-Saharan Africa (Low-Middle SDI) and High-Income North America (High SDI) experienced a significant increase in CKD burden attributable to high BMI from 1990 to 2021 (Supplementary Table S9).

**Figure 9:**
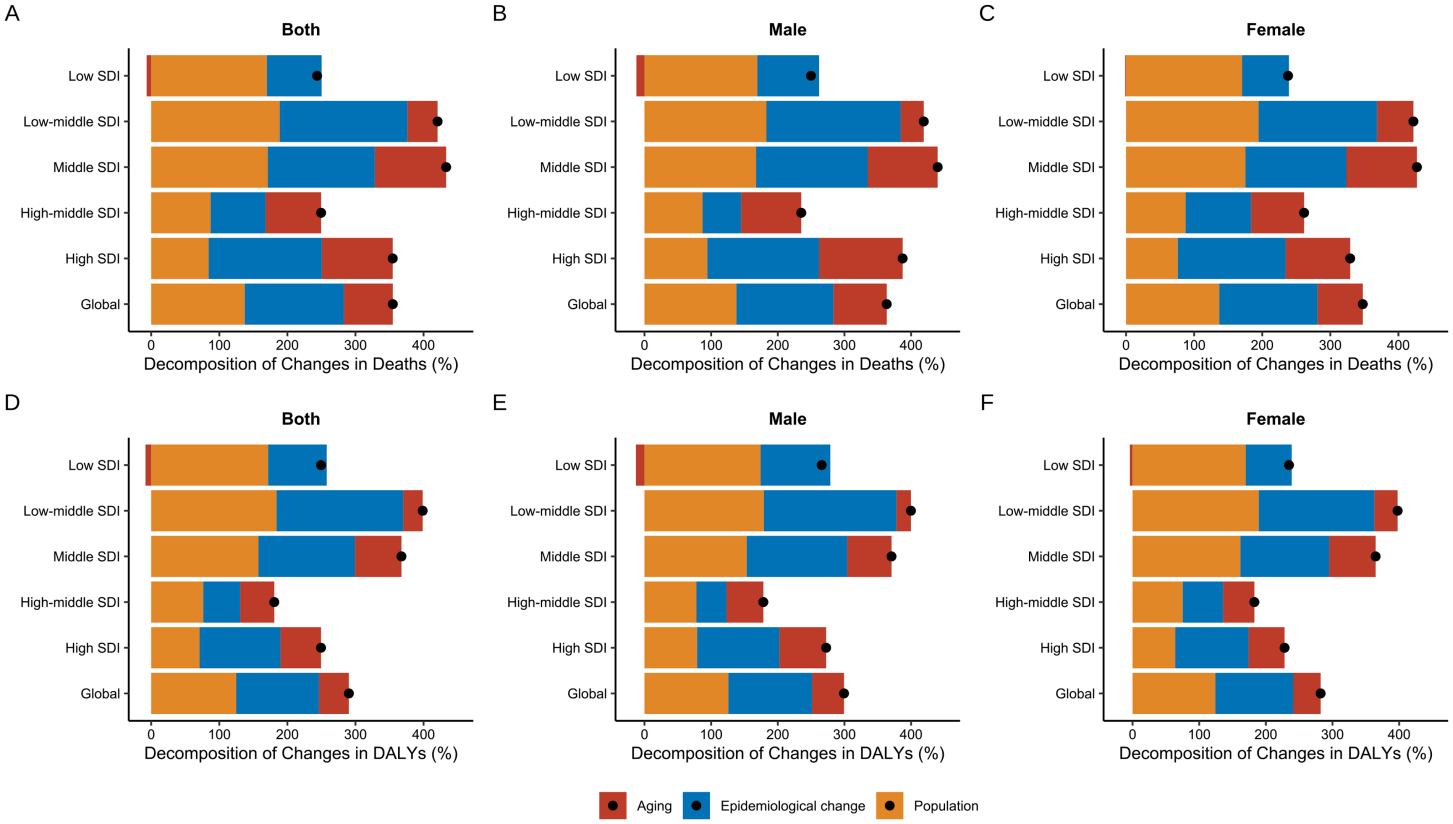
Decomposition analysis of changes in high BMI-attributable CKD from 1990 to 2021. **(A,D)** Decomposition of changes in deaths and DALYs for both sexes respectively. **(B,E)** Decomposition of changes in deaths and DALYs for males respectively. **(C,F)** Decomposition of changes in deaths and DALYs for females, respectively. BMI, body mass index; CKD, chronic kidney disease; ASMR, age-standardized mortality rate; ASDR, age-standardized DALY rate; DALYs, deaths and disability-adjusted life years; SDI, socio-demographic index. The dots represent the net percentage change.

Based on the above analysis, we conducted a decomposition analysis of the changes in mortality and DALY attributed to high BMI-related CKD from 1990 to 2021 (Figure 9). Overall, the aging, epidemiological changes, and population growth contributed to increases in mortality by 72.04, 145.45, and 137.36% globally, respectively (Supplementary Table S10). In Low SDI regions, the increase in high BMI-related CKD mortality was the lowest, with an overall rise of 243.64%. This phenomenon was primarily attributable to the positive effects of population growth and epidemiological changes, while the negative impact of aging was −6.27%. In contrast, all three factors had positive effects in other regions (Figure 9A). Significant sex differences were observed, especially in Low SDI regions, where the negative contribution of aging to male mortality was −12.16%, while the contribution for females was only −1.36% (Figures 9B,C). Furthermore, the effects of aging, epidemiological changes, and population growth on the DALY burden of high BMI-related CKD were consistent with the trends in mortality, with aging showing a negative contribution only in Low SDI regions (Figure 9D).

### Future projections of the CKD burden attributable to high BMI

**Figure 10:**
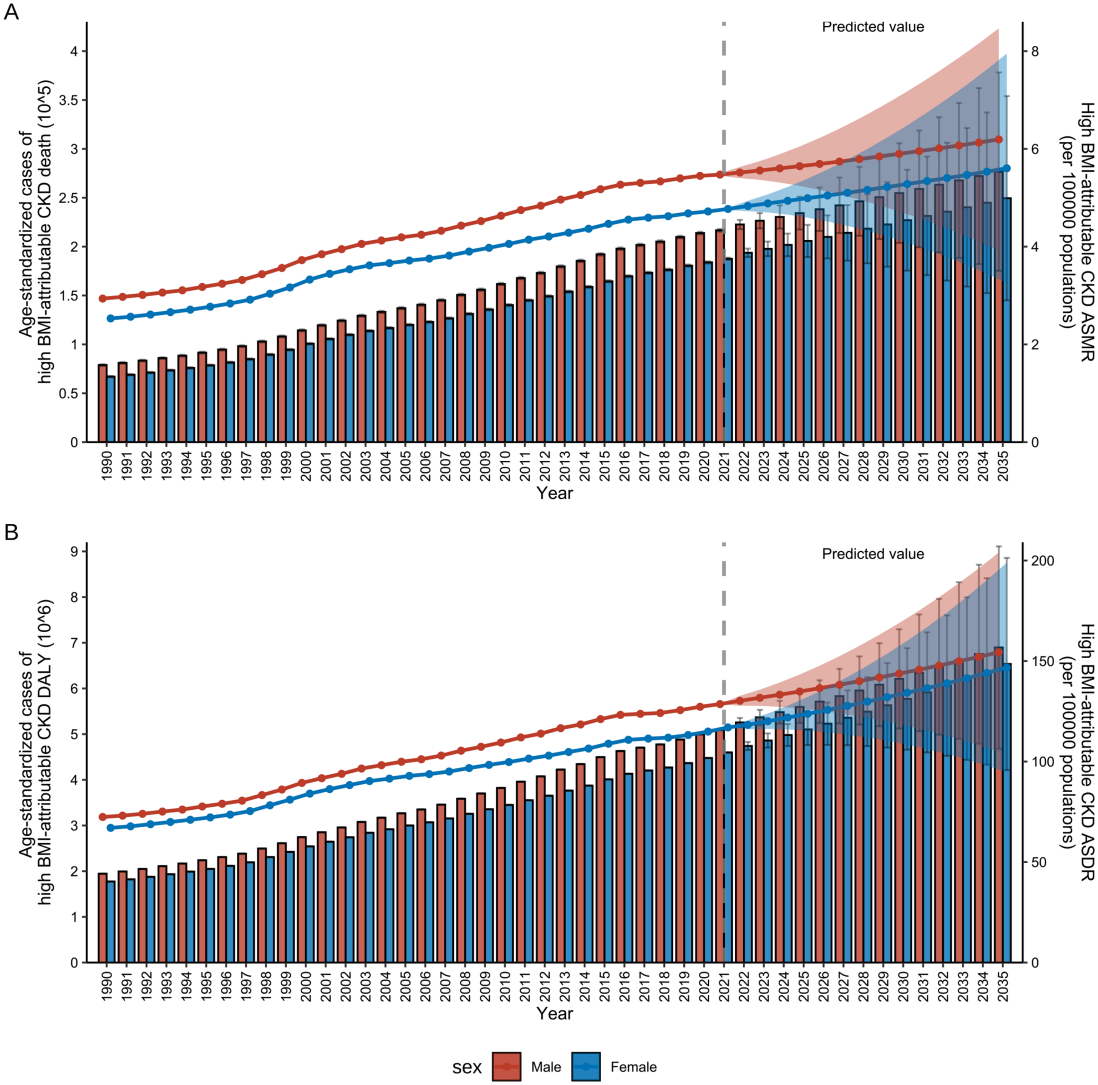
References and projections of high BMI-attributable CKD burden from 1990 to 2035 by sex. **(A)** References and projections of deaths and ASMR by sex. **(B)** References and projections in DALYs and ASDR by sex. BMI, body mass index; CKD, chronic kidney disease; ASMR, age-standardized mortality rate; ASDR, age-standardized DALY rate; DALYs, deaths and disability-adjusted life years. The error bars represent 95% uncertainty intervals. The dots represent predicted values. The shaded area denotes the 95% highest density interval of prediction values. The solid line represents the predicted mean value, and the vertical dashed line marks the starting point of the prediction.

Figure 10 presented both actual and projected data for the CKD burden attributable to high BMI by sex from 1990 to 2035, including ASMR and ASDR. By 2035, global ASMR and ASDR will reach 5.81 (3.55–8.07) per 100,000 individuals and 149.42 (99.45–199.39) per 100,000 individuals, respectively (Figure 10). Overall, both ASMR and ASDR for males and females across all age groups had demonstrated a significant upward trend over the past three decades, reflecting the growing global impact of high BMI on CKD. For males, the ASDR increased from 72.42 per 100,000 (95% UI: 72.29–72.56) in 1990 to 128.58 per 100,000 (95% UI: 128.47–128.69) in 2021 and it was projected to reach 154.38 per 100,000 (95% UI: 104.83–203.93) by 2035 (Supplementary Table S11). In females, the ASDR increased from 67.02 per 100,000 (95% UI: 66.91–67.13) in 1990 to 116.98 per 100,000 (95% UI: 116.88–117.08) in 2021 and is projected to reach 146.77 per 100,000 (95% UI: 94.65–198.88) by 2035 (Supplementary Table S11). The growth rates of ASMR mirror those of ASDR, exhibiting an overall increasing trend. Notably, over the next 15 years, both male and female ASMR are projected to stabilize at a rate of 5–6 per 100,000 (Supplementary Table S12).

Furthermore, the analysis of actual and projected data for the CKD burden attributable to high BMI by sex for all ages from 1990 to 2035, revealed that both ASDR and ASMR were found to be an age-dependent pattern in males (Supplementary Figure S4) and females (Supplementary Figure S5). By 2035, the ASDR and ASMR for the age group 55–74 years showed no significant difference between the sexes. However, for those aged 75 and older, the burden for males will be significantly higher than for females (Supplementary Tables S13–S16).

## Discussion

The CKD has emerged as a major global health concern, currently affecting over 800 million individuals, which constitutes more than 10% of the global population (24). By 2040, CKD is projected to become the fifth leading cause of death worldwide, a trend that has been attributed primarily to the rising prevalence of obesity (12). High BMI, a modifiable and independent risk factor for non-communicable diseases, plays a pivotal role in shaping the global CKD burden (25). Previous studies have estimated that approximately 2.7% of CKD cases were directly attributable to obesity, with high BMI potentially accounting for 15–30% of the global CKD burden (26). However, prior research has primarily focused on general associations between obesity and kidney dysfunction, with limited insights into the attributable CKD burden across countries, sexes, and SDI. This study addresses this gap by providing a comprehensive, comparative analysis of the CKD burden attributable to high BMI with the last GBD 2021 data.

The findings revealed a significant increase in the global burden of CKD attributable to high BMI from 1990 to 2021, with mortality rising more than fourfold. This upward trend mirrored the expanding impact of high BMI on chronic disease morbidity and mortality worldwide (27, 28). Importantly, this study not only reaffirmed the established link between BMI and CKD outcomes but also uncovered new demographic and epidemiological patterns that have been previously underexplored.

Both sexes experienced a marked increase in CKD mortality and DALYs attributable to high BMI across all age groups, although the patterns diverged by sex. While the absolute number of deaths and DALYs was higher among females, the ASR rose more significantly in males. This divergence may reflect sex-specific differences in health behaviors, occupational exposures, and engagement with preventive care. Among females, the relatively stable ASR may reflect protective hormonal factors, longer life expectance, or differences in health-seeking behavior. These sex-specific patterns underscore the importance of sex-responsive public health strategies.

The age-specific analysis further revealed that males bore a higher mortality and DALY burden before age 65, while females surpassed males thereafter. This may be explained by demographic structures and sex-based differences in risk exposure and longevity, with significant implications for disease mechanisms and health management (29). Despite a higher absolute burden, females exhibited lower ASR, which may have been attributed to their longer life expectancy and a larger proportion of elderly individuals (30). Moreover, males often engage in higher-risk behaviors, such as smoking, and alcohol consumption, and have higher early mortality (31), which contributes to elevated ASR in middle-aged groups. Conversely, postmenopausal females may become more vulnerable to CKD with estrogen loss, and BMI-induced metabolic disturbances (32, 33). In response to these challenges, the WHO has implemented novel strategies to promote sex health equality (34). This study also identified disease-specific patterns in the etiology of BMI-related CKD. Among males, hypertension emerged as the leading cause of BMI-attributable CKD mortality by 2021, Whereas T2DM remained the predominant cause among females. These patterns reflect the differential metabolic responses and risk factor prevalence across sexes and age groups, further supporting the need for age- and sex-tailored prevention efforts.

Geographic variation in BMI-related CKD burden was also substantial across SDI regions. Unexpectedly, high and high-middle SDI regions, where obesity epidemics have plateaued (25), still experienced increasing BMI-attributable CKD mortality. This paradox may be partially explained by improved CKD detection, greater disease awareness, and the delayed effects of cumulative metabolic risk (35). In addition, factors such as evolving diagnostic criteria, greater access to healthcare, and intensive screening programs may also contribute to the observed trend (36). Countries such as the United Arab Emirates and Austria exhibited some of the highest EAPCs, likely reflecting the influence of westernized dietary habits, sedentary lifestyles, and aging populations (37–40). Nevertheless, countries such as Japan, South Korea, Poland, and Kuwait, in high SDI regions, successfully reduced CKD-related DALYs through robust public health policies, lifestyle interventions, and expanded access to preventive care (41–44). Notably, Central Latin America and Andean Latin America, classified as high-middle SDI regions, exhibited disproportionately high ASMR and ASDR. These patterns may be attributed to a combination of factors, including a high prevalence of obesity-related comorbidities, limited healthcare resources, environmental and occupational exposures, and insufficient public healthcare systems (45). This illustrated that areas with a high-middle SDI may experience a more severe double burden during the health transition period. Meanwhile, low- and low-middle SDI regions, including Lesotho, Zimbabwe, and El Salvador, reported persistently high and increasing burdens.

These regions face dual challenges: rising exposure to obesogenic environments driven by urbanization, alongside limited healthcare infrastructure, weak surveillance systems, and insufficient public health investments (46, 47). The pronounced increase in DALYs in these regions emphasizes the urgent need for health system strengthening and international cooperation. Projections indicate that the global burden of high BMI-attributable CKD will continue to rise through 2035, demanding immediate and sustained global action. Currently, global efforts to prevent and address obesity remain inefficient and fragmented, underscoring the need for structural reforms at the systemic level. To effectively reduce the health risks associated with high BMI, international health regulations and national policy changes must be enhanced, particularly in obesity prevention, promotion of physical activity, and healthy dietary habits (48, 49).

A unique feature of this study was the inclusion of data from 2020 to 2021, years profoundly impacted by the COVID-19 pandemic. The pandemic healthcare services, chronic disease management, and data collection, particularly in low-resource settings. Additionally, behavioral changes during lockdowns, such as increased sedentary time, dietary changes, and delayed care for non-communicable diseases, likely exacerbated obesity and CKD risks. Although the GBD 2021 methodology adjusts for these confounders, interpreting trends during this period requires caution. The pandemic may have led to underreporting of CKD diagnoses, misclassification of causes of death, and short-term fluctuations in risk exposure. Nonetheless, the findings presented here serve as critical baseline data for guiding post-pandemic public health recovery efforts.

The growing recognition of obesity as a major contributor to CKD has intensified interest in novel therapeutic strategies. Glucagon-like peptide-1 receptor agonist (GLP-1RAs), including semaglutide, have demonstrated reno-protective effects beyond weight-reducing and glycemic control (50). Clinical trials have shown that GLP-1RAs slow CKD progression and reduce albuminuria in patients with obesity and T2DM, with mechanisms involving anti-inflammatory action, improved endothelial function, and favorable renal hemodynamics (50–52). Incorporating such pharmacotherapies into global obesity management may play a pivotal role in reducing BMI-attributable CKD burden.

Despite its strengths, including the use of high-quality, standardized global data spanning three decades, several limitations must be acknowledged. First, the data from low-income or conflict-affected regions may be incomplete or unreliable, particularly in earlier years. Second, regional disparities in CKD diagnostic criteria, healthcare access, and reporting quality may introduce regional bias. Third, although BMI, has widespread application in metabolic disease studies, it does not differentiate between fat and muscle mass or account for fat distribution, which may vary by age, sex, and ethnicity. Consequently, reliance on BMI alone may not fully capture the obesity-related CKD risk across diverse populations. Lastly, the absence of ethnic-specific data in the GBD framework precluded stratified analysis by race or ethnicity, which may have masked important disparities. Future research should incorporate more refined measures of adiposity metrics, such as waist-to-hip ratio or visceral fat content, and explore ethnically disaggregated data to improve the precision and equity of global CKD burden assessments.

In conclusion, this study provided a comprehensive assessment of the global, regional, and national burden of CKD attributable to high BMI from 1990 to 2021, with projections to 2035. The results demonstrated a sustained and unequal rise in CKD mortality and DALYs attributable to high BMI, disproportionately affecting certain regions, sexes, and age groups. The findings indicated the critical necessity for a globally coordinated, locally adapted strategy to combat obesity and reduce CKD risk. Policymakers should prioritize obesity prevention, CKD screening, and investment in health system resilience, especially in low- and middle-income countries, through data-informed and equitable public health interventive.

## Data Availability

The original contributions presented in the study are included in the article/Supplementary material, further inquiries can be directed to the corresponding authors.

## Footnotes

^1^ https://vizhub.healthdata.org/gbd-results/
