# Supplementary material for: Global burden and trends of high BMI-attributable chronic kidney disease: a comprehensive analysis from 1990 to 2021 and projections to 2035
Source: Front Nutr. 2025 Jul 22;12:1611227. doi: 10.3389/fnut.2025.1611227 (PMC12330218; doi:10.3389/fnut.2025.1611227)
Supplement: Supplementary file 1 [file Data_Sheet_1.PDF]

Global Burden and Trends of High BMI-Attributable Chronic Kidney Disease: A Comprehensive Analysis from 1990 to 2021 and Projections to 2035

Huifang Tan[a, 1†], PhD, Zhifu Liu[b, 2†], PhD, Yongjie Zhang[a], BS, Kehao Yang[b], MM, Yiming Zeng[b], MM, Guoli Li[a], PhD, Zheng Xiao[a], MD, Yuanwei Li[b,*], MD, Yinyin Chen[a,*], MD

[a] Department of Nephrology and Laboratory of Kidney Disease, Hunan Provincial People's Hospital, The First Affiliated Hospital of Hunan Normal University, Changsha, China

[b] Department of Urology, Hunan Provincial People's Hospital, The First Affiliated Hospital of Hunan Normal University, Changsha, China

[†]**These authors contributed equally to this work and share the first authorship**: Huifang Tan and Zhifu Liu.

[*]**Correspondence**:

Yinyin Chen, Department of Nephrology, Hunan Provincial People's Hospital, The First Affiliated Hospital of Hunan Normal University, No. 61 Jiefang West Rd, Changsha, Hunan 410000, P.R. China. Email: chenyinyin1212@hunnu.edu.cn

Yuanwei Li, Department of Urology, Hunan Provincial People's Hospital, The First Affiliated Hospital of Hunan Normal University, 61 Jiefang West Rd, Changsha, Hunan 410000, P.R. China. Email: liyuanwei@hunnu.edu.cn

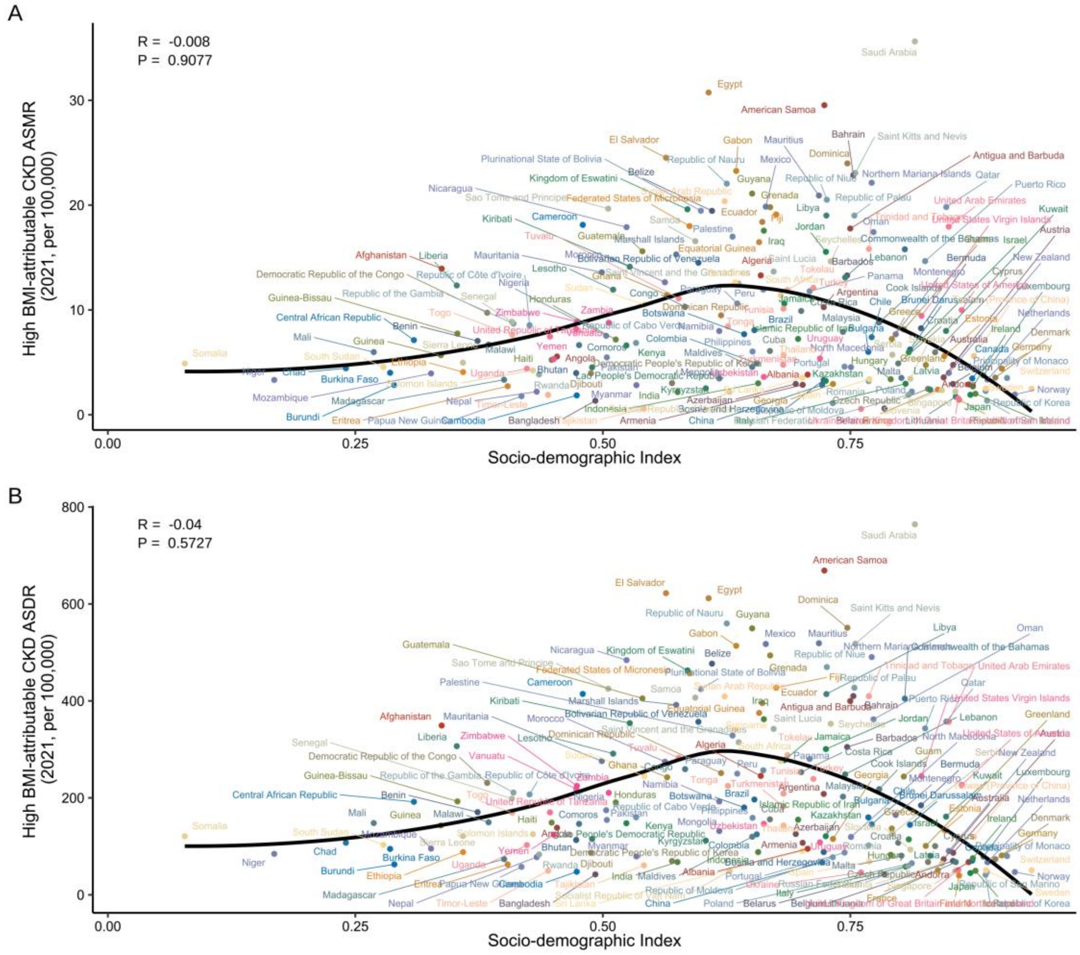

Fig. S1. Association between high BMI-attributable CKD burden and country-level SDI in 2021.

A: ASMR of high BMI-attributable CKD with country-level SDI in 2021; B: ASDR of high BMI-attributable CKD with country-level SDI in 2021.

BMI: Body Mass Index; CKD: chronic kidney disease; ASMR: Age-Standardized Mortality Rate; ASDR: Age-Standardized DALY Rate.

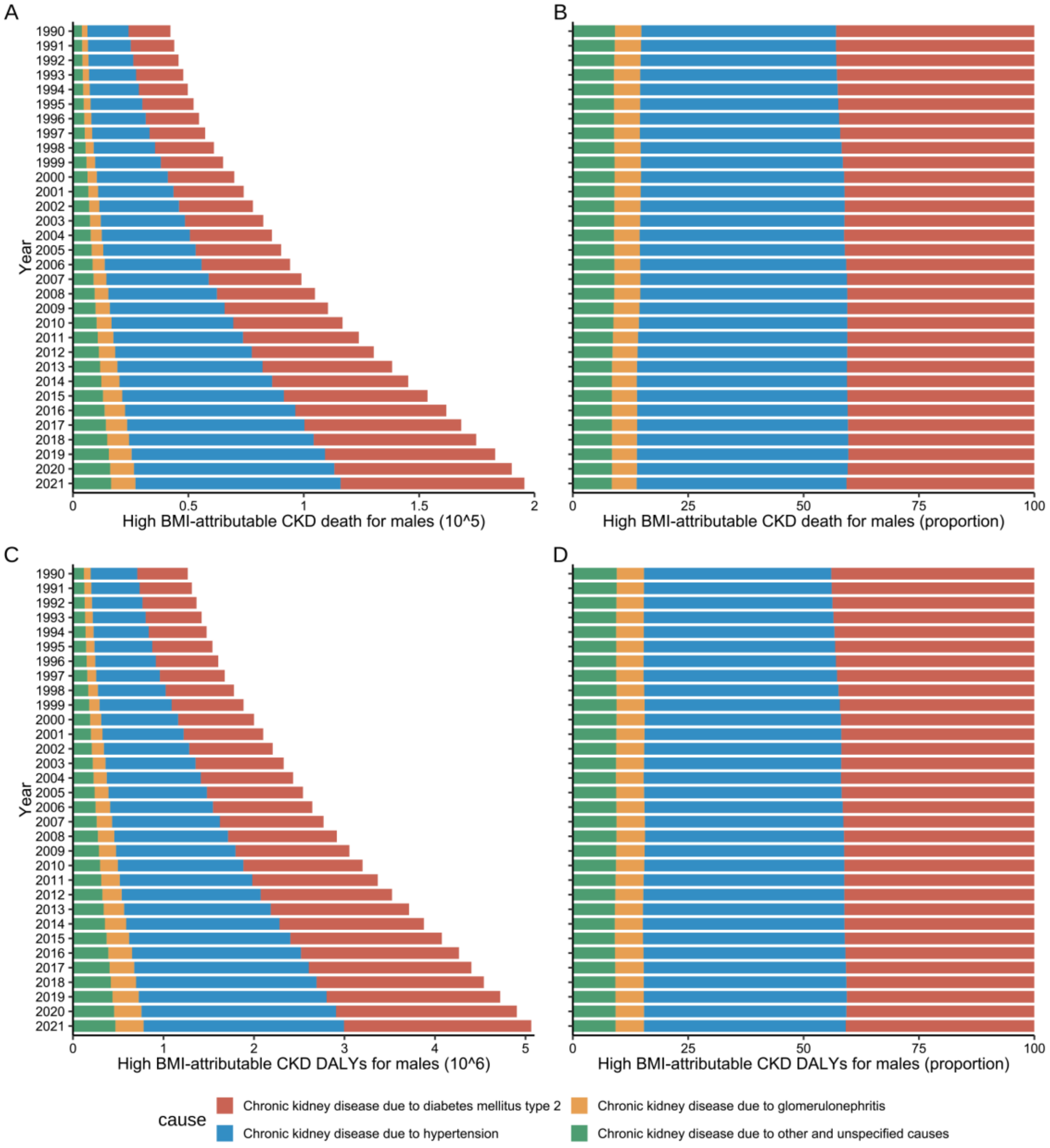

Fig. S2 Number and rate of global death and DALY for High BMI-attributable CKD by underlying cause from 1990 to 2021 in males.

A, B: Temporal trends in the death burden by underlying cause for males from 1990 to 2021; C, D: Temporal trends in DALYs burden by underlying cause for males from 1990 to 2021;

BMI: Body Mass Index; DALYs: Disability-Adjusted Life Years; CKD=chronic kidney disease.

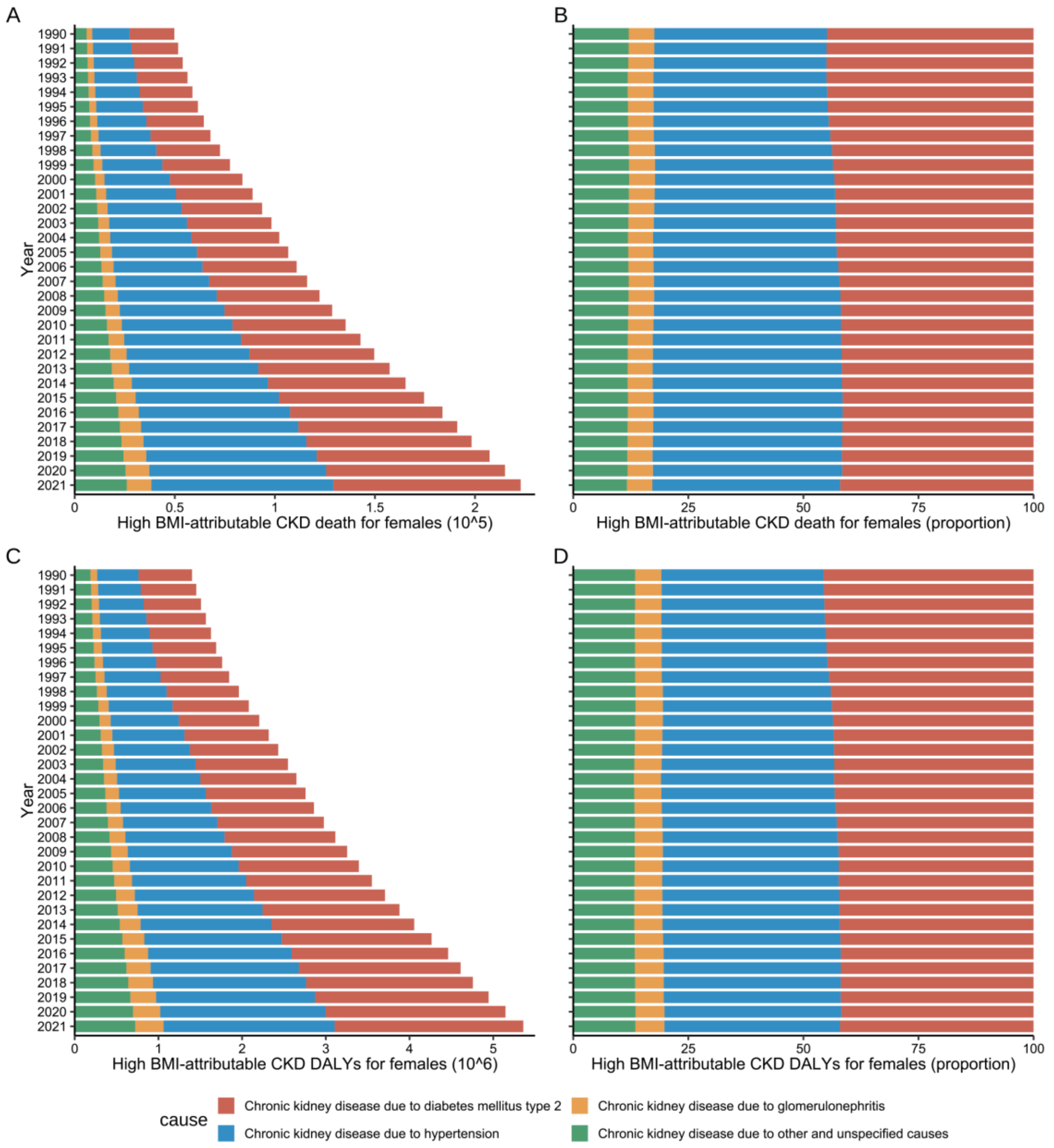

Fig. S3 Number and rate of global death and DALY for High BMI-attributable CKD by underlying cause from 1990 to 2021 in females.

A, B: Temporal trends in the death burden by underlying cause for females from 1990 to 2021; C, D: Temporal trends in the DALYs burden by underlying cause for females from 1990 to 2021;

BMI: Body Mass Index; DALYs: Disability-Adjusted Life Years; CKD=chronic kidney disease.

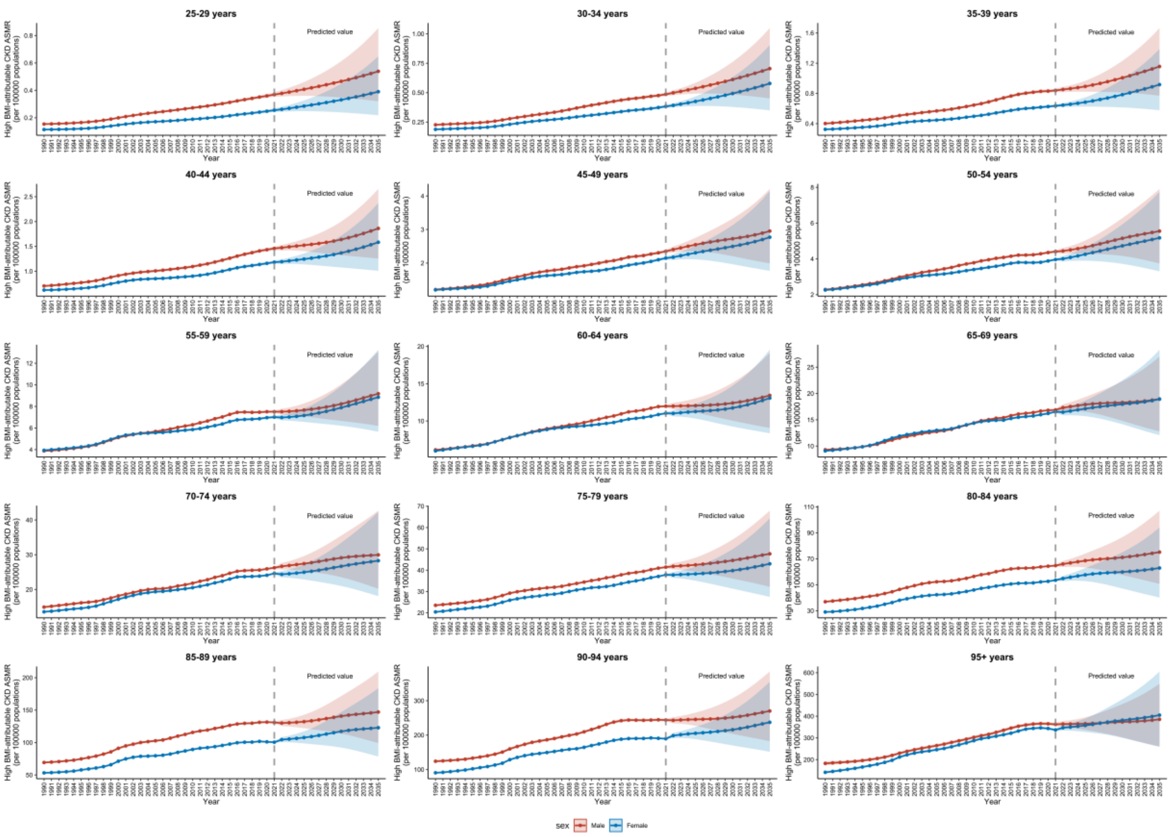

Fig. S4 References and projections of high BMI-attributable CKD ASMR burden from 1990 to 2035 in males.

BMI: Body Mass Index; ASMR: Age-Standardized Mortality Rate; CKD: chronic kidney disease.

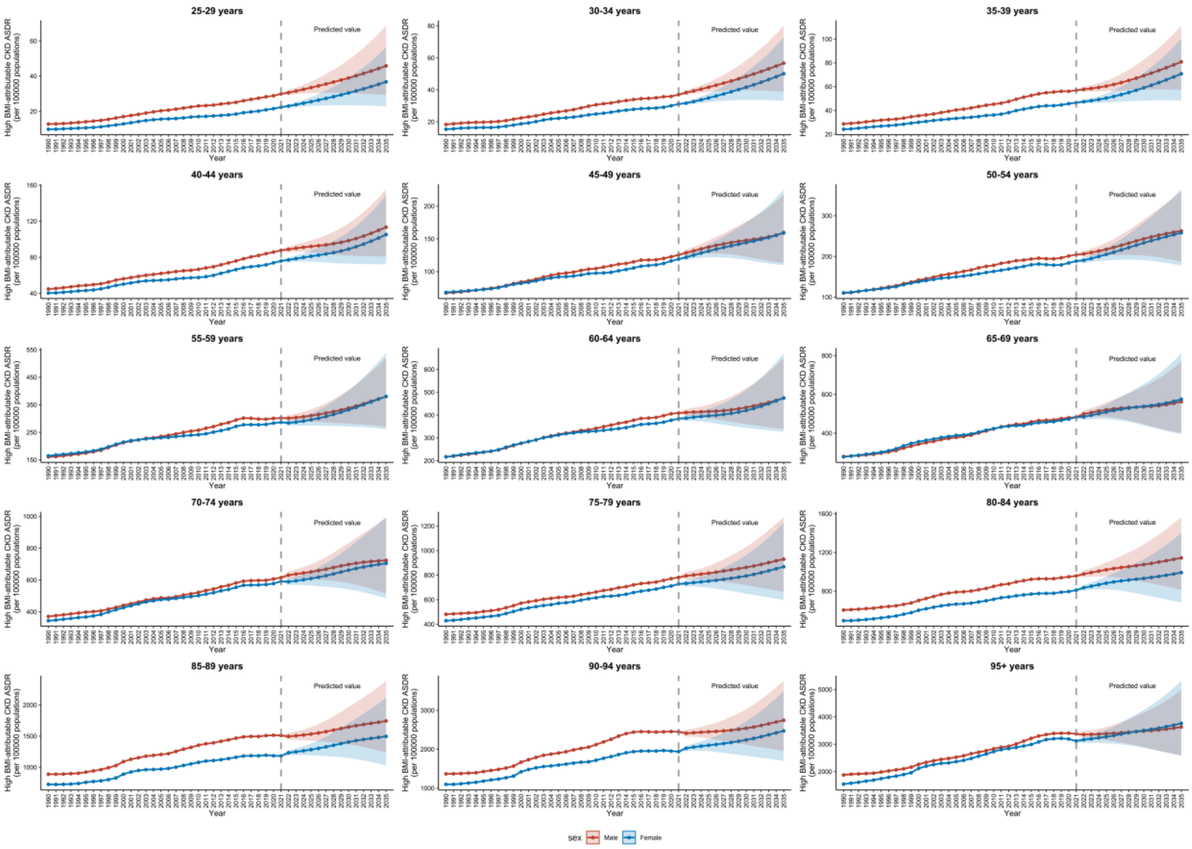

Fig. S5 References and projections of high BMI-attributable CKD ASDR burden from 1990 to 2035 in females.

BMI: Body Mass Index; ASDR: Age-Standardized DALY Rate; CKD: chronic kidney disease.
