# Supplementary material for: Global burden and trends of high BMI-attributable chronic kidney disease: a comprehensive analysis from 1990 to 2021 and projections to 2035
Source: Front Nutr. 2025 Jul 22;12:1611227. doi: 10.3389/fnut.2025.1611227 (PMC12330218; doi:10.3389/fnut.2025.1611227)
Supplement: Supplementary file 2 [file Data_Sheet_2.PDF]

Global Burden and Trends of High BMI-Attributable Chronic Kidney Disease: A Comprehensive Analysis from 1990 to 2021 and Projections to 2035

Huifang Tan[a, 1†], PhD, Zhifu Liu[b, 2†], PhD, Yongjie Zhang[a], BS, Kehao Yang[b], MM, Yiming Zeng[b], MM, Guoli Li[a], PhD, Zheng Xiao[a], MD, Yuanwei Li[b,*], MD, Yinyin Chen[a,*], MD

[a] Department of Nephrology and Laboratory of Kidney Disease, Hunan Provincial People's Hospital, The First Affiliated Hospital of Hunan Normal University, Changsha, China

[b] Department of Urology, Hunan Provincial People's Hospital, The First Affiliated Hospital of Hunan Normal University, Changsha, China

[†]**These authors contributed equally to this work and share the first authorship:** Huifang Tan and Zhifu Liu.

[*]**Correspondence:**

Yinyin Chen, Department of Nephrology, Hunan Provincial People's Hospital, The First Affiliated Hospital of Hunan Normal University, No. 61 Jiefang West Rd, Changsha, Hunan 410000, P.R. China. Email: chenyinyin1212@hunnu.edu.cn

Yuanwei Li, Department of Urology, Hunan Provincial People's Hospital, The First Affiliated Hospital of Hunan Normal University, 61 Jiefang West Rd, Changsha, Hunan 410000, P.R. China. Email: liyuanwei@hunnu.edu.cn

Table S1

The high BMI-attributable CKD ASMR for both sexes and all age groups in 204 GBD countries from 2021.

location	val	upper	lower	SDI value	SDI. Quintile
Ukraine	0.560593	0.958478	0.274741	0.760774	High-middle SDI
Tajikistan	0.580637	0.980724	0.258393	0.541511	Low-middle SDI
Belarus	0.606197	0.952679	0.304696	0.784485	High-middle SDI
Moldova	1.108598	1.73445	0.594822	0.732215	High-middle SDI
Bangladesh	1.338365	2.362714	0.645804	0.492421	Low-middle SDI
San Marino	1.349863	2.271149	0.611976	0.888005	High SDI
Finland	1.42715	2.225391	0.675839	0.859831	High SDI
United Kingdom	1.54774	2.471581	0.682305	0.859	High SDI
Lithuania	1.725571	2.659366	0.922262	0.856484	High SDI
Papua New Guinea	1.770616	3.639806	0.702134	0.417797	Low SDI
Cambodia	1.849301	3.340651	0.744717	0.473621	Low-middle SDI
Timor-Leste	1.898735	3.844228	0.785677	0.444668	Low SDI
Russian Federation	1.903347	2.7854	1.020563	0.808536	High-middle SDI
Japan	1.904862	3.086951	0.924922	0.871242	High SDI
Norway	1.9289	3.001033	0.987487	0.916133	High SDI
Iceland	2.006363	3.018791	0.995868	0.876362	High SDI
Viet Nam	2.175556	3.989999	0.862291	0.627934	Middle SDI
India	2.176874	3.616504	1.08772	0.575402	Low-middle SDI
Nepal	2.199373	4.011991	1.067105	0.433175	Low SDI
Poland	2.213704	3.454337	1.210434	0.812043	High SDI
Singapore	2.219963	3.683009	1.011686	0.856098	High SDI
South Korea	2.267088	3.833761	1.051947	0.886675	High SDI
Slovenia	2.349761	3.660425	1.250949	0.842431	High SDI
France	2.391203	3.521614	1.18101	0.838365	High SDI
Latvia	2.395442	3.724911	1.221776	0.830664	High SDI
Czechia	2.471583	3.822069	1.304318	0.82845	High SDI
Switzerland	2.486239	4.295034	1.056204	0.933059	High SDI
Sweden	2.509769	4.021869	1.246464	0.88688	High SDI
Kyrgyzstan	2.5209	3.95598	1.284942	0.603979	Low-middle SDI
Ireland	2.700791	4.096372	1.379868	0.873754	High SDI
Eritrea	2.725677	5.42799	1.118731	0.403864	Low SDI
Myanmar	2.821437	4.926606	1.281856	0.533901	Low-middle SDI
China	2.830031	4.731409	1.404543	0.72163	High-middle SDI
Burundi	2.835215	5.239354	1.283839	0.289374	Low SDI
Armenia	2.854988	4.542235	1.407656	0.701833	Middle SDI
Andorra	2.890208	4.946135	1.35679	0.869444	High SDI
Belgium	2.935588	4.412747	1.444282	0.853654	High SDI
Georgia	2.936017	4.926683	1.333593	0.732474	High-middle SDI
Azerbaijan	2.944169	4.999325	1.475975	0.694851	Middle SDI

Italy	2.958801	4.551266	1.444215	0.805774	High-middle SDI
Indonesia	2.999532	4.962372	1.371927	0.656868	Middle SDI
North Korea	3.016708	5.500094	1.360093	0.569855	Low-middle SDI
Romania	3.201326	4.703671	1.840561	0.768454	High-middle SDI
Kazakhstan	3.233364	5.377651	1.552966	0.725144	High-middle SDI
Bosnia and Herzegovina	3.312759	5.463769	1.618636	0.723078	High-middle SDI
Niger	3.32074	5.941245	1.472132	0.168073	Low SDI
Madagascar	3.337073	5.974737	1.354063	0.400247	Low SDI
Netherlands	3.356716	5.174874	1.699676	0.888464	High SDI
Hungary	3.390031	5.346683	1.678819	0.790755	High-middle SDI
Spain	3.401843	5.424448	1.586968	0.769284	High-middle SDI
Slovakia	3.430747	5.087339	1.914262	0.810611	High SDI
Mongolia	3.461353	5.82482	1.847283	0.617622	Low-middle SDI
Canada	3.465925	5.209219	1.687105	0.873171	High SDI
Monaco	3.50743	5.23127	1.81397	0.908263	High SDI
Malta	3.514142	5.732501	1.623871	0.801585	High-middle SDI
Sri Lanka	3.526793	6.828882	1.404111	0.701535	Middle SDI
Denmark	3.54572	5.414001	1.771628	0.896424	High SDI
Australia	3.564206	5.310355	1.90414	0.844253	High SDI
Uzbekistan	3.610198	6.082926	1.758778	0.662622	Middle SDI
Luxembourg	3.751584	6.165976	1.687313	0.884429	High SDI
Albania	3.825662	6.109608	2.024159	0.70685	Middle SDI
Rwanda	3.843892	7.340932	1.563218	0.435589	Low SDI
Mozambique	3.940526	7.39541	1.693352	0.326463	Low SDI
Djibouti	3.954079	7.50277	1.719533	0.487958	Low-middle SDI
Bhutan	3.976757	7.123991	1.836311	0.473062	Low-middle SDI
New Zealand	3.988194	5.724807	2.21664	0.849442	High SDI
Burkina Faso	4.003987	7.10825	1.745423	0.285118	Low SDI
Ethiopia	4.068788	7.400389	1.763678	0.358823	Low SDI
Solomon Islands	4.235694	8.847914	1.495308	0.42936	Low SDI
Uganda	4.394601	8.297476	1.978187	0.423261	Low SDI
Germany	4.405506	7.263537	1.947064	0.902957	High SDI
Chad	4.429422	7.863172	2.035806	0.240436	Low SDI
Laos	4.52418	8.503054	1.757543	0.489136	Low-middle SDI
North Macedonia	4.540353	7.43679	2.26031	0.75063	High-middle SDI
South Sudan	4.547141	8.668989	1.874983	0.278371	Low SDI
Maldives	4.555608	8.110422	1.989132	0.650887	Middle SDI
Portugal	4.587417	7.201806	2.259868	0.744152	High-middle SDI
Greenland	4.745731	7.85498	2.070884	0.82621	High SDI
Austria	4.75696	7.022533	2.501066	0.853837	High SDI
Turkmenistan	4.759905	8.179436	2.36436	0.682161	Middle SDI
Somalia	4.889569	9.301275	2.067323	0.077688	Low SDI
Croatia	4.896944	7.446746	2.518248	0.798341	High-middle SDI

Colombia	4.910875	7.738676	2.521401	0.655443	Middle SDI
Sierra Leone	4.974729	8.572525	2.390339	0.358666	Low SDI
Haiti	5.233014	11.63698	2.044066	0.448278	Low SDI
Yemen	5.312291	9.797076	2.521656	0.450376	Low SDI
Uruguay	5.367083	8.339747	2.62386	0.719283	High-middle SDI
Pakistan	5.472371	9.107954	2.623929	0.504029	Low-middle SDI
Angola	5.557825	10.06192	2.436437	0.453722	Low SDI
Estonia	5.593443	8.219267	2.929768	0.844918	High SDI
Taiwan (Province of China)	5.60707	9.762514	2.567491	0.874747	High SDI
Guinea	5.675837	10.08611	2.52469	0.336401	Low SDI
Thailand	5.707689	10.1292	2.323652	0.682548	Middle SDI
Serbia	5.729662	8.906111	3.062321	0.792416	High-middle SDI
Cyprus	5.956817	9.98283	2.828876	0.835631	High SDI
Israel	5.958423	9.393167	2.783874	0.809012	High-middle SDI
Mali	5.977531	10.31359	2.898302	0.26858	Low SDI
Bulgaria	6.002033	9.165809	3.219816	0.768151	High-middle SDI
Philippines	6.029939	10.55641	2.510736	0.651219	Middle SDI
Cuba	6.081782	9.135836	3.442494	0.66873	Middle SDI
Brunei Darussalam	6.224597	10.36934	3.075742	0.810234	High-middle SDI
Kenya	6.616376	11.12065	3.127524	0.523768	Low-middle SDI
Comoros	6.644512	12.35413	3.027789	0.475979	Low-middle SDI
Malawi	6.69304	11.8049	3.084804	0.384554	Low SDI
Cabo Verde	6.849904	12.01612	3.004261	0.533535	Low-middle SDI
Benin	7.041405	11.78347	3.190117	0.373487	Low SDI
Central African Republic	7.119977	13.11935	3.348696	0.309168	Low SDI
Honduras	7.175985	11.88765	3.404527	0.513037	Low-middle SDI
Greece	7.223976	10.66606	3.704606	0.791854	High-middle SDI
Iran	7.234706	11.04095	3.671879	0.697207	Middle SDI
Chile	7.400745	11.14143	3.891121	0.771515	High-middle SDI
United Republic of Tanzania	7.454221	12.47119	3.794883	0.446568	Low SDI
Nigeria	7.531303	12.05329	3.717191	0.503391	Low-middle SDI
Togo	7.621711	13.22826	3.63576	0.408534	Low SDI
Montenegro	7.676762	11.62755	3.933926	0.795801	High-middle SDI
Botswana	7.684471	12.8202	3.620034	0.642722	Middle SDI
Guinea-Bissau	7.745106	13.7394	3.435687	0.35311	Low SDI
Malaysia	7.912236	12.18862	4.113922	0.742524	High-middle SDI
Namibia	7.985501	13.08246	4.062675	0.617565	Low-middle SDI
Kuwait	7.998021	11.67485	4.388802	0.846651	High SDI
Brazil	8.06824	11.20095	4.547944	0.653044	Middle SDI
Guam	8.14738	12.87216	4.07957	0.803982	High-middle SDI
Vanuatu	8.168985	15.57923	3.419672	0.473101	Low-middle SDI

Bermuda	8.249856	11.57112	4.831262	0.821365	High SDI
Tonga	8.426314	13.94529	3.516856	0.62635	Middle SDI
Côte d'Ivoire	8.541995	14.10583	4.03821	0.425942	Low SDI
Gambia	8.697052	15.48742	4.2176	0.409714	Low SDI
Zambia	8.773216	15.81663	4.059621	0.505949	Low-middle SDI
Cook Islands	8.813951	14.12411	4.277775	0.77911	High-middle SDI
Zimbabwe	8.872223	15.44751	4.041298	0.473819	Low-middle SDI
Senegal	8.881034	14.85158	4.172861	0.408054	Low SDI
United States Virgin Islands	9.439762	14.49455	4.928818	0.821831	High SDI
Dominican Republic	9.483748	14.63334	4.885858	0.619388	Middle SDI
Democratic Republic of the Congo	9.732396	17.05495	4.650567	0.38318	Low SDI
Costa Rica	9.901748	15.24021	5.217097	0.70034	Middle SDI
United States of America	9.982018	14.14787	5.472171	0.862448	High SDI
Tunisia	10.12029	17.52203	4.909249	0.682432	Middle SDI
Argentina	10.26267	14.8334	5.774493	0.723123	High-middle SDI
Congo	10.29107	17.91926	4.227437	0.583075	Low-middle SDI
Sudan	10.49519	17.55625	4.920379	0.54195	Low-middle SDI
Jamaica	10.605	16.63122	5.329629	0.683263	Middle SDI
Paraguay	10.614	16.48606	5.300946	0.635718	Middle SDI
Tuvalu	11.10443	22.81789	4.136352	0.576621	Low-middle SDI
South Africa	11.37619	17.63587	5.836033	0.679627	Middle SDI
Ghana	11.43301	18.55387	5.634468	0.56493	Low-middle SDI
Panama	11.78559	17.55582	6.282642	0.708865	Middle SDI
Tokelau	11.9065	20.70755	5.173725	0.686426	Middle SDI
Lesotho	11.933	20.85632	5.130296	0.510393	Low-middle SDI
Peru	11.93734	18.67552	6.093335	0.662054	Middle SDI
Turkey	12.11104	18.47996	6.539256	0.712693	High-middle SDI
Liberia	12.33638	21.59348	5.889458	0.352442	Low SDI
Saint Vincent and the Grenadines	12.51804	19.94599	6.46393	0.637196	Middle SDI
Suriname	12.63203	21.30312	6.212173	0.633666	Middle SDI
Morocco	12.64222	20.62146	6.474299	0.562698	Low-middle SDI
Lebanon	13.09518	20.28779	6.907501	0.744746	High-middle SDI
Barbados	13.29123	20.2638	7.491175	0.746749	High-middle SDI
Algeria	13.29275	20.682	7.129027	0.659501	Middle SDI
Mauritania	13.58443	22.66891	6.63157	0.498945	Low-middle SDI
Saint Lucia	13.66294	21.52584	7.091125	0.67251	Middle SDI
Afghanistan	13.9367	27.22518	5.768963	0.3372	Low SDI
Kiribati	14.11765	27.19779	5.65902	0.527187	Low-middle SDI
Venezuela	14.4996	23.06411	7.489627	0.596513	Low-middle SDI
Seychelles	14.62815	23.82925	6.898582	0.730151	High-middle SDI

Puerto Rico	14.6606	21.85729	8.668445	0.825526	High SDI
Marshall Islands	15.29808	43.5007	3.45294	0.574091	Low-middle SDI
Jordan	15.54054	22.55983	8.68193	0.725307	High-middle SDI
Guatemala	15.59738	24.85296	7.956102	0.539972	Low-middle SDI
Bahamas	15.78641	23.31193	8.59029	0.805021	High-middle SDI
Trinidad and Tobago	15.81788	25.07312	8.333912	0.768763	High-middle SDI
Equatorial Guinea	16.46722	29.43339	7.065562	0.657857	Middle SDI
Samoa	16.55699	26.91446	7.460737	0.593393	Low-middle SDI
Palestine	16.99081	26.08435	8.695079	0.631012	Middle SDI
Oman	17.42834	26.44452	9.751323	0.773392	High-middle SDI
Iraq	17.56052	28.7403	8.406277	0.662626	Middle SDI
Antigua and Barbuda	17.76761	27.27853	9.937593	0.749887	High-middle SDI
Nicaragua	17.89868	29.02194	8.955651	0.523958	Low-middle SDI
United Arab Emirates	17.94136	26.52298	8.317274	0.849318	High SDI
Micronesia	18.00029	32.952	7.094283	0.587535	Low-middle SDI
Cameroon	18.11776	30.70096	8.990298	0.479691	Low-middle SDI
Ecuador	18.39277	28.34594	10.02393	0.661017	Middle SDI
Palau	19.00458	32.32848	8.600254	0.754047	High-middle SDI
Libya	19.00503	30.93026	8.850234	0.725771	High-middle SDI
Fiji	19.11045	30.38036	8.89895	0.675052	Middle SDI
Belize	19.45233	28.45857	11.00423	0.610229	Low-middle SDI
Bolivia	19.46243	30.71812	9.835219	0.599011	Low-middle SDI
Eswatini	19.60304	31.44744	9.914155	0.58546	Low-middle SDI
Sao Tome and Principe	19.66156	31.43939	9.161582	0.505414	Low-middle SDI
Grenada	19.80208	31.46703	10.88083	0.668993	Middle SDI
Qatar	19.81263	28.62011	11.5955	0.846861	High SDI
Mexico	19.85181	28.91274	11.27373	0.664575	Middle SDI
Syrian Arab Republic	20.37276	31.80086	10.2878	0.623004	Middle SDI
Niue	20.51202	37.97542	7.765277	0.726222	High-middle SDI
Mauritius	20.91475	33.26493	10.29125	0.71826	High-middle SDI
Guyana	21.10235	33.23897	11.52972	0.650812	Middle SDI
Nauru	22.04903	39.94273	9.541495	0.625178	Middle SDI
Northern Mariana Islands	22.13419	36.87358	9.616592	0.771535	High-middle SDI
Bahrain	22.87045	34.29708	13.17258	0.753043	High-middle SDI
Saint Kitts and Nevis	23.06816	35.01606	12.55937	0.754987	High-middle SDI
Gabon	23.25886	39.48915	9.278996	0.634691	Middle SDI
Dominica	23.98027	35.31469	13.54059	0.746967	High-middle SDI
El Salvador	24.51763	38.0937	12.94246	0.563775	Low-middle SDI
American Samoa	29.53219	48.50456	13.17667	0.723728	High-middle SDI
Egypt	30.74307	45.35674	16.22909	0.606787	Low-middle SDI
Saudi Arabia	35.61654	53.05655	19.8014	0.815143	High SDI

Table S2

The high BMI-attributable CKD ASDR for both sexes and all age groups in 204 GBD countries from 2021.

location	val	upper	lower	SDI value	SDI. Quintile
Finland	39.77618	61.1236	19.59426	0.859831	High SDI
South Korea	41.45605	69.69956	19.82206	0.886675	High SDI
Belarus	42.01222	63.25934	23.07014	0.784485	High-middle SDI
Bangladesh	42.0967	72.26135	20.31136	0.492421	Low-middle SDI
Japan	44.93476	71.38166	22.21422	0.871242	High SDI
Ukraine	46.23494	71.1355	25.11155	0.760774	High-middle SDI
Norway	46.9725	70.46142	24.42404	0.916133	High SDI
San Marino	47.45616	71.97723	25.36279	0.888005	High SDI
Cambodia	47.64838	87.09523	19.33516	0.473621	Low-middle SDI
Viet Nam	49.53851	91.31562	19.56666	0.627934	Middle SDI
Iceland	49.57841	72.26381	27.2369	0.876362	High SDI
Timor-Leste	49.66552	100.3351	20.93284	0.444668	Low SDI
Singapore	49.81669	78.76471	23.81197	0.856098	High SDI
Sweden	50.75596	78.55764	25.83344	0.88688	High SDI
Switzerland	50.77646	83.48516	23.11787	0.933059	High SDI
France	50.94577	74.31857	26.23574	0.838365	High SDI
United Kingdom	51.34061	79.66344	25.55738	0.859	High SDI
Papua New Guinea	57.67179	110.8409	23.43062	0.417797	Low SDI
Italy	58.16529	87.4575	28.75956	0.805774	High-middle SDI
Russian Federation	59.78719	87.43623	33.3267	0.808536	High-middle SDI
Tajikistan	60.83739	94.06555	33.01095	0.541511	Low-middle SDI
Eritrea	62.77067	124.1781	25.8981	0.403864	Low SDI
Burundi	62.79935	116.8568	28.55321	0.289374	Low SDI
Slovenia	64.15518	94.15431	35.82223	0.842431	High SDI
Poland	65.09634	99.40248	36.8903	0.812043	High SDI
China	66.96834	109.5398	33.81533	0.72163	High-middle SDI
Netherlands	67.33234	100.1546	33.80095	0.888464	High SDI
Andorra	67.42239	106.4944	35.33683	0.869444	High SDI
Lithuania	67.48942	100.0233	35.44215	0.856484	High SDI
India	67.59517	108.5392	33.90968	0.575402	Low-middle SDI
Czechia	67.70248	101.9219	37.3305	0.82845	High SDI
Spain	68.56306	104.0082	34.56346	0.769284	High-middle SDI
North Korea	69.848	128.1963	30.65518	0.569855	Low-middle SDI
Belgium	69.90825	102.2324	36.1519	0.853654	High SDI
Canada	70.55023	100.3768	35.91018	0.873171	High SDI
Australia	73.26831	102.9596	41.61183	0.844253	High SDI
Denmark	73.39817	109.8046	38.53421	0.896424	High SDI
Madagascar	73.72861	131.9093	30.80612	0.400247	Low SDI
Ireland	75.50266	111.6905	41.33378	0.873754	High SDI

Moldova	77.42683	115.6115	45.77055	0.732215	High-middle SDI
Nepal	77.78164	132.4122	36.05389	0.433175	Low SDI
Luxembourg	78.75508	121.7686	38.91075	0.884429	High SDI
Myanmar	79.23782	139.1548	36.62212	0.533901	Low-middle SDI
Monaco	79.97975	116.6113	44.1842	0.908263	High SDI
Latvia	80.58455	122.4571	43.87024	0.830664	High SDI
Malta	80.73546	124.6508	38.22354	0.801585	High-middle SDI
Germany	81.21177	127.1633	38.95296	0.902957	High SDI
Hungary	82.51494	126.5831	42.9224	0.790755	High-middle SDI
Rwanda	83.3001	156.243	33.83407	0.435589	Low SDI
Niger	84.4976	147.7707	39.08377	0.168073	Low SDI
Djibouti	86.77631	162.948	38.45949	0.487958	Low-middle SDI
Austria	86.80314	125.7333	46.0843	0.853837	High SDI
Portugal	87.15963	132.6352	46.0499	0.744152	High-middle SDI
Ethiopia	88.06927	156.6578	39.36413	0.358823	Low SDI
Indonesia	88.13655	146.1392	40.45304	0.656868	Middle SDI
Bosnia and Herzegovina	88.2279	138.3978	43.93327	0.723078	High-middle SDI
Sri Lanka	90.41338	170.6746	37.61993	0.701535	Middle SDI
New Zealand	90.51011	127.4585	52.5034	0.849442	High SDI
Slovakia	93.26792	133.8128	53.50718	0.810611	High SDI
Burkina Faso	94.72215	167.6374	40.68012	0.285118	Low SDI
Albania	95.01121	142.542	53.10949	0.70685	Middle SDI
Mozambique	95.33251	177.0731	40.8462	0.326463	Low SDI
Cyprus	100.4594	164.6985	48.57999	0.835631	High SDI
Greenland	100.901	162.09	46.816	0.82621	High SDI
Romania	102	148.1134	60.53156	0.768454	High-middle SDI
Uganda	102.6125	188.3196	45.9419	0.423261	Low SDI
South Sudan	102.9986	199.8188	42.69903	0.278371	Low SDI
Armenia	107.2613	162.3774	56.72514	0.701833	Middle SDI
Chad	107.4401	190.4049	50.50701	0.240436	Low SDI
Croatia	107.473	161.4144	56.64723	0.798341	High-middle SDI
Uruguay	108.2719	161.3731	54.65343	0.719283	High-middle SDI
Kyrgyzstan	111.8861	169.6513	61.66288	0.603979	Low-middle SDI
Bhutan	113.0432	192.9316	52.25655	0.473062	Low-middle SDI
Israel	114.3831	175.5694	56.31901	0.809012	High-middle SDI
Laos	115.1509	218.4612	45.57388	0.489136	Low-middle SDI
Georgia	115.2957	185.7711	57.62005	0.732474	High-middle SDI
North Macedonia	119.3432	183.9813	60.85082	0.75063	High-middle SDI
Taiwan (Province of China)	119.5739	200.6232	54.57203	0.874747	High SDI
Maldives	119.7796	203.9478	54.89878	0.650887	Middle SDI
Somalia	120.8537	226.2384	53.01307	0.077688	Low SDI
Kazakhstan	120.8699	185.1477	61.62915	0.725144	High-middle SDI

Sierra Leone	121.196	204.7298	59.71681	0.358666	Low SDI
Yemen	124.1638	219.3095	60.2703	0.450376	Low SDI
Colombia	124.7265	186.931	63.95131	0.655443	Middle SDI
Solomon Islands	125.0175	244.1312	45.26446	0.42936	Low SDI
Azerbaijan	125.4003	197.5755	68.67466	0.694851	Middle SDI
Serbia	126.4383	193.7492	67.24005	0.792416	High-middle SDI
Mongolia	131.6377	203.1329	71.64144	0.617622	Low-middle SDI
Estonia	133.9683	193.923	71.79089	0.844918	High SDI
Greece	136.7064	199.1979	72.69197	0.791854	High-middle SDI
Angola	138.3575	246.0777	60.69238	0.453722	Low SDI
Guinea	143.1051	248.6309	65.11074	0.336401	Low SDI
Thailand	143.1249	251.5829	61.95743	0.682548	Middle SDI
Brunei Darussalam	144.516	227.2258	72.51586	0.810234	High-middle SDI
Chile	145.3814	211.6899	80.1012	0.771515	High-middle SDI
Comoros	146.396	264.8565	64.44156	0.475979	Low-middle SDI
Uzbekistan	146.3983	228.2146	74.22201	0.662622	Middle SDI
Kenya	147.673	240.6634	71.76145	0.523768	Low-middle SDI
Mali	148.012	252.5617	76.3846	0.26858	Low SDI
Haiti	148.9526	315.0899	61.61428	0.448278	Low SDI
Malawi	149.544	256.1771	71.45574	0.384554	Low SDI
Pakistan	154.4892	260.3402	72.48933	0.504029	Low-middle SDI
Iran	154.9327	230.6573	80.86556	0.697207	Middle SDI
Cuba	155.6551	221.5011	91.50464	0.66873	Middle SDI
Philippines	158.8559	274.3812	66.98881	0.651219	Middle SDI
Bulgaria	159.734	242.8912	86.52062	0.768151	High-middle SDI
Cabo Verde	159.8788	271.1761	76.16847	0.533535	Low-middle SDI
Kuwait	161.2866	228.3211	95.13363	0.846651	High SDI
Montenegro	164.7397	245.2893	88.40615	0.795801	High-middle SDI
Togo	169.1462	281.2468	82.37203	0.408534	Low SDI
United Republic of Tanzania	170.0517	285.335	88.62107	0.446568	Low SDI
Benin	170.5235	282.5201	78.86086	0.373487	Low SDI
Nigeria	174.5309	280.0069	87.23255	0.503391	Low-middle SDI
Botswana	180.1347	294.2109	85.65708	0.642722	Middle SDI
Turkmenistan	182.404	297.5772	96.88207	0.682161	Middle SDI
Bermuda	184.5631	250.4865	115.2716	0.821365	High SDI
Honduras	190.671	308.3361	92.39	0.513037	Low-middle SDI
Central African Republic	191.894	337.1901	90.39266	0.309168	Low SDI
Namibia	192.4109	309.3214	97.03548	0.617565	Low-middle SDI
Guinea-Bissau	192.7458	345.5862	88.03128	0.35311	Low SDI
Brazil	196.5765	268.0301	113.1775	0.653044	Middle SDI
Senegal	198.5141	333.4547	95.51134	0.408054	Low SDI
Malaysia	202.4669	314.1005	104.6234	0.742524	High-middle SDI

Côte d'Ivoire	207.1494	351.7629	102.0111	0.425942	Low SDI
Argentina	208.1274	296.0946	117.7335	0.723123	High-middle SDI
Tunisia	208.9615	338.118	104.0819	0.682432	Middle SDI
Zambia	210.318	373.5568	100.5386	0.505949	Low-middle SDI
Gambia	211.0411	358.3426	104.2056	0.409714	Low SDI
Cook Islands	217.9446	330.3386	108.5488	0.77911	High-middle SDI
Vanuatu	218.2691	401.5163	96.25511	0.473101	Low-middle SDI
Zimbabwe	224.6765	381.4618	103.5669	0.473819	Low-middle SDI
Tonga	224.7202	343.5129	100.2519	0.62635	Middle SDI
United States of America	226.7614	307.1362	134.1178	0.862448	High SDI
Democratic Republic of the Congo	230.8774	394.0662	110.991	0.38318	Low SDI
Turkey	239.1329	356.8924	134.1397	0.712693	High-middle SDI
Ghana	242.438	398.038	125.4655	0.56493	Low-middle SDI
Guam	243.9218	372.9628	126.4799	0.803982	High-middle SDI
Sudan	244.4995	407.5834	116.8441	0.54195	Low-middle SDI
United States Virgin Islands	245.1818	357.244	142.1293	0.821831	High SDI
Algeria	245.262	367.6698	133.8641	0.659501	Middle SDI
Lebanon	248.4956	380.0717	137.6377	0.744746	High-middle SDI
Paraguay	249.9665	369.6586	135.1717	0.635718	Middle SDI
Dominican Republic	251.1912	369.5373	134.1463	0.619388	Middle SDI
Costa Rica	253.0422	375.1605	139.154	0.70034	Middle SDI
Peru	256.8874	384.3463	142.4497	0.662054	Middle SDI
Congo	259.3059	444.1668	113.6541	0.583075	Low-middle SDI
Morocco	274.4912	439.8279	144.9173	0.562698	Low-middle SDI
Mauritania	275.8977	446.6207	135.9192	0.498945	Low-middle SDI
South Africa	276.1534	415.815	147.0901	0.679627	Middle SDI
Jamaica	277.743	420.7975	146.6286	0.683263	Middle SDI
Panama	280.2655	412.7461	152.0258	0.708865	Middle SDI
Tuvalu	282.4288	518.3974	108.1057	0.576621	Low-middle SDI
Tokelau	288.1694	474.9849	129.3033	0.686426	Middle SDI
Lesotho	291.2497	498.3876	130.8594	0.510393	Low-middle SDI
Jordan	300.6357	429.7637	178.4615	0.725307	High-middle SDI
Barbados	304.9534	449.2988	176.3948	0.746749	High-middle SDI
Liberia	306.6957	514.6552	151.2537	0.352442	Low SDI
Saint Vincent and the Grenadines	314.7389	483.7499	169.647	0.637196	Middle SDI
Palestine	328.5427	496.5238	176.6476	0.631012	Middle SDI
Suriname	340.8802	548.9923	174.7863	0.633666	Middle SDI
Saint Lucia	342.3019	513.7264	185.1304	0.67251	Middle SDI
Puerto Rico	343.3556	487.6147	209.2957	0.825526	High SDI
Afghanistan	349.1983	672.0329	147.4203	0.3372	Low SDI

Seychelles	352.8328	553.447	173.918	0.730151	High-middle SDI
Kiribati	354.1206	645.5232	154.0575	0.527187	Low-middle SDI
United Arab Emirates	356.8757	506.2816	184.2722	0.849318	High SDI
Venezuela	356.8955	548.2586	192.5312	0.596513	Low-middle SDI
Qatar	357.487	516.5842	216.8382	0.846861	High SDI
Oman	362.0512	540.4518	209.9627	0.773392	High-middle SDI
Iraq	362.3144	589.4267	179.2138	0.662626	Middle SDI
Equatorial Guinea	375.1619	655.3749	167.4518	0.657857	Middle SDI
Marshall Islands	391.8006	1007.255	99.74255	0.574091	Low-middle SDI
Ecuador	395.5467	629.7377	219.9106	0.661017	Middle SDI
Antigua and Barbuda	399.6693	585.8732	228.9732	0.749887	High-middle SDI
Bahamas	404.7597	573.7891	233.5199	0.805021	High-middle SDI
Guatemala	405.2082	636.4768	211.088	0.539972	Low-middle SDI
Samoa	406.7936	625.2415	193.5333	0.593393	Low-middle SDI
Syrian Arab Republic	409.1396	633.8108	221.4564	0.623004	Middle SDI
Trinidad and Tobago	409.8158	640.435	221.0645	0.768763	High-middle SDI
Bahrain	410.0081	604.2061	243.3921	0.753043	High-middle SDI
Libya	414.1314	659.5606	198.8246	0.725771	High-middle SDI
Cameroon	414.3533	690.3902	210.7133	0.479691	Low-middle SDI
Bolivia	424.2844	660.3046	229.3994	0.599011	Low-middle SDI
Sao Tome and Principe	425.18	659.7199	210.6235	0.505414	Low-middle SDI
Fiji	427.1207	667.4059	200.3126	0.675052	Middle SDI
Palau	427.1402	687.9495	204.4001	0.754047	High-middle SDI
Micronesia	456.8563	770.6575	191.8069	0.587535	Low-middle SDI
Eswatini	462.9189	753.8558	227.1389	0.58546	Low-middle SDI
Niue	469.7786	844.9539	195.4279	0.726222	High-middle SDI
Belize	476.8226	657.7069	287.1952	0.610229	Low-middle SDI
Nicaragua	484.1135	774.3557	252.3046	0.523958	Low-middle SDI
Northern Mariana Islands	490.4419	772.0543	217.8773	0.771535	High-middle SDI
Grenada	493.7689	749.0775	282.0336	0.668993	Middle SDI
Gabon	513.5757	838.9609	229.0866	0.634691	Middle SDI
Mexico	517.3615	743.7511	301.2783	0.664575	Middle SDI
Saint Kitts and Nevis	517.7125	751.2198	289.9128	0.754987	High-middle SDI
Mauritius	518.9281	830.5009	257.8673	0.71826	High-middle SDI
Guyana	549.6499	855.5194	306.5082	0.650812	Middle SDI
Dominica	550.8314	794.7116	316.242	0.746967	High-middle SDI
Nauru	559.7349	900.8456	260.0376	0.625178	Middle SDI
Egypt	611.7648	896.3627	335.9143	0.606787	Low-middle SDI
El Salvador	622.2287	948.5533	343.5521	0.563775	Low-middle SDI
American Samoa	668.9003	1029.317	321.3892	0.723728	High-middle SDI
Saudi Arabia	764.2562	1126.37	444.9366	0.815143	High SDI

Table S3

The high BMI-attributable CKD death EAPC for both sexes and all age groups in 204 GBD countries from 1990 to 2021.

location	EAPC	LCI	UCI	Pr	SDIvalue	SDI.Quintile
Poland	-1.89	-2.5	-1.27	0	0.812043	High SDI
Cyprus	-0.81	-1.07	-0.55	0	0.835631	High SDI
Maldives	-0.79	-1.01	-0.57	0	0.650887	Middle SDI
Kuwait	-0.5	-0.75	-0.24	0.001	0.846651	High SDI
Ethiopia	-0.43	-0.6	-0.26	0	0.358823	Low SDI
Czechia	-0.29	-0.49	-0.08	0.011	0.82845	High SDI
Japan	-0.26	-0.38	-0.14	0	0.871242	High SDI
South Korea	-0.18	-0.36	0.01	0.068	0.886675	High SDI
Andorra	-0.11	-0.34	0.12	0.347	0.869444	High SDI
Slovakia	-0.04	-0.1	0.01	0.133	0.810611	High SDI
Spain	-0.04	-0.14	0.06	0.442	0.769284	High-middle SDI
Rwanda	0	-0.37	0.38	0.987	0.435589	Low SDI
Mongolia	0.02	-0.13	0.18	0.759	0.617622	Low-middle SDI
Greece	0.09	-0.89	1.08	0.859	0.791854	High-middle SDI
Solomon Islands	0.16	-0.04	0.36	0.124	0.42936	Low SDI
Niger	0.26	0.15	0.37	0	0.168073	Low SDI
Burundi	0.32	0.14	0.5	0.001	0.289374	Low SDI
Israel	0.36	-0.14	0.86	0.164	0.809012	High-middle SDI
Iraq	0.44	0.23	0.64	0	0.662626	Middle SDI
Taiwan (Province of China)	0.58	0.26	0.9	0.001	0.874747	High SDI
Argentina	0.59	0.23	0.96	0.003	0.723123	High-middle SDI
San Marino	0.62	0.18	1.05	0.009	0.888005	High SDI
Romania	0.63	0.15	1.12	0.015	0.768454	High-middle SDI
Serbia	0.63	0.5	0.76	0	0.792416	High-middle SDI
Myanmar	0.64	0.57	0.72	0	0.533901	Low-middle SDI
Papua New Guinea	0.64	0.57	0.72	0	0.417797	Low SDI
Albania	0.68	0.42	0.93	0	0.70685	Middle SDI
Mali	0.68	0.52	0.83	0	0.26858	Low SDI
Palestine	0.7	0.46	0.93	0	0.631012	Middle SDI
Mauritania	0.75	0.58	0.93	0	0.498945	Low-middle SDI
Cameroon	0.79	0.55	1.04	0	0.479691	Low-middle SDI
Colombia	0.8	0.63	0.97	0	0.655443	Middle SDI
Italy	0.81	0.67	0.95	0	0.805774	High-middle SDI
Senegal	0.83	0.71	0.96	0	0.408054	Low SDI
Malta	0.86	0.54	1.19	0	0.801585	High-middle SDI
Jordan	0.88	0.56	1.2	0	0.725307	High-middle SDI
Sierra Leone	0.88	0.84	0.91	0	0.358666	Low SDI
Guinea-Bissau	0.9	0.83	0.96	0	0.35311	Low SDI

United States Virgin Islands	0.94	0.66	1.21	0	0.821831	High SDI
Greenland	0.95	0.76	1.15	0	0.82621	High SDI
Portugal	0.96	0.46	1.45	0.001	0.744152	High-middle SDI
Chad	1.01	0.89	1.14	0	0.240436	Low SDI
Qatar	1.01	0.35	1.68	0.005	0.846861	High SDI
Bosnia and Herzegovina	1.02	0.81	1.22	0	0.723078	High-middle SDI
Bermuda	1.06	0.75	1.37	0	0.821365	High SDI
Guam	1.1	0.7	1.49	0	0.803982	High-middle SDI
Cambodia	1.12	0.87	1.37	0	0.473621	Low-middle SDI
Peru	1.13	0.92	1.34	0	0.662054	Middle SDI
Sri Lanka	1.19	1	1.38	0	0.701535	Middle SDI
Congo	1.21	1.07	1.35	0	0.583075	Low-middle SDI
Bhutan	1.22	1.17	1.27	0	0.473062	Low-middle SDI
Côte d'Ivoire	1.22	1.11	1.34	0	0.425942	Low SDI
Laos	1.22	1.19	1.24	0	0.489136	Low-middle SDI
France	1.24	1	1.48	0	0.838365	High SDI
Turkey	1.24	0.78	1.71	0	0.712693	High-middle SDI
Brazil	1.26	1.06	1.45	0	0.653044	Middle SDI
Luxembourg	1.29	1.01	1.58	0	0.884429	High SDI
Guinea	1.3	1.24	1.36	0	0.336401	Low SDI
Burkina Faso	1.31	1.23	1.39	0	0.285118	Low SDI
Tajikistan	1.34	0.92	1.77	0	0.541511	Low-middle SDI
Lebanon	1.37	1.2	1.55	0	0.744746	High-middle SDI
Afghanistan	1.38	1.19	1.58	0	0.3372	Low SDI
China	1.41	1.31	1.51	0	0.72163	High-middle SDI
Puerto Rico	1.41	0.99	1.83	0	0.825526	High SDI
Slovenia	1.41	1.1	1.71	0	0.842431	High SDI
Vanuatu	1.41	1.35	1.46	0	0.473101	Low-middle SDI
Benin	1.43	1.34	1.53	0	0.373487	Low SDI
North Korea	1.45	1.38	1.52	0	0.569855	Low-middle SDI
North Macedonia	1.46	1.11	1.82	0	0.75063	High-middle SDI
Cook Islands	1.47	1.37	1.57	0	0.77911	High-middle SDI
Ireland	1.48	1.27	1.69	0	0.873754	High SDI
Syrian Arab Republic	1.51	1.25	1.77	0	0.623004	Middle SDI
Uruguay	1.51	1.25	1.76	0	0.719283	High-middle SDI
Central African Republic	1.56	1.51	1.62	0	0.309168	Low SDI
Belgium	1.59	1.29	1.89	0	0.853654	High SDI
Democratic Republic of the Congo	1.59	1.44	1.75	0	0.38318	Low SDI

Chile	1.61	1.15	2.07	0	0.771515	High-middle SDI
Jamaica	1.61	0.97	2.25	0	0.683263	Middle SDI
Somalia	1.61	1.52	1.69	0	0.077688	Low SDI
Canada	1.62	1.38	1.86	0	0.873171	High SDI
Liberia	1.67	1.35	1.99	0	0.352442	Low SDI
Togo	1.68	1.61	1.75	0	0.408534	Low SDI
Gambia	1.69	1.56	1.81	0	0.409714	Low SDI
Angola	1.71	1.54	1.88	0	0.453722	Low SDI
Brunei Darussalam	1.71	1.49	1.93	0	0.810234	High-middle SDI
Nigeria	1.75	1.66	1.84	0	0.503391	Low-middle SDI
Saint Lucia	1.76	1.47	2.06	0	0.67251	Middle SDI
Timor-Leste	1.76	1.42	2.1	0	0.444668	Low SDI
Malaysia	1.78	1.58	1.99	0	0.742524	High-middle SDI
Yemen	1.79	1.56	2.03	0	0.450376	Low SDI
Uganda	1.81	1.64	1.98	0	0.423261	Low SDI
Croatia	1.85	1.53	2.18	0	0.798341	High-middle SDI
Montenegro	1.88	1.6	2.16	0	0.795801	High-middle SDI
Russian Federation	1.92	1.45	2.4	0	0.808536	High-middle SDI
Samoa	1.99	1.9	2.07	0	0.593393	Low-middle SDI
Kiribati	2	1.73	2.27	0	0.527187	Low-middle SDI
Malawi	2.02	1.81	2.23	0	0.384554	Low SDI
Bangladesh	2.03	1.85	2.21	0	0.492421	Low-middle SDI
Australia	2.04	1.76	2.32	0	0.844253	High SDI
Madagascar	2.04	1.91	2.17	0	0.400247	Low SDI
Singapore	2.04	1.46	2.62	0	0.856098	High SDI
United Republic of Tanzania	2.04	1.99	2.09	0	0.446568	Low SDI
Iran	2.05	1.91	2.18	0	0.697207	Middle SDI
Northern Mariana Islands	2.06	1.87	2.25	0	0.771535	High-middle SDI
Zambia	2.06	1.87	2.25	0	0.505949	Low-middle SDI
United Kingdom	2.07	1.75	2.39	0	0.859	High SDI
South Sudan	2.1	1.88	2.32	0	0.278371	Low SDI
Monaco	2.11	1.83	2.4	0	0.908263	High SDI
Bahrain	2.12	1.75	2.49	0	0.753043	High-middle SDI
Haiti	2.13	2	2.26	0	0.448278	Low SDI
Switzerland	2.15	1.8	2.5	0	0.933059	High SDI
New Zealand	2.16	1.71	2.6	0	0.849442	High SDI
Paraguay	2.18	2.05	2.31	0	0.635718	Middle SDI
Comoros	2.21	2.1	2.31	0	0.475979	Low-middle SDI
Nauru	2.21	2.08	2.33	0	0.625178	Middle SDI
Bolivia	2.23	2.14	2.33	0	0.599011	Low-middle SDI
Finland	2.24	2.02	2.47	0	0.859831	High SDI
Marshall Islands	2.24	2.01	2.47	0	0.574091	Low-middle SDI

Tonga	2.24	2.01	2.46	0	0.62635	Middle SDI
Micronesia	2.32	1.93	2.7	0	0.587535	Low-middle SDI
Tokelau	2.35	2.18	2.53	0	0.686426	Middle SDI
Namibia	2.42	2.07	2.77	0	0.617565	Low-middle SDI
Sao Tome and Principe	2.46	2.4	2.52	0	0.505414	Low-middle SDI
Dominica	2.47	2.38	2.56	0	0.746967	High-middle SDI
Iceland	2.5	2.31	2.7	0	0.876362	High SDI
Pakistan	2.5	2.24	2.76	0	0.504029	Low-middle SDI
Egypt	2.51	2.41	2.62	0	0.606787	Low-middle SDI
Fiji	2.54	2.12	2.96	0	0.675052	Middle SDI
Thailand	2.54	2.32	2.76	0	0.682548	Middle SDI
Suriname	2.55	2.35	2.75	0	0.633666	Middle SDI
Palau	2.57	2.36	2.78	0	0.754047	High-middle SDI
Netherlands	2.58	2.2	2.96	0	0.888464	High SDI
Sudan	2.58	2.37	2.79	0	0.54195	Low-middle SDI
Tuvalu	2.58	2.47	2.68	0	0.576621	Low-middle SDI
Barbados	2.6	2.28	2.92	0	0.746749	High-middle SDI
Kyrgyzstan	2.6	1.67	3.53	0	0.603979	Low-middle SDI
Djibouti	2.61	2.5	2.71	0	0.487958	Low-middle SDI
Eritrea	2.61	2.54	2.67	0	0.403864	Low SDI
Gabon	2.63	2.41	2.85	0	0.634691	Middle SDI
Morocco	2.7	2.58	2.83	0	0.562698	Low-middle SDI
Norway	2.72	2.38	3.06	0	0.916133	High SDI
Viet Nam	2.76	2.56	2.95	0	0.627934	Middle SDI
Costa Rica	2.77	2.33	3.22	0	0.70034	Middle SDI
Moldova	2.78	2.3	3.27	0	0.732215	High-middle SDI
Bahamas	2.79	2.55	3.03	0	0.805021	High-middle SDI
Indonesia	2.82	2.68	2.96	0	0.656868	Middle SDI
Mexico	2.84	2.15	3.53	0	0.664575	Middle SDI
India	2.85	2.75	2.95	0	0.575402	Low-middle SDI
Turkmenistan	2.85	2.27	3.43	0	0.682161	Middle SDI
Guatemala	2.88	2.46	3.3	0	0.539972	Low-middle SDI
Algeria	2.91	2.65	3.17	0	0.659501	Middle SDI
Ecuador	2.92	2.02	3.82	0	0.661017	Middle SDI
Eswatini	2.93	2.25	3.61	0	0.58546	Low-middle SDI
Azerbaijan	2.94	2.63	3.26	0	0.694851	Middle SDI
Saint Kitts and Nevis	2.94	2.63	3.25	0	0.754987	High-middle SDI
Seychelles	2.97	2.73	3.22	0	0.730151	High-middle SDI
Niue	2.99	2.79	3.19	0	0.726222	High-middle SDI
Uzbekistan	3	2.16	3.85	0	0.662622	Middle SDI
Hungary	3.03	2.71	3.36	0	0.790755	High-middle SDI
Saudi Arabia	3.03	2.82	3.25	0	0.815143	High SDI

Dominican Republic	3.04	2.77	3.31	0	0.619388	Middle SDI
South Africa	3.07	2.61	3.53	0	0.679627	Middle SDI
Tunisia	3.1	3.03	3.16	0	0.682432	Middle SDI
Antigua and Barbuda	3.11	2.84	3.39	0	0.749887	High-middle SDI
Philippines	3.11	2.92	3.31	0	0.651219	Middle SDI
Belize	3.12	2.61	3.63	0	0.610229	Low-middle SDI
Nepal	3.13	2.8	3.46	0	0.433175	Low SDI
Venezuela	3.19	2.74	3.65	0	0.596513	Low-middle SDI
Lithuania	3.21	2.72	3.7	0	0.856484	High SDI
Mozambique	3.22	3.08	3.36	0	0.326463	Low SDI
Kazakhstan	3.24	2.86	3.62	0	0.725144	High-middle SDI
Grenada	3.32	3.11	3.53	0	0.668993	Middle SDI
Honduras	3.39	3.14	3.65	0	0.513037	Low-middle SDI
Trinidad and Tobago	3.39	3.02	3.77	0	0.768763	High-middle SDI
Equatorial Guinea	3.42	3.02	3.83	0	0.657857	Middle SDI
Saint Vincent and the Grenadines	3.45	3.1	3.79	0	0.637196	Middle SDI
Cabo Verde	3.46	3.2	3.71	0	0.533535	Low-middle SDI
Germany	3.46	2.98	3.94	0	0.902957	High SDI
Kenya	3.51	3.41	3.61	0	0.523768	Low-middle SDI
American Samoa	3.55	3.29	3.82	0	0.723728	High-middle SDI
Nicaragua	3.6	3.17	4.03	0	0.523958	Low-middle SDI
Botswana	3.61	3.31	3.9	0	0.642722	Middle SDI
Panama	3.62	3.15	4.09	0	0.708865	Middle SDI
Mauritius	3.7	3.27	4.14	0	0.71826	High-middle SDI
Sweden	3.72	3.55	3.88	0	0.88688	High SDI
Bulgaria	3.79	3.32	4.27	0	0.768151	High-middle SDI
Denmark	3.94	3.69	4.19	0	0.896424	High SDI
Ghana	3.94	3.85	4.03	0	0.56493	Low-middle SDI
Libya	4.04	3.78	4.29	0	0.725771	High-middle SDI
El Salvador	4.09	3.58	4.61	0	0.563775	Low-middle SDI
Guyana	4.09	3.71	4.47	0	0.650812	Middle SDI
Cuba	4.17	3.81	4.53	0	0.66873	Middle SDI
Zimbabwe	4.19	3.61	4.78	0	0.473819	Low-middle SDI
United States of America	4.27	4.04	4.49	0	0.862448	High SDI
Estonia	4.54	3.92	5.16	0	0.844918	High SDI
Belarus	4.81	3.92	5.71	0	0.784485	High-middle SDI
Latvia	4.93	4.45	5.42	0	0.830664	High SDI
Oman	5	4.65	5.35	0	0.773392	High-middle SDI
Georgia	5.04	4.36	5.72	0	0.732474	High-middle SDI
Austria	5.18	4.5	5.87	0	0.853837	High SDI

Lesotho	5.55	4.96	6.14	0	0.510393	Low-middle SDI
United Arab Emirates	5.92	5.12	6.73	0	0.849318	High SDI
Armenia	8.7	7.37	10.06	0	0.701833	Middle SDI
Ukraine	14.26	12.24	16.31	0	0.760774	High-middle SDI

Table S4

The high BMI-attributable CKD DALY EAPC for both sexes and all age groups in 204 GBD countries from 1990 to 2021.

location	EAPC	LCI	UCI	Pr	SDIvalue	SDI.Quintile
Poland	-1.5	-1.89	-1.11	0	0.812042809	High SDI
Maldives	-0.98	-1.17	-0.78	0	0.650886627	Middle SDI
Kuwait	-0.76	-0.98	-0.54	0	0.846651055	High SDI
Ethiopia	-0.7	-0.88	-0.51	0	0.358823295	Low SDI
Czechia	-0.67	-0.79	-0.54	0	0.828450433	High SDI
Cyprus	-0.53	-0.7	-0.36	0	0.835630545	High SDI
Rwanda	-0.38	-0.77	0.02	0.069	0.435588706	Low SDI
Slovakia	-0.33	-0.37	-0.29	0	0.81061053	High SDI
South Korea	-0.31	-0.45	-0.17	0	0.886675267	High SDI
Spain	-0.18	-0.28	-0.08	0.002	0.769283698	High-middle SDI
Japan	-0.11	-0.2	-0.01	0.032	0.871241813	High SDI
Andorra	-0.03	-0.2	0.13	0.682	0.869444113	High SDI
Italy	-0.01	-0.11	0.09	0.851	0.805773534	High-middle SDI
Iraq	0.01	-0.12	0.15	0.839	0.662626231	Middle SDI
Mongolia	0.05	-0.07	0.18	0.42	0.617621565	Low-middle SDI
Israel	0.12	-0.24	0.49	0.518	0.809011652	High-middle SDI
Burundi	0.13	-0.05	0.32	0.17	0.289374365	Low SDI
Solomon Islands	0.15	-0.04	0.34	0.13	0.429360316	Low SDI
Niger	0.17	0.06	0.29	0.006	0.168072774	Low SDI
Slovenia	0.27	0.12	0.42	0.002	0.842430731	High SDI
Jordan	0.39	0.08	0.71	0.019	0.725307227	High-middle SDI
Russian Federation	0.41	0.21	0.62	0	0.808536005	High-middle SDI
Greece	0.43	-0.19	1.05	0.186	0.791854408	High-middle SDI
Albania	0.44	0.25	0.62	0	0.706849791	Middle SDI
Portugal	0.46	0.05	0.88	0.036	0.744151851	High-middle SDI
Serbia	0.5	0.41	0.6	0	0.792416294	High-middle SDI
Mauritania	0.51	0.36	0.67	0	0.4989451	Low-middle SDI
Myanmar	0.51	0.43	0.6	0	0.53390084	Low-middle SDI
Argentina	0.53	0.21	0.84	0.003	0.723122973	High-middle SDI
Palestine	0.53	0.34	0.71	0	0.631011665	Middle SDI
San Marino	0.59	0.4	0.77	0	0.888005474	High SDI
Papua New Guinea	0.6	0.54	0.66	0	0.417797443	Low SDI

Colombia	0.62	0.46	0.77	0	0.655442913	Middle SDI
Mali	0.62	0.46	0.77	0	0.268579941	Low SDI
Senegal	0.65	0.53	0.77	0	0.408054193	Low SDI
Bosnia and Herzegovina	0.66	0.49	0.83	0	0.723077893	High-middle SDI
Malta	0.68	0.44	0.93	0	0.801585034	High-middle SDI
Tajikistan	0.7	0.62	0.79	0	0.541511187	Low-middle SDI
Luxembourg	0.73	0.56	0.89	0	0.884428955	High SDI
Romania	0.74	0.44	1.05	0	0.768453864	High-middle SDI
Bermuda	0.76	0.49	1.03	0	0.821365422	High SDI
Greenland	0.77	0.61	0.92	0	0.826210336	High SDI
Brazil	0.8	0.61	1	0	0.653043887	Middle SDI
Ireland	0.81	0.69	0.92	0	0.87375385	High SDI
Cameroon	0.82	0.54	1.09	0	0.479691223	Low-middle SDI
Guinea-Bissau	0.82	0.77	0.88	0	0.353109621	Low SDI
Switzerland	0.82	0.62	1.01	0	0.933059111	High SDI
Qatar	0.84	0.29	1.4	0.006	0.846860584	High SDI
France	0.86	0.76	0.97	0	0.838364875	High SDI
North Macedonia	0.86	0.65	1.07	0	0.750629703	High-middle SDI
United Kingdom	0.86	0.75	0.97	0	0.859000182	High SDI
Turkey	0.88	0.57	1.19	0	0.712692673	High-middle SDI
Bhutan	0.91	0.84	0.97	0	0.473062378	Low-middle SDI
Cambodia	0.93	0.71	1.14	0	0.473621491	Low-middle SDI
Lebanon	0.95	0.76	1.14	0	0.744746351	High-middle SDI
Taiwan (Province of China)	0.97	0.72	1.22	0	0.874747053	High SDI
United States Virgin Islands	0.97	0.75	1.18	0	0.821830853	High SDI
Chad	0.98	0.84	1.11	0	0.240436019	Low SDI
Chile	1.01	0.59	1.42	0	0.771514716	High-middle SDI
Sierra Leone	1.01	0.98	1.04	0	0.358665881	Low SDI
Peru	1.02	0.83	1.22	0	0.662054037	Middle SDI
Congo	1.05	0.91	1.19	0	0.583075236	Low-middle SDI
Croatia	1.06	0.85	1.27	0	0.798341027	High-middle SDI
Finland	1.06	0.9	1.22	0	0.859831368	High SDI
Kyrgyzstan	1.07	0.56	1.57	0	0.603979328	Low-middle SDI
Norway	1.07	0.93	1.21	0	0.91613281	High SDI
Afghanistan	1.15	0.99	1.31	0	0.337199998	Low SDI
Belgium	1.17	0.98	1.37	0	0.853654016	High SDI
Laos	1.18	1.12	1.24	0	0.489136091	Low-middle SDI
Syrian Arab Republic	1.18	0.9	1.45	0	0.623004075	Middle SDI
Côte d'Ivoire	1.21	1.09	1.32	0	0.425941883	Low SDI
Sri Lanka	1.22	1.01	1.42	0	0.701534935	Middle SDI

Montenegro	1.23	1.11	1.35	0	0.795800584	High-middle SDI
Burkina Faso	1.24	1.15	1.33	0	0.285118402	Low SDI
Uruguay	1.24	1.02	1.46	0	0.719283445	High-middle SDI
Cook Islands	1.25	1.17	1.32	0	0.779109955	High-middle SDI
Guinea	1.25	1.2	1.31	0	0.336401293	Low SDI
North Korea	1.31	1.27	1.34	0	0.569854634	Low-middle SDI
Iceland	1.32	1.23	1.4	0	0.87636168	High SDI
Monaco	1.32	1.17	1.48	0	0.908262831	High SDI
Benin	1.33	1.23	1.43	0	0.373486574	Low SDI
Kazakhstan	1.33	1.08	1.58	0	0.725144495	High-middle SDI
Puerto Rico	1.36	1.01	1.71	0	0.825525847	High SDI
Canada	1.39	1.19	1.59	0	0.87317068	High SDI
Germany	1.39	1.18	1.6	0	0.902957091	High SDI
Hungary	1.43	1.2	1.65	0	0.790754768	High-middle SDI
China	1.44	1.32	1.56	0	0.72162976	High-middle SDI
Australia	1.45	1.26	1.64	0	0.844252814	High SDI
Netherlands	1.47	1.25	1.69	0	0.888464256	High SDI
Vanuatu	1.47	1.41	1.52	0	0.473100706	Low-middle SDI
Democratic Republic of the Congo	1.5	1.36	1.63	0	0.383179849	Low SDI
Bahrain	1.53	1.23	1.83	0	0.753043204	High-middle SDI
Brunei Darussalam	1.53	1.39	1.66	0	0.810234367	High-middle SDI
Central African Republic	1.53	1.47	1.58	0	0.30916769	Low SDI
Malaysia	1.54	1.38	1.7	0	0.742523828	High-middle SDI
Yemen	1.54	1.35	1.73	0	0.450376375	Low SDI
Angola	1.55	1.4	1.7	0	0.453721949	Low SDI
Singapore	1.55	1.13	1.97	0	0.856097766	High SDI
Togo	1.55	1.48	1.62	0	0.408533695	Low SDI
New Zealand	1.57	1.23	1.91	0	0.849442499	High SDI
Somalia	1.57	1.5	1.65	0	0.077688109	Low SDI
Moldova	1.6	1.45	1.76	0	0.732214875	High-middle SDI
Gambia	1.62	1.47	1.77	0	0.40971416	Low SDI
Tonga	1.62	1.46	1.79	0	0.626349936	Middle SDI
Liberia	1.63	1.31	1.94	0	0.352442452	Low SDI
Lithuania	1.63	1.44	1.82	0	0.856484049	High SDI
Uganda	1.63	1.43	1.83	0	0.423261181	Low SDI
Azerbaijan	1.67	1.52	1.83	0	0.694851274	Middle SDI
Nigeria	1.68	1.59	1.77	0	0.503390833	Low-middle SDI
Kiribati	1.7	1.47	1.92	0	0.527186583	Low-middle SDI
Samoa	1.71	1.64	1.78	0	0.593392769	Low-middle SDI
Guam	1.75	1.47	2.03	0	0.803982203	High-middle SDI
Iran	1.81	1.71	1.91	0	0.697207398	Middle SDI

Paraguay	1.85	1.74	1.97	0	0.635718099	Middle SDI
Saint Lucia	1.86	1.6	2.11	0	0.672509735	Middle SDI
Belarus	1.87	1.59	2.14	0	0.784484711	High-middle SDI
Bolivia	1.88	1.79	1.97	0	0.599010799	Low-middle SDI
Nauru	1.94	1.81	2.07	0	0.625177834	Middle SDI
Malawi	1.95	1.72	2.19	0	0.384553634	Low SDI
Timor-Leste	1.95	1.62	2.29	0	0.444667619	Low SDI
Northern Mariana Islands	1.96	1.78	2.14	0	0.771535213	High-middle SDI
Madagascar	1.98	1.85	2.11	0	0.400246943	Low SDI
Sweden	1.98	1.85	2.1	0	0.886880299	High SDI
Tokelau	2	1.86	2.15	0	0.686425621	Middle SDI
United Republic of Tanzania	2	1.95	2.04	0	0.446568273	Low SDI
Namibia	2.02	1.68	2.36	0	0.617564872	Low-middle SDI
Comoros	2.03	1.91	2.16	0	0.475978688	Low-middle SDI
Jamaica	2.03	1.44	2.63	0	0.683263064	Middle SDI
Marshall Islands	2.04	1.82	2.26	0	0.574091128	Low-middle SDI
Turkmenistan	2.05	1.72	2.39	0	0.682160776	Middle SDI
Denmark	2.08	1.94	2.22	0	0.896424204	High SDI
South Sudan	2.11	1.85	2.36	0	0.278371125	Low SDI
Micronesia	2.12	1.79	2.45	0	0.587534967	Low-middle SDI
Zambia	2.12	1.95	2.3	0	0.505948954	Low-middle SDI
Haiti	2.17	2.03	2.3	0	0.448278285	Low SDI
Uzbekistan	2.17	1.7	2.64	0	0.662621694	Middle SDI
Barbados	2.18	1.92	2.44	0	0.746748764	High-middle SDI
Palau	2.18	1.99	2.36	0	0.754046931	High-middle SDI
Tuvalu	2.18	2.09	2.26	0	0.576620529	Low-middle SDI
Fiji	2.19	1.88	2.51	0	0.675051631	Middle SDI
Latvia	2.2	2.01	2.39	0	0.830663516	High SDI
Sudan	2.24	2.06	2.42	0	0.541949735	Low-middle SDI
Gabon	2.26	2.03	2.49	0	0.634691393	Middle SDI
Sao Tome and Principe	2.27	2.16	2.38	0	0.505413747	Low-middle SDI
Suriname	2.29	2.1	2.47	0	0.633665739	Middle SDI
Costa Rica	2.3	1.94	2.66	0	0.700340477	Middle SDI
Algeria	2.32	2.12	2.52	0	0.659500924	Middle SDI
Eritrea	2.35	2.29	2.4	0	0.403863943	Low SDI
Saint Kitts and Nevis	2.35	2.05	2.66	0	0.754987055	High-middle SDI
Bangladesh	2.39	2.21	2.58	0	0.492420885	Low-middle SDI
Georgia	2.4	2.05	2.75	0	0.732473604	High-middle SDI
Seychelles	2.41	2.2	2.63	0	0.730150775	High-middle SDI
Dominica	2.42	2.33	2.52	0	0.746967185	High-middle SDI

Morocco	2.43	2.32	2.55	0	0.562698301	Low-middle SDI
Bahamas	2.44	2.24	2.64	0	0.805020668	High-middle SDI
Estonia	2.44	2.04	2.84	0	0.844917787	High SDI
Thailand	2.47	2.26	2.69	0	0.682547933	Middle SDI
South Africa	2.48	2.12	2.84	0	0.679626598	Middle SDI
Djibouti	2.5	2.38	2.61	0	0.487958371	Low-middle SDI
Venezuela	2.51	2.11	2.91	0	0.596513059	Low-middle SDI
Egypt	2.52	2.42	2.62	0	0.606787094	Low-middle SDI
Niue	2.54	2.37	2.71	0	0.72622205	High-middle SDI
Pakistan	2.56	2.34	2.78	0	0.504028689	Low-middle SDI
Tunisia	2.57	2.54	2.6	0	0.682432216	Middle SDI
Ecuador	2.61	1.76	3.46	0	0.661017053	Middle SDI
Ukraine	2.64	2.38	2.91	0	0.760773913	High-middle SDI
Antigua and Barbuda	2.67	2.42	2.93	0	0.749886887	High-middle SDI
Nepal	2.67	2.39	2.95	0	0.433174635	Low SDI
Honduras	2.68	2.51	2.85	0	0.513037248	Low-middle SDI
Saudi Arabia	2.69	2.47	2.91	0	0.815143493	High SDI
Cabo Verde	2.78	2.62	2.93	0	0.533534539	Low-middle SDI
Indonesia	2.79	2.64	2.93	0	0.656868336	Middle SDI
India	2.81	2.76	2.86	0	0.575401649	Low-middle SDI
Equatorial Guinea	2.83	2.47	3.19	0	0.657857456	Middle SDI
Viet Nam	2.84	2.6	3.08	0	0.627933721	Middle SDI
Belize	2.95	2.51	3.4	0	0.610229002	Low-middle SDI
Eswatini	2.95	2.26	3.64	0	0.585459713	Low-middle SDI
Bulgaria	2.97	2.63	3.3	0	0.768150939	High-middle SDI
Austria	2.99	2.59	3.39	0	0.853837004	High SDI
Grenada	3.02	2.82	3.22	0	0.668993028	Middle SDI
Mexico	3.02	2.34	3.71	0	0.664575304	Middle SDI
Botswana	3.04	2.8	3.27	0	0.642721629	Middle SDI
Dominican Republic	3.04	2.83	3.25	0	0.619388201	Middle SDI
Panama	3.1	2.74	3.47	0	0.708864828	Middle SDI
American Samoa	3.19	2.95	3.43	0	0.723727533	High-middle SDI
Philippines	3.21	3.03	3.39	0	0.651219329	Middle SDI
Trinidad and Tobago	3.22	2.9	3.55	0	0.768763254	High-middle SDI
Mozambique	3.24	3.1	3.38	0	0.326462614	Low SDI
Armenia	3.25	2.92	3.59	0	0.701833194	Middle SDI
Saint Vincent and the Grenadines	3.28	2.99	3.58	0	0.637195963	Middle SDI
Guatemala	3.29	2.84	3.74	0	0.539972424	Low-middle SDI
Cuba	3.32	3.02	3.62	0	0.668729864	Middle SDI
Nicaragua	3.39	3	3.78	0	0.523958472	Low-middle SDI
Kenya	3.4	3.31	3.5	0	0.523768077	Low-middle SDI

Mauritius	3.52	3.09	3.95	0	0.718260446	High-middle SDI
United States of America	3.52	3.32	3.73	0	0.862448354	High SDI
Libya	3.69	3.47	3.9	0	0.725771399	High-middle SDI
Ghana	3.72	3.63	3.81	0	0.56493039	Low-middle SDI
Guyana	3.88	3.52	4.23	0	0.650812335	Middle SDI
El Salvador	3.97	3.5	4.44	0	0.563775188	Low-middle SDI
Zimbabwe	4.03	3.45	4.62	0	0.473819486	Low-middle SDI
Oman	4.33	4.07	4.58	0	0.773391602	High-middle SDI
United Arab Emirates	4.48	3.89	5.07	0	0.849317734	High SDI
Lesotho	5.1	4.58	5.61	0	0.510393066	Low-middle SDI

Table S5

The ASR of high BMI-attributable CKD for both sexes in all age groups from 1990 to 2021 globally.

location	sex	year	val	upper	lower
Global	Male	1990	72.45329	114.7228	35.96
Global	Female	1990	67.03992	101.1879	34.78133
Global	Male	1991	73.11268	115.7026	36.51405
Global	Female	1991	67.78342	101.3639	35.32621
Global	Male	1992	74.00997	116.966	37.06771
Global	Female	1992	68.83362	102.3462	36.26007
Global	Male	1993	75.12467	118.7952	37.86042
Global	Female	1993	69.93535	104.2303	37.10125
Global	Male	1994	76.10101	120.105	38.15056
Global	Female	1994	71.03969	105.8398	37.63664
Global	Male	1995	77.64421	122.8861	39.03719
Global	Female	1995	72.21912	107.9154	38.00853
Global	Male	1996	78.99029	124.3147	39.82607
Global	Female	1996	73.6035	109.0945	39.0295
Global	Male	1997	80.52526	126.7457	40.73188
Global	Female	1997	75.34805	110.9438	40.03448
Global	Male	1998	83.29188	130.6485	42.32451
Global	Female	1998	78.23769	114.9924	41.38961
Global	Male	1999	86.05668	134.5913	43.40478
Global	Female	1999	81.04233	119.0948	43.06252
Global	Male	2000	89.3798	138.6743	45.32572
Global	Female	2000	84.05251	124.0917	44.64538
Global	Male	2001	91.71268	142.0875	46.27957
Global	Female	2001	86.29543	127.4292	45.56446
Global	Male	2002	93.89081	145.3762	47.39295
Global	Female	2002	88.30032	130.3692	46.81204
Global	Male	2003	96.52162	149.276	48.60273
Global	Female	2003	90.29112	133.5365	47.23732
Global	Male	2004	98.18847	151.0126	49.51496
Global	Female	2004	91.51098	135.8538	48.11786
Global	Male	2005	99.88491	152.0992	50.68258
Global	Female	2005	92.87373	137.2561	48.60625
Global	Male	2006	101.1183	153.6535	51.32731
Global	Female	2006	93.75381	138.1986	49.25569
Global	Male	2007	102.9538	156.7036	52.91457
Global	Female	2007	95.03439	141.0911	49.96547
Global	Male	2008	105.3956	159.4823	54.2148
Global	Female	2008	96.76427	143.2296	50.96251
Global	Male	2009	107.3232	162.9932	55.15902
Global	Female	2009	98.35219	144.3549	51.42091

Global	Male	2010	109.4527	166.5569	56.70195
Global	Female	2010	99.7772	146.4936	52.267
Global	Male	2011	111.9938	168.7709	57.81756
Global	Female	2011	101.4519	148.8107	53.19346
Global	Male	2012	113.8784	172.3172	58.7991
Global	Female	2012	102.9532	152.6538	54.01459
Global	Male	2013	116.6059	174.9261	60.11144
Global	Female	2013	104.754	153.7385	55.13002
Global	Male	2014	118.4548	178.4747	61.71328
Global	Female	2014	106.4683	157.3289	56.31886
Global	Male	2015	121.1271	181.1436	63.33587
Global	Female	2015	108.8768	159.9565	57.91095
Global	Male	2016	123.2349	185.1339	64.40512
Global	Female	2016	110.831	161.8232	58.65745
Global	Male	2017	123.7364	186.6326	64.67508
Global	Female	2017	111.4291	164.0307	59.51571
Global	Male	2018	124.1461	186.2996	65.07258
Global	Female	2018	111.8841	163.576	59.98348
Global	Male	2019	125.5823	187.915	65.52622
Global	Female	2019	113.1814	165.127	60.73071
Global	Male	2020	127.2537	189.635	67.74829
Global	Female	2020	114.9129	168.0771	61.86172
Global	Male	2021	128.5842	190.8964	68.10333
Global	Female	2021	116.9777	170.9371	63.77652

Table S6

The death and DALY of high BMI-attributable CKD for both sexes and 25-95+ years age groups in global from 2021.

sex	Age (years)	metric	Deaths			DALY		
			val	upper	lower	val	upper	lower
Male	25-29	Number	1110.165	1836.027	505.5272	89006.91	148606.5	41564.22
Female	25-29	Number	755.1094	1268.687	319.8199	65090.57	111303.3	28071.75
Male	25-29	Rate	0.373345	0.617451	0.170007	29.93278	49.97598	13.97793
Female	25-29	Rate	0.259499	0.435994	0.109909	22.36887	38.25024	9.647072
Male	30-34	Number	1470.921	2385.982	706.2332	113166.9	180487.6	56416.04
Female	30-34	Number	1158.473	1876.532	551.9961	93081.86	151621.8	48048.68
Male	30-34	Rate	0.481402	0.780883	0.231136	37.03719	59.06988	18.46381
Female	30-34	Rate	0.387539	0.627748	0.184657	31.13826	50.72137	16.07351
Male	35-39	Number	2360.458	3805.446	1207.198	161127.5	255085.1	84146.61
Female	35-39	Number	1768.921	2760.689	911.923	129247	201642.5	66142.64
Male	35-39	Rate	0.833897	1.344379	0.426476	56.92275	90.1159	29.72713
Female	35-39	Rate	0.636754	0.993759	0.328263	46.52476	72.58479	23.80921
Male	40-44	Number	3701.511	5788.827	1804.497	221144.4	336438.7	111021.4
Female	40-44	Number	2969.992	4587.712	1583.526	188422.8	285391	97964.88
Male	40-44	Rate	1.467916	2.295688	0.715613	87.69971	133.4222	44.02798
Female	40-44	Rate	1.197143	1.849214	0.638287	75.9494	115.0353	39.48766
Male	45-49	Number	5578.596	8563.168	2845.497	298367.1	450654.2	152092.1
Female	45-49	Number	5098.244	7668.607	2627.947	280812.9	424541.5	147832.5
Male	45-49	Rate	2.345307	3.600056	1.19628	125.437	189.4603	63.94131
Female	45-49	Rate	2.16355	3.254338	1.115226	119.169	180.1633	62.73589
Male	50-54	Number	9802.177	14850.64	5313.652	454031.8	676935.4	240352.7
Female	50-54	Number	8868.843	13189.11	5078.59	420727.5	630038.9	235691.8
Male	50-54	Rate	4.415785	6.690068	2.393748	204.5369	304.9528	108.2766
Female	50-54	Rate	3.978084	5.915923	2.277981	188.7156	282.6014	105.7186
Male	55-59	Number	14646.25	22344.37	7722.206	587848.2	878777.1	321683.2
Female	55-59	Number	14143.6	20810.28	7906.158	575787.1	849273	321045.7
Male	55-59	Rate	7.521522	11.47487	3.965709	301.8872	451.2926	165.1992
Female	55-59	Rate	7.036494	10.35319	3.933343	286.4562	422.5165	159.7214
Male	60-64	Number	18666.14	28828.84	10227.05	636147.3	970772.3	345482.1
Female	60-64	Number	18207.83	26626.56	10516.45	632221.9	935166.9	358716.3
Male	60-64	Rate	12.0011	18.53505	6.575318	409.0009	624.1426	222.1223
Female	60-64	Rate	11.06785	16.18528	6.392553	384.3039	568.4527	218.0501
Male	65-69	Number	22193.55	33449.84	12276.75	636394.2	961170.1	352242
Female	65-69	Number	23882.91	34730.88	13441.11	695876.5	1023012	396443.6
Male	65-69	Rate	16.83456	25.37284	9.312333	482.7266	729.0802	267.1875
Female	65-69	Rate	16.58433	24.11717	9.333527	483.2176	710.3808	275.291
Male	70-74	Number	25269.81	39164.06	13498.26	593282	919141.4	316921.1
Female	70-74	Number	27016.58	39526.59	14571.77	647523.2	944975	349765.2

Male	70-74	Rate	26.21583	40.63024	14.00359	615.4925	953.5511	328.7856
Female	70-74	Rate	24.68445	36.11456	13.3139	591.6274	863.4024	319.5726
Male	75-79	Number	24752.65	39070.42	12703.1	467473.7	747086.9	236785.4
Female	75-79	Number	27335.01	41206.85	14118.2	524573.2	792330.1	273006.2
Male	75-79	Rate	41.40158	65.34965	21.24737	781.9023	1249.587	396.0501
Female	75-79	Rate	37.91386	57.1542	19.58205	727.5868	1098.967	378.6616
Male	80-84	Number	23713.79	38206.07	11522.11	350976.8	569787.4	170651.7
Female	80-84	Number	27232.24	42306.61	13352.33	412874.6	651999.5	200639.6
Male	80-84	Rate	64.70009	104.2405	31.43662	957.5961	1554.593	465.6018
Female	80-84	Rate	53.46869	83.06619	26.21641	810.6518	1280.157	393.9424
Male	85-89	Number	22638.06	36630.63	10968.72	260604.4	425547.8	126694.3
Female	85-89	Number	28478.39	44719.12	13775.98	336196.2	524782	162784.4
Male	85-89	Rate	131.2149	212.3187	63.57698	1510.517	2466.564	734.3466
Female	85-89	Rate	100.0325	157.0793	48.3892	1180.915	1843.337	571.7925
Male	90-94	Number	14252.73	22512.36	7264.19	142849.2	226661.4	72884.39
Female	90-94	Number	22714.73	35440.27	10856.92	233860.1	368050.8	112247.5
Male	90-94	Rate	244.5351	386.2463	124.6323	2450.874	3888.847	1250.483
Female	90-94	Rate	188.334	293.845	90.01768	1938.997	3051.61	930.6747
Male	95+	Number	5452.871	8621.664	2595.089	51069.24	80766.58	25016.36
Female	95+	Number	13161.11	20256.29	6323.627	122776.5	190995.6	59631.11
Male	95+	Rate	360.6296	570.2	171.6281	3377.502	5341.557	1654.475
Female	95+	Rate	334.1853	514.3453	160.5688	3117.527	4849.736	1514.146

Table S7

The high BMI-attributable CKD death causes for both sexes and all age groups in global from 1990 to 2021.

year	sex	cause	proportion	val	upper	lower
2021	Male	diabetes mellitus type 2	45.46855	88940.88	131307.7	44215.88
2021	Female	diabetes mellitus type 2	40.77678	90847.4	131912.7	47294.65
2021	Male	glomerulonephritis	5.389587	10542.55	20738.36	3297.455
2021	Female	glomerulonephritis	5.534933	12331.39	23029.69	4120.174
2021	Male	hypertension	40.70417	79621.3	137065.6	33880.9
2021	Female	hypertension	42.03104	93641.79	150923.3	42092.13
2021	Male	other and unspecified causes	8.437688	16504.93	33908.31	5332.931
2021	Female	other and unspecified causes	11.65724	25971.41	50220.29	8702.081
2020	Male	diabetes mellitus type 2	40.4471	76896.92	132657.2	33375.91
2020	Female	diabetes mellitus type 2	41.58172	89385.55	144218.6	39315.57
2020	Male	hypertension	45.66278	86812.82	128246.4	43082.08
2020	Female	hypertension	41.09006	88328.66	127851.8	45768.07
2020	Male	glomerulonephritis	8.491684	16144.16	33353.35	5180.39
2020	Female	glomerulonephritis	11.79264	25349.88	49423.64	8479.264
2020	Male	other and unspecified causes	5.398444	10263.37	20047.6	3249.442
2020	Female	other and unspecified causes	5.53558	11899.48	22367.82	3936.01
2019	Male	diabetes mellitus type 2	40.30816	73737.26	127960.5	30762.93
2019	Female	diabetes mellitus type 2	41.68171	86390.97	138228.1	38714.63
2019	Male	hypertension	45.81323	83807.9	124633.1	41668.4
2019	Female	hypertension	41.09388	85172.61	123064.3	44234.17
2019	Male	glomerulonephritis	8.489821	15530.76	32210.28	5010.923
2019	Female	glomerulonephritis	11.76176	24377.84	47609.06	8068.445
2019	Male	other and unspecified causes	5.388792	9857.925	19526.5	3061.043
2019	Female	other and unspecified causes	5.462648	11322.07	21561.69	3733.728
2018	Male	diabetes mellitus type 2	40.33115	70472.05	120804	29671.74
2018	Female	diabetes mellitus type 2	41.63238	82545.35	133651	37176.55
2018	Male	hypertension	45.77512	79984.51	121087.2	39526.47
2018	Female	hypertension	41.06975	81429.81	119036.3	42398.24
2018	Male	glomerulonephritis	8.489663	14834.29	30853.86	4814.92
2018	Female	glomerulonephritis	11.80311	23402.27	45491.36	7749.962
2018	Male	other and unspecified causes	5.404064	9442.714	18759.49	2919.562
2018	Female	other and unspecified causes	5.494748	10894.55	20803	3576.358
2017	Male	diabetes mellitus type 2	40.39119	67954.67	118687.5	28972.32
2017	Female	diabetes mellitus type 2	41.55109	79410.23	128166.5	35399.62
2017	Male	hypertension	45.66926	76834.58	115236.1	38359.59
2017	Female	hypertension	41.07994	78509.78	113067.2	40596.32
2017	Male	glomerulonephritis	8.486904	14278.48	29789.43	4619.203
2017	Female	glomerulonephritis	11.82241	22594.36	43790.64	7439.772
2017	Male	other and unspecified causes	5.452645	9173.603	18338.07	2860.066

2017	Female	other and unspecified causes	5.546558	10600.29	20321.97	3476.547
2016	Male	diabetes mellitus type 2	40.44608	65430.79	114081.4	28065.34
2016	Female	diabetes mellitus type 2	41.47913	76209.2	122018.8	33855.17
2016	Male	hypertension	45.60996	73784.55	111409.5	36852.93
2016	Female	hypertension	41.10424	75520.42	109820.4	39128.16
2016	Male	glomerulonephritis	8.479531	13717.58	28642.35	4408.723
2016	Female	glomerulonephritis	11.86194	21793.83	42204.25	7162.352
2016	Male	other and unspecified causes	5.464424	8839.957	17735.79	2716.052
2016	Female	other and unspecified causes	5.554692	10205.58	19597.18	3310.325
2015	Male	diabetes mellitus type 2	40.53375	62273.6	107248.5	26429.68
2015	Female	diabetes mellitus type 2	41.50986	72451.34	116004.3	31775.42
2015	Male	hypertension	45.56796	70007.84	105648.8	34816.44
2015	Female	hypertension	41.09637	71729.64	103578.7	37063.41
2015	Male	glomerulonephritis	8.469537	13012.08	27243.11	4132.037
2015	Female	glomerulonephritis	11.87815	20732.14	40208.28	6839.178
2015	Male	other and unspecified causes	5.428751	8340.404	16795.26	2554.208
2015	Female	other and unspecified causes	5.515621	9626.969	18506.21	3121.447
2014	Male	diabetes mellitus type 2	40.60239	58979.37	100265.7	25194.27
2014	Female	diabetes mellitus type 2	41.63886	68825.96	109890.3	30335.4
2014	Male	hypertension	45.53961	66151.22	100318.4	32861.28
2014	Female	hypertension	41.102	67938.58	99058.52	34859.03
2014	Male	glomerulonephritis	8.460883	12290.35	25809.77	3911.727
2014	Female	glomerulonephritis	11.79867	19502.33	37874.71	6430.071
2014	Male	other and unspecified causes	5.397119	7839.901	15816.46	2392.165
2014	Female	other and unspecified causes	5.460479	9025.77	17341.1	2918.967
2013	Male	diabetes mellitus type 2	40.60327	56149.17	97529.62	23680.34
2013	Female	diabetes mellitus type 2	41.68913	65583.12	104579.6	28967.24
2013	Male	hypertension	45.491	62908.28	96808.84	31230.66
2013	Female	hypertension	41.05359	64583.31	93715.39	33325.3
2013	Male	glomerulonephritis	8.494659	11747.03	24602.98	3714.615
2013	Female	glomerulonephritis	11.7947	18554.79	36183.19	6146.222
2013	Male	other and unspecified causes	5.411066	7482.817	15256.38	2272.139
2013	Female	other and unspecified causes	5.462585	8593.448	16502.12	2779.211
2012	Male	diabetes mellitus type 2	40.55627	52859.06	90339.75	22481.47
2012	Female	diabetes mellitus type 2	41.71994	62429.28	99885.52	27344.32
2012	Male	hypertension	45.41808	59195.7	91091.3	29525.68
2012	Female	hypertension	40.97436	61313.61	89218.61	31366.01
2012	Male	glomerulonephritis	8.608914	11220.44	23484.84	3518.163
2012	Female	glomerulonephritis	11.8588	17745.39	34868.1	5872.513
2012	Male	other and unspecified causes	5.416739	7059.913	14465.24	2147.269
2012	Female	other and unspecified causes	5.446893	8150.675	15823.78	2635.359
2011	Male	diabetes mellitus type 2	40.54253	50210.82	86277.24	21160.23
2011	Female	diabetes mellitus type 2	41.82981	59721.78	95631.11	26345.21
2011	Male	hypertension	45.33133	56141.62	85686.53	27616.64

2011	Female	hypertension	40.88161	58368	85164.96	30019.99
2011	Male	glomerulonephritis	8.689597	10761.83	22689.3	3356.144
2011	Female	glomerulonephritis	11.85805	16930.12	33435.3	5614.699
2011	Male	other and unspecified causes	5.436549	6733.018	13667.37	2056.695
2011	Female	other and unspecified causes	5.430532	7753.347	14941.48	2495.405
2010	Male	diabetes mellitus type 2	40.53891	47351.41	83140.81	20195.59
2010	Female	diabetes mellitus type 2	41.81981	56599.44	91013.06	24944.69
2010	Male	hypertension	45.15044	52737.9	81460.8	25796.63
2010	Female	hypertension	40.76387	55170.31	80928.66	28292.19
2010	Male	glomerulonephritis	8.796267	10274.47	21691.26	3204.276
2010	Female	glomerulonephritis	11.93628	16154.71	32104.54	5359.54
2010	Male	other and unspecified causes	5.514384	6441.068	13222.17	1945.97
2010	Female	other and unspecified causes	5.48004	7416.752	14374.34	2370.298
2009	Male	diabetes mellitus type 2	40.54675	44802.99	78188.63	19104.94
2009	Female	diabetes mellitus type 2	41.84298	53814.1	85179.52	23836.22
2009	Male	hypertension	45.00959	49734.3	77267.69	24469.5
2009	Female	hypertension	40.64596	52274.63	76927.11	26691.15
2009	Male	glomerulonephritis	8.866698	9797.447	20578.07	3067.824
2009	Female	glomerulonephritis	11.9835	15411.93	30430.98	5107.591
2009	Male	other and unspecified causes	5.57697	6162.392	12682.7	1867.629
2009	Female	other and unspecified causes	5.527562	7108.977	13800.28	2276.209
2008	Male	diabetes mellitus type 2	40.53849	42505.83	73832	17911.19
2008	Female	diabetes mellitus type 2	41.98032	51342.69	81841.51	22537.39
2008	Male	hypertension	44.84478	47021.11	72452.54	22913.17
2008	Female	hypertension	40.45454	49476.63	72986.58	25520.13
2008	Male	glomerulonephritis	8.975457	9411.038	19807.45	2946.477
2008	Female	glomerulonephritis	12.03049	14713.5	29205.68	4865.262
2008	Male	other and unspecified causes	5.641268	5915.04	12193.62	1792.731
2008	Female	other and unspecified causes	5.534656	6768.984	13150.63	2156.918
2007	Male	diabetes mellitus type 2	40.60895	40195.46	69914.07	17172.17
2007	Female	diabetes mellitus type 2	42.15954	48944.58	78572.08	21371
2007	Male	hypertension	44.75401	44298.32	68335.04	21548.18
2007	Female	hypertension	40.29491	46779.86	69201.45	24036.11
2007	Male	glomerulonephritis	9.025766	8933.866	18795.03	2783.103
2007	Female	glomerulonephritis	12.04785	13986.8	27864.98	4632.465
2007	Male	other and unspecified causes	5.611278	5554.144	11424.51	1682.025
2007	Female	other and unspecified causes	5.497708	6382.494	12414.33	2023.106
2006	Male	diabetes mellitus type 2	40.76512	38338.56	66129.72	16232.68
2006	Female	diabetes mellitus type 2	42.42076	47035.88	75417.72	20767.64
2006	Male	hypertension	44.63091	41974.23	65296.67	20182.15
2006	Female	hypertension	40.1219	44486.92	66101.8	22743.18
2006	Male	glomerulonephritis	9.022563	8485.491	17857.54	2626.308
2006	Female	glomerulonephritis	12.00668	13312.93	26491.42	4397.896
2006	Male	other and unspecified causes	5.581417	5249.181	10737	1583.186

2006	Female	other and unspecified causes	5.450658	6043.656	11811.4	1899.693
2005	Male	diabetes mellitus type 2	41.08435	37055.41	64571.89	15680.69
2005	Female	diabetes mellitus type 2	42.76198	45615.35	72970.24	20024.19
2005	Male	hypertension	44.37993	40027.81	62342.61	18894.85
2005	Female	hypertension	39.85616	42515.64	63378.04	21581.33
2005	Male	glomerulonephritis	8.990309	8108.675	17037.67	2500.961
2005	Female	glomerulonephritis	11.95458	12752.27	25441.46	4209.421
2005	Male	other and unspecified causes	5.545415	5001.604	10238.1	1494.968
2005	Female	other and unspecified causes	5.427283	5789.429	11292.55	1817.351
2004	Male	diabetes mellitus type 2	41.24841	35555.92	62006.3	14763.99
2004	Female	diabetes mellitus type 2	42.93724	43872.73	70272.02	19448.37
2004	Male	hypertension	44.2862	38174.48	60761.01	17915.5
2004	Female	hypertension	39.72921	40594.8	60423.53	20680.19
2004	Male	glomerulonephritis	8.932724	7699.963	16118.9	2356.7
2004	Female	glomerulonephritis	11.91565	12175.26	24315.02	4059.653
2004	Male	other and unspecified causes	5.532672	4769.135	9774.307	1424.6
2004	Female	other and unspecified causes	5.417902	5535.944	10803.67	1751.837
2003	Male	diabetes mellitus type 2	41.16303	33942.49	58795.37	14308.58
2003	Female	diabetes mellitus type 2	42.93594	42203.03	67221.43	18724.16
2003	Male	hypertension	44.25405	36491.29	57733.58	17201.19
2003	Female	hypertension	39.57452	38898.99	58288.95	19819.66
2003	Male	glomerulonephritis	8.975462	7401.046	15506.36	2267.581
2003	Female	glomerulonephritis	11.99716	11792.37	23565.84	3931.818
2003	Male	other and unspecified causes	5.607458	4623.835	9412.579	1376.888
2003	Female	other and unspecified causes	5.492376	5398.622	10532.1	1727.017
2002	Male	diabetes mellitus type 2	41.09872	32037.32	55664.37	13531.04
2002	Female	diabetes mellitus type 2	42.97829	40233.6	63700.33	17775.24
2002	Male	hypertension	44.24964	34493.52	55011.5	16137.95
2002	Female	hypertension	39.4172	36899.93	55284.38	18919.71
2002	Male	glomerulonephritis	9.010187	7023.63	14675.5	2161.833
2002	Female	glomerulonephritis	12.06947	11298.69	22443.49	3788.47
2002	Male	other and unspecified causes	5.641448	4397.627	9017.097	1314.62
2002	Female	other and unspecified causes	5.535041	5181.561	10028.91	1666.178
2001	Male	diabetes mellitus type 2	41.1366	30422.84	52805.81	12912.47
2001	Female	diabetes mellitus type 2	43.11073	38306.75	61112.28	17237.69
2001	Male	hypertension	44.15756	32657.01	52616.98	15262.6
2001	Female	hypertension	39.21485	34845	52219.14	17861.3
2001	Male	glomerulonephritis	9.025061	6674.542	13935.86	2058.515
2001	Female	glomerulonephritis	12.09581	10747.93	21354	3611.562
2001	Male	other and unspecified causes	5.680778	4201.256	8601.551	1257.505
2001	Female	other and unspecified causes	5.578615	4956.971	9615.134	1603.646
2000	Male	diabetes mellitus type 2	41.2272	28817.24	49801.71	12228.99
2000	Female	diabetes mellitus type 2	43.26558	36242.72	57463.31	16379.88
2000	Male	hypertension	44.00306	30757.53	50178.57	14430.95

2000	Female	hypertension	38.97111	32645.33	48834.19	16683.21
2000	Male	glomerulonephritis	9.049433	6325.428	13110.91	1953.779
2000	Female	glomerulonephritis	12.13186	10162.62	20164.06	3429.95
2000	Male	other and unspecified causes	5.720311	3998.418	8243.848	1197.435
2000	Female	other and unspecified causes	5.631453	4717.356	9173.338	1542.032
1999	Male	diabetes mellitus type 2	41.5012	26997.93	46775.29	11483.03
1999	Female	diabetes mellitus type 2	43.58936	33811.5	53448.16	15198.16
1999	Male	hypertension	43.75664	28465.16	46709.77	13281
1999	Female	hypertension	38.65194	29981.63	45230.35	15231.72
1999	Male	glomerulonephritis	9.042009	5882.13	12161.48	1825.141
1999	Female	glomerulonephritis	12.11854	9400.137	18540.89	3205.372
1999	Male	other and unspecified causes	5.700151	3708.14	7556.649	1114.209
1999	Female	other and unspecified causes	5.640162	4374.974	8537.742	1439.391
1998	Male	diabetes mellitus type 2	41.77188	25508.46	44567.48	10805.45
1998	Female	diabetes mellitus type 2	43.8007	31791.1	50278.1	14385.32
1998	Male	hypertension	43.55199	26595.51	44082.52	12313.93
1998	Female	hypertension	38.47883	27928.43	42338.01	14092.54
1998	Male	glomerulonephritis	9.006561	5499.957	11416.07	1708.579
1998	Female	glomerulonephritis	12.10254	8784.181	17294.79	2999.583
1998	Male	other and unspecified causes	5.669568	3462.185	7065.833	1044.062
1998	Female	other and unspecified causes	5.617928	4077.564	7985.325	1337.613
1997	Male	diabetes mellitus type 2	42.07205	24092.55	41639.87	10198.89
1997	Female	diabetes mellitus type 2	44.1832	29963.61	47461.09	13463.59
1997	Male	hypertension	43.40308	24854.77	41552.95	11397.18
1997	Female	hypertension	38.33414	25996.97	39419.89	13032.44
1997	Male	glomerulonephritis	8.942826	5121.108	10674.77	1599.04
1997	Female	glomerulonephritis	11.96405	8113.627	15967.73	2774.509
1997	Male	other and unspecified causes	5.582046	3196.557	6544.804	970.2425
1997	Female	other and unspecified causes	5.518614	3742.545	7380.974	1210.578
1996	Male	diabetes mellitus type 2	42.31072	23109.74	40362.09	9690.832
1996	Female	diabetes mellitus type 2	44.50392	28701.66	45395.76	12744.03
1996	Male	hypertension	43.14236	23563.98	39369.28	10924.7
1996	Female	hypertension	38.06048	24546.13	37548.17	12281.29
1996	Male	glomerulonephritis	8.940097	4883.002	10157.69	1538.023
1996	Female	glomerulonephritis	11.9066	7678.856	15080.48	2596.868
1996	Male	other and unspecified causes	5.606821	3062.396	6268.516	935.4652
1996	Female	other and unspecified causes	5.529	3565.787	7003.151	1135.619
1995	Male	diabetes mellitus type 2	42.49388	22198.95	38735.3	9368.309
1995	Female	diabetes mellitus type 2	44.71477	27516.22	43680.79	12234.72
1995	Male	hypertension	42.9241	22423.7	37523.01	10252.63
1995	Female	hypertension	37.81737	23271.75	35632.45	11585.15
1995	Male	glomerulonephritis	8.941055	4670.839	9693.033	1474.513
1995	Female	glomerulonephritis	11.90292	7324.725	14426.18	2447.839
1995	Male	other and unspecified causes	5.640966	2946.861	6036.451	884.2621

1995	Female	other and unspecified causes	5.564944	3424.512	6724.557	1082.513
1994	Male	diabetes mellitus type 2	42.60926	21192.01	36527.8	8938.172
1994	Female	diabetes mellitus type 2	44.84408	26370.6	41907.55	11895.89
1994	Male	hypertension	42.81861	21296.14	35745.64	9715.064
1994	Female	hypertension	37.71421	22177.87	33818.9	10946.42
1994	Male	glomerulonephritis	8.88725	4420.136	9174.002	1398.803
1994	Female	glomerulonephritis	11.84479	6965.336	13733.13	2320.713
1994	Male	other and unspecified causes	5.68488	2827.415	5807.163	846.9836
1994	Female	other and unspecified causes	5.596923	3291.275	6462.858	1027.344
1993	Male	diabetes mellitus type 2	42.78151	20439.65	35355.69	8640.053
1993	Female	diabetes mellitus type 2	44.98332	25347.03	40791.72	11363.52
1993	Male	hypertension	42.6315	20367.97	34488.69	9223.269
1993	Female	hypertension	37.59048	21181.34	32451.51	10452.68
1993	Male	glomerulonephritis	8.919021	4261.225	8823.476	1351.799
1993	Female	glomerulonephritis	11.85341	6679.116	13199	2218.323
1993	Male	other and unspecified causes	5.667974	2707.978	5558.291	807.7659
1993	Female	other and unspecified causes	5.572796	3140.138	6134.483	986.895
1992	Male	diabetes mellitus type 2	42.91064	19626.44	34337.19	8395.287
1992	Female	diabetes mellitus type 2	44.99364	24272.86	38919.45	10872.49
1992	Male	hypertension	42.41986	19401.97	32838.56	8783.361
1992	Female	hypertension	37.47483	20216.67	31218.13	9973.171
1992	Male	glomerulonephritis	8.997838	4115.425	8504.056	1307.804
1992	Female	glomerulonephritis	11.95397	6448.844	12787.42	2129.953
1992	Male	other and unspecified causes	5.671655	2594.097	5293.888	774.629
1992	Female	other and unspecified causes	5.577559	3008.943	5862.782	943.6276
1991	Male	diabetes mellitus type 2	42.99121	18863.14	32945.08	7910.59
1991	Female	diabetes mellitus type 2	44.97248	23239.24	37675.1	10198.82
1991	Male	hypertension	42.23162	18529.85	31459.52	8417.45
1991	Female	hypertension	37.41142	19332.11	29811.17	9501.656
1991	Male	glomerulonephritis	9.096127	3991.083	8208.181	1262.967
1991	Female	glomerulonephritis	12.03874	6220.943	12292.82	2039.962
1991	Male	other and unspecified causes	5.681047	2492.657	5083.313	741.3133
1991	Female	other and unspecified causes	5.577354	2882.062	5582.222	908.1389
1990	Male	diabetes mellitus type 2	42.97673	18140.9	31336.32	7570.38
1990	Female	diabetes mellitus type 2	44.87695	22337.6	35884.28	9884.126
1990	Male	hypertension	42.17987	17804.54	30385.89	8037.144
1990	Female	hypertension	37.51218	18671.77	28969.76	9115.037
1990	Male	glomerulonephritis	9.156814	3865.182	7918.94	1224.751
1990	Female	glomerulonephritis	12.05179	5998.803	11867.82	1946.803
1990	Male	other and unspecified causes	5.686584	2400.364	4900.895	712.144
1990	Female	other and unspecified causes	5.559088	2767.048	5405.874	879.9663

Table S8

The high BMI-attributable CKD DALY causes for both sexes and all age groups in global from 1990 to 2021

year	sex	cause	proportion	val	upper	lower
2021	Male	diabetes mellitus type 2	40.87043	2069470	3375308	905007.3
2021	Female	diabetes mellitus type 2	42.05219	2253607	3491730	1048253
2021	Male	glomerulonephritis	6.166713	312250.8	630038.7	93579.69
2021	Female	glomerulonephritis	6.327585	339099.8	667860.9	111920.2
2021	Male	hypertension	43.70903	2213202	3225398	1082658
2021	Female	hypertension	38.13785	2043835	2932756	1032283
2021	Male	other and unspecified causes	9.253827	468566.6	932991.5	150717.2
2021	Female	other and unspecified causes	13.48237	722530	1368297	252517.8
2020	Male	diabetes mellitus type 2	40.73639	1997805	3295626	891640
2020	Female	diabetes mellitus type 2	41.76297	2149280	3321133	970229.6
2020	Male	hypertension	43.84021	2150023	3129771	1039364
2020	Female	hypertension	38.39406	1975903	2820262	1002725
2020	Male	glomerulonephritis	6.163064	302250.6	605489.3	91829.26
2020	Female	glomerulonephritis	6.309062	324688.1	636041.9	106195.7
2020	Male	other and unspecified causes	9.260336	454147.9	892764.4	147380
2020	Female	other and unspecified causes	13.53391	696506.2	1321740	243113.9
2019	Male	diabetes mellitus type 2	40.64852	1918905	3158657	822382.9
2019	Female	diabetes mellitus type 2	41.92438	2072876	3213379	966362.4
2019	Male	hypertension	43.96468	2075451	3048477	997327.8
2019	Female	hypertension	38.42884	1900045	2717661	960196
2019	Male	glomerulonephritis	6.147217	290193.2	583827	88478.32
2019	Female	glomerulonephritis	6.197805	306439.4	598879.6	97866.47
2019	Male	other and unspecified causes	9.239592	436175.7	865325.1	141711.2
2019	Female	other and unspecified causes	13.44898	664960.5	1263506	231463.6
2018	Male	diabetes mellitus type 2	40.72627	1848765	3037587	808459
2018	Female	diabetes mellitus type 2	41.91887	1994003	3108696	922525.6
2018	Male	hypertension	43.91804	1993655	2963016	958592.9
2018	Female	hypertension	38.42997	1828043	2592743	926533.5
2018	Male	glomerulonephritis	6.146635	279025.9	557935.7	84436.89
2018	Female	glomerulonephritis	6.215989	295683.1	572742.4	94462.57
2018	Male	other and unspecified causes	9.20906	418044.4	833568.4	135071.2
2018	Female	other and unspecified causes	13.43516	639086	1213012	222496.1
2017	Male	diabetes mellitus type 2	40.82787	1797295	2997660	789438.9
2017	Female	diabetes mellitus type 2	41.89992	1931735	2952898	895095.9
2017	Male	hypertension	43.80748	1928461	2823214	937825.1
2017	Female	hypertension	38.43962	1772203	2508217	898459.2
2017	Male	glomerulonephritis	6.183874	272222.1	548138.4	82095.4
2017	Female	glomerulonephritis	6.264842	288831.5	565614.3	92376.18
2017	Male	other and unspecified causes	9.180774	404149.4	807382.2	129364.2

2017	Female	other and unspecified causes	13.39562	617585.5	1173411	213414.9
2016	Male	diabetes mellitus type 2	40.93559	1745932	2852168	776121.1
2016	Female	diabetes mellitus type 2	41.90373	1869438	2890001	855305.4
2016	Male	hypertension	43.74912	1865931	2739731	909602.1
2016	Female	hypertension	38.4747	1716460	2431410	872018.8
2016	Male	glomerulonephritis	6.176603	263436.5	531943.1	78995.58
2016	Female	glomerulonephritis	6.258042	279188.1	546078.7	89273.21
2016	Male	other and unspecified causes	9.138682	389771.3	777843.7	123837.4
2016	Female	other and unspecified causes	13.36352	596182.7	1142810	204538.2
2015	Male	diabetes mellitus type 2	41.0832	1674510	2733612	725672.5
2015	Female	diabetes mellitus type 2	42.00176	1790733	2758516	809613.3
2015	Male	hypertension	43.70985	1781569	2627130	873105.7
2015	Female	hypertension	38.48075	1640616	2326618	833740.1
2015	Male	glomerulonephritis	6.110612	249062.4	505983.2	73566.53
2015	Female	glomerulonephritis	6.186591	263763.6	518708.5	83482.07
2015	Male	other and unspecified causes	9.096338	370757.6	745078	117631.4
2015	Female	other and unspecified causes	13.3309	568359.4	1090695	193019.3
2014	Male	diabetes mellitus type 2	41.15536	1595747	2618424	698574
2014	Female	diabetes mellitus type 2	42.11457	1708081	2599289	777135
2014	Male	hypertension	43.68301	1693753	2521511	821659.7
2014	Female	hypertension	38.4935	1561218	2221166	782849.6
2014	Male	glomerulonephritis	6.072392	235449.3	479541.8	70051.59
2014	Female	glomerulonephritis	6.12287	248331.1	488531.7	78069.15
2014	Male	other and unspecified causes	9.08924	352423.7	711367.4	110183.9
2014	Female	other and unspecified causes	13.26906	538165.8	1033787	182199.9
2013	Male	diabetes mellitus type 2	41.17539	1528901	2505628	665821.5
2013	Female	diabetes mellitus type 2	42.17126	1636137	2512803	745939.6
2013	Male	hypertension	43.6267	1619921	2404994	787504
2013	Female	hypertension	38.46075	1492179	2139388	744949.7
2013	Male	glomerulonephritis	6.080535	225778.9	461864.6	67558.3
2013	Female	glomerulonephritis	6.112183	237137.1	470659.6	74421.78
2013	Male	other and unspecified causes	9.117376	338541.1	685867.1	105571.2
2013	Female	other and unspecified causes	13.25581	514291.6	992066.4	173611.8
2012	Male	diabetes mellitus type 2	41.18265	1451689	2368812	632563.2
2012	Female	diabetes mellitus type 2	42.23793	1565685	2417373	715717.9
2012	Male	hypertension	43.55067	1535161	2282172	740582.1
2012	Female	hypertension	38.39796	1423344	2028592	710039.4
2012	Male	glomerulonephritis	6.066993	213861.5	440570.6	63617.93
2012	Female	glomerulonephritis	6.071408	225056.3	445631.6	70362.4
2012	Male	other and unspecified causes	9.199679	324288.7	661893	100922.4
2012	Female	other and unspecified causes	13.29271	492737.1	958622.7	165981.3
2011	Male	diabetes mellitus type 2	41.18978	1387182	2272016	604944
2011	Female	diabetes mellitus type 2	42.35151	1503457	2322742	698304.9
2011	Male	hypertension	43.47211	1464046	2181898	702054.8

2011	Female	hypertension	38.33504	1360875	1945475	678225.6
2011	Male	glomerulonephritis	6.076992	204659.9	423183.1	60895.86
2011	Female	glomerulonephritis	6.034419	214218.8	429037.1	66338.86
2011	Male	other and unspecified causes	9.261109	311894	636858.1	97002.77
2011	Female	other and unspecified causes	13.27903	471398.9	918777.5	159491.4
2010	Male	diabetes mellitus type 2	41.18639	1317859	2171878	571893.9
2010	Female	diabetes mellitus type 2	42.32767	1436814	2228608	666828.5
2010	Male	hypertension	43.32416	1386262	2074714	662624.9
2010	Female	hypertension	38.26982	1299070	1851635	641969.2
2010	Male	glomerulonephritis	6.141291	196505.6	405499.2	57932.54
2010	Female	glomerulonephritis	6.06609	205913.5	411345.2	63355.69
2010	Male	other and unspecified causes	9.348167	299117.4	611123.4	93254.93
2010	Female	other and unspecified causes	13.33642	452705	882284.5	152607.5
2009	Male	diabetes mellitus type 2	41.2181	1258849	2060874	550804.2
2009	Female	diabetes mellitus type 2	42.35813	1378606	2138221	642522.7
2009	Male	hypertension	43.21729	1319907	1974237	634416.1
2009	Female	hypertension	38.1859	1242815	1784328	614385.2
2009	Male	glomerulonephritis	6.1754	188603.9	390603.2	55639.59
2009	Female	glomerulonephritis	6.097192	198441.9	397887	61571.13
2009	Male	other and unspecified causes	9.389205	286757.3	584237	89617.2
2009	Female	other and unspecified causes	13.35878	434780.5	848261.3	147504.7
2008	Male	diabetes mellitus type 2	41.26091	1202925	1981413	520723.9
2008	Female	diabetes mellitus type 2	42.52732	1324262	2042323	612040.5
2008	Male	hypertension	43.08718	1256169	1897827	604245.6
2008	Female	hypertension	38.04403	1184656	1705964	584649.3
2008	Male	glomerulonephritis	6.207002	180959.7	375915.9	53429.27
2008	Female	glomerulonephritis	6.071485	189060.5	380583.4	58065.74
2008	Male	other and unspecified causes	9.444902	275357.8	562727.5	86158.47
2008	Female	other and unspecified causes	13.35716	415929.7	815483.5	140760.2
2007	Male	diabetes mellitus type 2	41.3754	1145407	1885672	500800.2
2007	Female	diabetes mellitus type 2	42.7348	1271916	1971580	583388.3
2007	Male	hypertension	43.02308	1191021	1807681	574075.9
2007	Female	hypertension	37.91481	1128459	1627994	554278.6
2007	Male	glomerulonephritis	6.142906	170055.9	354183.1	50021.95
2007	Female	glomerulonephritis	6.000199	178584	359603.9	54079.17
2007	Male	other and unspecified causes	9.458617	261845.7	536053.2	82294.27
2007	Female	other and unspecified causes	13.35019	397342	780147.6	134103.6
2006	Male	diabetes mellitus type 2	41.53105	1098260	1800423	479645.2
2006	Female	diabetes mellitus type 2	42.96083	1227673	1912870	568544.6
2006	Male	hypertension	42.9529	1135860	1728896	540980.8
2006	Female	hypertension	37.80952	1080466	1564697	530953.8
2006	Male	glomerulonephritis	6.084879	160910.5	336118	46988.44
2006	Female	glomerulonephritis	5.930008	169459.3	339376.9	50792.17
2006	Male	other and unspecified causes	9.431177	249401	511265.5	78590.27

2006	Female	other and unspecified causes	13.29964	380058.3	749117.8	127665.6
2005	Male	diabetes mellitus type 2	41.77424	1061475	1740160	463035.1
2005	Female	diabetes mellitus type 2	43.22385	1191902	1857193	549085
2005	Male	hypertension	42.7935	1087374	1665709	518933.1
2005	Female	hypertension	37.63525	1037796	1507537	504330.7
2005	Male	glomerulonephritis	6.03813	153427.6	319511.2	44631.41
2005	Female	glomerulonephritis	5.896273	162590.3	327341.3	48308.96
2005	Male	other and unspecified causes	9.394128	238702.9	491694.1	75649.59
2005	Female	other and unspecified causes	13.24462	365221.7	720463.3	121669
2004	Male	diabetes mellitus type 2	41.91766	1019385	1679850	442886.8
2004	Female	diabetes mellitus type 2	43.37575	1148969	1774579	532962.1
2004	Male	hypertension	42.74386	1039477	1596348	491465.6
2004	Female	hypertension	37.56349	995009.4	1461936	484502.2
2004	Male	glomerulonephritis	6.002783	145980.2	304941.8	42288.19
2004	Female	glomerulonephritis	5.868267	155443	313168.4	46321.48
2004	Male	other and unspecified causes	9.335694	227032.4	470913.4	71232.41
2004	Female	other and unspecified causes	13.19249	349452.5	690246.2	116150.5
2003	Male	diabetes mellitus type 2	41.84703	974156.1	1594893	427445.6
2003	Female	diabetes mellitus type 2	43.35364	1104572	1703781	512487.3
2003	Male	hypertension	42.7306	994724.8	1542139	467028.3
2003	Female	hypertension	37.46185	954459.8	1394471	461606.2
2003	Male	glomerulonephritis	6.059302	141054.4	295390.3	40327.12
2003	Female	glomerulonephritis	5.927022	151009.8	304922.5	45501.91
2003	Male	other and unspecified causes	9.363076	217962.9	452247.8	68456.77
2003	Female	other and unspecified causes	13.25749	337776.7	665978.7	112465.7
2002	Male	diabetes mellitus type 2	41.8236	922833.8	1517524	407949.3
2002	Female	diabetes mellitus type 2	43.38099	1054594	1627378	486822.4
2002	Male	hypertension	42.71048	942402.5	1456670	443188.8
2002	Female	hypertension	37.33748	907676	1331261	440941
2002	Male	glomerulonephritis	6.071113	133958.5	279925.8	38314.31
2002	Female	glomerulonephritis	5.943121	144477.6	291502.9	43967.94
2002	Male	other and unspecified causes	9.394808	207295.5	430652.7	65331.4
2002	Female	other and unspecified causes	13.33841	324257.5	640333.3	107736.7
2001	Male	diabetes mellitus type 2	41.83306	879226.2	1427350	390654.8
2001	Female	diabetes mellitus type 2	43.46271	1007401	1549130	472041.9
2001	Male	hypertension	42.6482	896358.5	1407620	419261.6
2001	Female	hypertension	37.17207	861592.9	1261941	418117
2001	Male	glomerulonephritis	6.097978	128164.3	269535.8	36369.08
2001	Female	glomerulonephritis	5.966701	138299.2	279758.3	42523.39
2001	Male	other and unspecified causes	9.420759	198000.8	411871.9	62385.67
2001	Female	other and unspecified causes	13.39851	310557.5	613446.9	101706.9
2000	Male	diabetes mellitus type 2	41.91466	838229.4	1356703	372110.3
2000	Female	diabetes mellitus type 2	43.58306	960801.1	1494571	450452.7
2000	Male	hypertension	42.51242	850183.5	1332407	397555.6

2000	Female	hypertension	36.96121	814820.5	1195899	396766.8
2000	Male	glomerulonephritis	6.121479	122420.2	258483.8	34503.25
2000	Female	glomerulonephritis	5.992597	132108.5	267614.4	40671.13
2000	Male	other and unspecified causes	9.451437	189014.3	395227.5	59678.02
2000	Female	other and unspecified causes	13.46314	296798.8	584907.1	95745.55
1999	Male	diabetes mellitus type 2	42.15433	794299.3	1297359	352933
1999	Female	diabetes mellitus type 2	43.82518	911322.7	1403980	425157.9
1999	Male	hypertension	42.2924	796901	1272519	372313.5
1999	Female	hypertension	36.68254	762795.1	1121948	373167
1999	Male	glomerulonephritis	6.083022	114620.3	241064.4	32780.02
1999	Female	glomerulonephritis	5.969267	124127.9	253047.1	38102.14
1999	Male	other and unspecified causes	9.47025	178444.6	373959.5	55820.32
1999	Female	other and unspecified causes	13.52302	281204.4	554421	90308.79
1998	Male	diabetes mellitus type 2	42.44192	754861.9	1239383	333831.3
1998	Female	diabetes mellitus type 2	44.05016	863784.1	1346992	404837.3
1998	Male	hypertension	42.08008	748426.3	1193763	348092.4
1998	Female	hypertension	36.49593	715652.5	1059854	346757.3
1998	Male	glomerulonephritis	6.028819	107227.1	225456.8	30849.67
1998	Female	glomerulonephritis	5.915037	115988.6	236279.9	35495.26
1998	Male	other and unspecified causes	9.44918	168060.9	353654.2	52196.34
1998	Female	other and unspecified causes	13.53887	265485.2	522635.7	84423.66
1997	Male	diabetes mellitus type 2	42.76375	716990.5	1179669	316036.3
1997	Female	diabetes mellitus type 2	44.43918	819931.9	1261122	380680.9
1997	Male	hypertension	41.9158	702773.5	1122339	326877.6
1997	Female	hypertension	36.32121	670150.1	999952.8	325197.6
1997	Male	glomerulonephritis	5.923159	99309.54	210923.8	28648.31
1997	Female	glomerulonephritis	5.780071	106646.1	219206.8	32067.44
1997	Male	other and unspecified causes	9.397286	157557.8	331951.1	48398.35
1997	Female	other and unspecified causes	13.45954	248337.3	489979.4	77859.08
1996	Male	diabetes mellitus type 2	43.00066	690207.5	1147177	301799.2
1996	Female	diabetes mellitus type 2	44.76592	788309.1	1222168	361404.7
1996	Male	hypertension	41.66974	668844.7	1083154	314273.3
1996	Female	hypertension	36.04946	634815.9	947222.7	307788
1996	Male	glomerulonephritis	5.937233	95299.06	201849.2	27273.77
1996	Female	glomerulonephritis	5.771892	101640.6	208589.6	30336.94
1996	Male	other and unspecified causes	9.39236	150757.6	317339.9	45731.88
1996	Female	other and unspecified causes	13.41273	236192.6	466853.2	72983.8
1995	Male	diabetes mellitus type 2	43.17428	665322.5	1092756	292798.7
1995	Female	diabetes mellitus type 2	44.96948	760013.9	1175199	349025.4
1995	Male	hypertension	41.44598	638689.2	1041489	296703.9
1995	Female	hypertension	35.81067	605224	914003.8	291463.5
1995	Male	glomerulonephritis	5.974663	92070.5	194382.8	26012.96
1995	Female	glomerulonephritis	5.802668	98068.92	201490.1	29157.05
1995	Male	other and unspecified causes	9.405073	144933.7	306115.1	43122.16

1995	Female	other and unspecified causes	13.41718	226759.2	447546.2	69439.21
1994	Male	diabetes mellitus type 2	43.31394	639421.9	1056196	281024
1994	Female	diabetes mellitus type 2	45.12713	734512.6	1136495	343561.5
1994	Male	hypertension	41.30267	609730.5	995879.7	284305.7
1994	Female	hypertension	35.67091	580598.1	873021.8	278350.4
1994	Male	glomerulonephritis	6.009672	88717.77	187627.8	24880.6
1994	Female	glomerulonephritis	5.824444	94801.67	194292.3	28060.92
1994	Male	other and unspecified causes	9.373724	138379.6	292875.7	40884.52
1994	Female	other and unspecified causes	13.37752	217739.4	431141.1	66185.51
1993	Male	diabetes mellitus type 2	43.53641	618785.9	1024261	272274.8
1993	Female	diabetes mellitus type 2	45.33255	710477.5	1108003	327686
1993	Male	hypertension	41.09943	584149.1	956360	271562.9
1993	Female	hypertension	35.51316	556582.5	848639.6	266461.1
1993	Male	glomerulonephritis	5.974692	84918.71	180456	23658.82
1993	Female	glomerulonephritis	5.785971	90681.03	185958.2	26743.58
1993	Male	other and unspecified causes	9.389467	133453.1	282616.8	38979.97
1993	Female	other and unspecified causes	13.36832	209516	416513.8	63461.26
1992	Male	diabetes mellitus type 2	43.75455	597221.1	985501.2	262316.9
1992	Female	diabetes mellitus type 2	45.45351	685711.3	1066832	316001.4
1992	Male	hypertension	40.86855	557829.2	913175.3	258940.4
1992	Female	hypertension	35.36762	533555.6	807880.5	257240.7
1992	Male	glomerulonephritis	5.94001	81077.27	172527.1	22644.86
1992	Female	glomerulonephritis	5.764157	86958.02	178571	25776.45
1992	Male	other and unspecified causes	9.436887	128807.4	272905.7	37264.98
1992	Female	other and unspecified causes	13.41471	202374.2	402984.3	60507.68
1991	Male	diabetes mellitus type 2	43.92653	576856.1	963405.5	251775.2
1991	Female	diabetes mellitus type 2	45.58729	661699.2	1026543	297271.2
1991	Male	hypertension	40.66403	534011.9	879814.3	247303.2
1991	Female	hypertension	35.22919	511351.5	784747.1	245780.8
1991	Male	glomerulonephritis	5.918757	77726.85	165284.1	21664.58
1991	Female	glomerulonephritis	5.73892	83300.39	171166.4	24567.25
1991	Male	other and unspecified causes	9.490684	124634.4	264101.1	35740.55
1991	Female	other and unspecified causes	13.4446	195148.3	389604.8	57681.76
1990	Male	diabetes mellitus type 2	44.00495	557967.4	925038	242064.5
1990	Female	diabetes mellitus type 2	45.64553	640005	1002393	291542.6
1990	Male	hypertension	40.57878	514524.7	864141.3	235950.9
1990	Female	hypertension	35.22149	493847.5	757913.8	234468.8
1990	Male	glomerulonephritis	5.9014	74827.68	158840.9	20586.55
1990	Female	glomerulonephritis	5.714512	80124.3	163966.1	23494.27
1990	Male	other and unspecified causes	9.514862	120645.1	255893	34602.16
1990	Female	other and unspecified causes	13.41847	188143.1	376423.5	54908.53

Table S9

Correlation between ASMR/ASDR for high BMI-attributable CKD with SDI in the 21 GBD regions from 1990 to 2021.

location	Year	ASMR	ASDR	SDI value
Andean Latin America	1990	8.19638	186.821	0.500011
Andean Latin America	1991	8.226956	187.6757	0.501652
Andean Latin America	1992	8.668337	196.3719	0.503656
Andean Latin America	1993	8.933883	201.2749	0.506388
Andean Latin America	1994	9.510403	213.5848	0.510306
Andean Latin America	1995	9.914727	220.8334	0.514896
Andean Latin America	1996	10.18284	223.4527	0.519376
Andean Latin America	1997	10.65369	231.1143	0.524036
Andean Latin America	1998	11.23317	242.7988	0.528444
Andean Latin America	1999	11.34861	246.3139	0.532744
Andean Latin America	2000	12.13998	262.4216	0.537561
Andean Latin America	2001	12.6302	272.7169	0.542572
Andean Latin America	2002	13.24371	285.8516	0.547911
Andean Latin America	2003	13.6394	294.3073	0.553109
Andean Latin America	2004	13.55099	292.7937	0.558235
Andean Latin America	2005	13.77567	299.3405	0.563092
Andean Latin America	2006	13.8249	301.2035	0.567987
Andean Latin America	2007	14.01405	305.7482	0.572999
Andean Latin America	2008	14.23362	311.8258	0.578564
Andean Latin America	2009	14.82523	323.407	0.583999
Andean Latin America	2010	14.8091	320.2948	0.590201
Andean Latin America	2011	14.79843	317.6622	0.596945
Andean Latin America	2012	14.97624	320.0915	0.603839
Andean Latin America	2013	14.73385	313.6929	0.610705
Andean Latin America	2014	14.08344	299.7647	0.61706
Andean Latin America	2015	13.81518	294.7696	0.622761
Andean Latin America	2016	13.92153	296.8672	0.628161
Andean Latin America	2017	14.33519	305.4779	0.633538
Andean Latin America	2018	14.54195	309.6779	0.63886
Andean Latin America	2019	15.03713	319.4809	0.643921
Andean Latin America	2020	14.79623	320.4885	0.647807
Andean Latin America	2021	14.71654	320.22	0.651602
Australasia	1990	2.335121	53.97281	0.731234
Australasia	1991	2.299085	53.96473	0.734915
Australasia	1992	2.31067	54.60595	0.739006
Australasia	1993	2.368315	55.86665	0.743426
Australasia	1994	2.350644	55.45144	0.747726
Australasia	1995	2.376954	55.88523	0.752015
Australasia	1996	2.424849	56.79087	0.756387

Australasia	1997	2.393003	57.21426	0.76099
Australasia	1998	2.511348	58.80732	0.7653
Australasia	1999	2.553884	59.45855	0.769521
Australasia	2000	2.778077	63.46679	0.773791
Australasia	2001	2.83951	64.57419	0.778074
Australasia	2002	2.925198	65.6697	0.78248
Australasia	2003	2.917442	65.86587	0.786285
Australasia	2004	2.926599	66.14898	0.789428
Australasia	2005	2.996396	67.18033	0.791722
Australasia	2006	3.167377	69.47156	0.792857
Australasia	2007	3.415449	72.35198	0.79395
Australasia	2008	3.540207	73.70114	0.796402
Australasia	2009	3.580421	73.87192	0.799932
Australasia	2010	3.487476	72.18972	0.804112
Australasia	2011	3.517057	72.994	0.808161
Australasia	2012	3.638049	75.14279	0.812464
Australasia	2013	3.955743	80.61584	0.817349
Australasia	2014	4.082999	82.73006	0.821647
Australasia	2015	4.174617	84.59076	0.825611
Australasia	2016	4.092248	82.95414	0.829457
Australasia	2017	4.021815	81.90318	0.832924
Australasia	2018	3.693566	76.5725	0.836619
Australasia	2019	3.725336	77.22225	0.840582
Australasia	2020	3.505326	73.77117	0.843333
Australasia	2021	3.639655	76.15667	0.845514
Caribbean	1990	4.910634	126.2921	0.518111
Caribbean	1991	4.824498	125.0403	0.522789
Caribbean	1992	4.794484	124.8643	0.526984
Caribbean	1993	4.823501	125.9598	0.530507
Caribbean	1994	4.498903	119.9608	0.533584
Caribbean	1995	4.504178	120.646	0.536651
Caribbean	1996	4.679533	124.585	0.539776
Caribbean	1997	4.788785	127.7566	0.543303
Caribbean	1998	4.99536	133.1263	0.547365
Caribbean	1999	5.286774	140.0129	0.55206
Caribbean	2000	5.658629	148.1378	0.557369
Caribbean	2001	6.050443	158.7578	0.56311
Caribbean	2002	5.920319	156.7824	0.569069
Caribbean	2003	6.118867	161.3586	0.574889
Caribbean	2004	6.273017	164.0689	0.580533
Caribbean	2005	6.442883	168.1752	0.585758
Caribbean	2006	6.722362	174.268	0.590602
Caribbean	2007	6.803554	177.0913	0.59471
Caribbean	2008	6.842856	178.8342	0.598297

Caribbean	2009	7.179737	186.7858	0.601655
Caribbean	2010	7.401268	192.1065	0.60548
Caribbean	2011	7.777964	200.5952	0.609525
Caribbean	2012	7.974938	205.437	0.613255
Caribbean	2013	8.321876	214.301	0.616877
Caribbean	2014	8.471482	218.5944	0.620443
Caribbean	2015	8.558877	221.5343	0.624075
Caribbean	2016	8.817263	226.8727	0.627582
Caribbean	2017	9.00672	231.109	0.630788
Caribbean	2018	9.041964	232.1347	0.633972
Caribbean	2019	9.21287	235.2326	0.637321
Caribbean	2020	9.318086	235.6671	0.639709
Caribbean	2021	9.328114	234.8101	0.642003
Central Asia	1990	0.995445	68.67966	0.553362
Central Asia	1991	1.230588	75.03749	0.555148
Central Asia	1992	1.384311	79.72777	0.55727
Central Asia	1993	1.512942	83.83144	0.560083
Central Asia	1994	1.577053	86.16193	0.5627
Central Asia	1995	1.627239	88.08996	0.565586
Central Asia	1996	1.702358	90.44519	0.568761
Central Asia	1997	1.728298	91.17651	0.571766
Central Asia	1998	1.820407	93.8107	0.574659
Central Asia	1999	1.964994	97.5698	0.578121
Central Asia	2000	1.967857	98.23427	0.582156
Central Asia	2001	2.059714	101.0717	0.586646
Central Asia	2002	2.156166	103.8581	0.591457
Central Asia	2003	2.344024	108.6287	0.59653
Central Asia	2004	2.686451	117.089	0.601737
Central Asia	2005	2.858475	121.2847	0.607294
Central Asia	2006	2.827384	120.5674	0.613078
Central Asia	2007	2.886329	121.9795	0.619105
Central Asia	2008	2.905308	122.4809	0.62493
Central Asia	2009	3.013218	125.23	0.630103
Central Asia	2010	3.059359	126.0714	0.635296
Central Asia	2011	3.097359	126.8258	0.640314
Central Asia	2012	2.931379	122.6079	0.644674
Central Asia	2013	2.792246	119.1677	0.648888
Central Asia	2014	2.861807	120.6449	0.653
Central Asia	2015	2.951022	122.3583	0.656818
Central Asia	2016	3.243311	129.467	0.660312
Central Asia	2017	3.246361	129.3255	0.663577
Central Asia	2018	3.156448	127.6313	0.666666
Central Asia	2019	3.189839	128.707	0.669837
Central Asia	2020	3.177163	128.2111	0.672487

Central Asia	2021	3.138427	127.6971	0.675164
Central Europe	1990	3.060304	88.99615	0.637272
Central Europe	1991	3.055185	88.6706	0.643083
Central Europe	1992	3.010007	87.54638	0.648772
Central Europe	1993	2.872794	84.50662	0.65447
Central Europe	1994	2.872758	84.49346	0.661219
Central Europe	1995	2.925098	85.66996	0.667955
Central Europe	1996	2.915358	85.28839	0.674121
Central Europe	1997	2.934447	85.52206	0.680037
Central Europe	1998	2.932329	84.86514	0.685968
Central Europe	1999	2.982698	85.74013	0.692056
Central Europe	2000	3.03251	85.97182	0.69878
Central Europe	2001	3.037808	85.83042	0.705657
Central Europe	2002	3.079562	86.36355	0.712001
Central Europe	2003	3.16008	87.78908	0.717824
Central Europe	2004	3.197442	88.13118	0.723494
Central Europe	2005	3.358986	91.49179	0.728674
Central Europe	2006	3.427002	93.01101	0.733476
Central Europe	2007	3.473541	93.994	0.738077
Central Europe	2008	3.472479	94.16358	0.743148
Central Europe	2009	3.503348	94.71397	0.748766
Central Europe	2010	3.435678	93.46057	0.754919
Central Europe	2011	3.417513	93.12306	0.760349
Central Europe	2012	3.287471	90.89849	0.764803
Central Europe	2013	3.105159	87.62604	0.768568
Central Europe	2014	2.993755	85.83559	0.771755
Central Europe	2015	3.075933	87.21273	0.774844
Central Europe	2016	3.133865	88.00655	0.777817
Central Europe	2017	3.22706	89.50008	0.781359
Central Europe	2018	3.25709	89.70311	0.785476
Central Europe	2019	3.256593	89.60336	0.789749
Central Europe	2020	3.314119	89.96141	0.793111
Central Europe	2021	3.299393	89.32002	0.796244
Central Latin America	1990	7.342729	186.213	0.485787
Central Latin America	1991	7.50332	191.3126	0.489559
Central Latin America	1992	7.605504	194.887	0.49411
Central Latin America	1993	7.60408	196.3628	0.499418
Central Latin America	1994	7.679536	198.0483	0.505061
Central Latin America	1995	7.952909	204.0154	0.509874
Central Latin America	1996	8.276797	210.8938	0.514693
Central Latin America	1997	8.789235	224.3431	0.520171
Central Latin America	1998	10.51878	268.6463	0.526132
Central Latin America	1999	11.27991	288.3455	0.531807
Central Latin America	2000	12.1457	309.0118	0.5373

Central Latin America	2001	12.5538	318.7225	0.54244
Central Latin America	2002	12.73587	323.6837	0.547134
Central Latin America	2003	13.20413	335.8916	0.551446
Central Latin America	2004	13.3844	339.8782	0.556011
Central Latin America	2005	13.77459	349.7817	0.56075
Central Latin America	2006	14.44291	366.1128	0.565649
Central Latin America	2007	15.18774	384.7953	0.570707
Central Latin America	2008	16.01984	405.3955	0.575839
Central Latin America	2009	16.16922	408.9275	0.57994
Central Latin America	2010	15.94062	403.0746	0.584253
Central Latin America	2011	15.73108	398.2654	0.589308
Central Latin America	2012	15.87698	402.4059	0.594827
Central Latin America	2013	16.18964	409.8334	0.600731
Central Latin America	2014	15.90582	402.7056	0.606509
Central Latin America	2015	16.19439	410.2423	0.611815
Central Latin America	2016	16.46582	418.7084	0.617086
Central Latin America	2017	15.90194	405.4287	0.622442
Central Latin America	2018	14.76581	378.2644	0.627765
Central Latin America	2019	14.48679	371.6846	0.632847
Central Latin America	2020	14.75946	384.5348	0.636996
Central Latin America	2021	15.0064	391.2414	0.640685
Central Sub-Saharan Africa	1990	5.363729	132.2027	0.302375
Central Sub-Saharan Africa	1991	5.451946	134.6723	0.305221
Central Sub-Saharan Africa	1992	5.51568	136.2684	0.307804
Central Sub-Saharan Africa	1993	5.588278	137.9839	0.309082
Central Sub-Saharan Africa	1994	5.710505	140.845	0.309898
Central Sub-Saharan Africa	1995	5.842837	144.1627	0.311059
Central Sub-Saharan Africa	1996	6.028672	148.5541	0.312674
Central Sub-Saharan Africa	1997	6.009481	147.8339	0.314502
Central Sub-Saharan Africa	1998	6.116624	150.4345	0.316506
Central Sub-Saharan Africa	1999	6.195126	152.4832	0.318569
Central Sub-Saharan Africa	2000	6.279144	154.5002	0.320841
Central Sub-Saharan Africa	2001	6.309675	155.2211	0.323749
Central Sub-Saharan Africa	2002	6.286104	154.6215	0.327567
Central Sub-Saharan Africa	2003	6.389084	157.3314	0.331935
Central Sub-Saharan Africa	2004	6.401152	157.5869	0.33742
Central Sub-Saharan Africa	2005	6.396934	157.4534	0.344099
Central Sub-Saharan Africa	2006	6.457432	158.8314	0.35146
Central Sub-Saharan Africa	2007	6.505067	159.9411	0.359566
Central Sub-Saharan Africa	2008	6.63279	162.7781	0.368488
Central Sub-Saharan Africa	2009	6.744397	165.3051	0.376669
Central Sub-Saharan Africa	2010	6.870449	168.1076	0.385381
Central Sub-Saharan Africa	2011	7.038566	171.7997	0.394692
Central Sub-Saharan Africa	2012	7.211883	175.5898	0.404234

Central Sub-Saharan Africa	2013	7.40507	179.6531	0.413522
Central Sub-Saharan Africa	2014	7.593741	183.6681	0.422579
Central Sub-Saharan Africa	2015	7.781375	187.4812	0.430988
Central Sub-Saharan Africa	2016	7.981254	191.8625	0.438746
Central Sub-Saharan Africa	2017	8.203683	196.746	0.446179
Central Sub-Saharan Africa	2018	8.431734	201.7857	0.45325
Central Sub-Saharan Africa	2019	8.651651	206.3895	0.46016
Central Sub-Saharan Africa	2020	8.941134	212.6092	0.466456
Central Sub-Saharan Africa	2021	9.190197	218.6653	0.472256
East Asia	1990	1.885399	44.83943	0.471179
East Asia	1991	1.920041	45.22973	0.479936
East Asia	1992	1.94281	45.46162	0.488431
East Asia	1993	1.959487	45.57355	0.496859
East Asia	1994	1.974883	45.72726	0.504886
East Asia	1995	1.978289	45.79094	0.51353
East Asia	1996	1.963641	45.64712	0.523758
East Asia	1997	1.949751	45.54373	0.533816
East Asia	1998	1.954675	45.91775	0.542653
East Asia	1999	1.969924	46.5549	0.551367
East Asia	2000	2.034647	47.92118	0.558816
East Asia	2001	2.097003	49.52755	0.564235
East Asia	2002	2.162263	51.62339	0.570798
East Asia	2003	2.279526	54.46618	0.578905
East Asia	2004	2.36933	56.95552	0.587683
East Asia	2005	2.383624	57.6433	0.597096
East Asia	2006	2.288806	56.26189	0.607273
East Asia	2007	2.280928	56.06215	0.617524
East Asia	2008	2.328242	56.7234	0.626956
East Asia	2009	2.393639	57.58986	0.636271
East Asia	2010	2.466513	58.64077	0.647985
East Asia	2011	2.493617	59.1673	0.657502
East Asia	2012	2.486562	59.37672	0.66293
East Asia	2013	2.522392	59.95382	0.668682
East Asia	2014	2.54395	60.44095	0.674063
East Asia	2015	2.552639	60.87604	0.677307
East Asia	2016	2.600379	61.71365	0.681528
East Asia	2017	2.651654	62.5266	0.689748
East Asia	2018	2.698058	63.43847	0.698626
East Asia	2019	2.737293	64.23362	0.708328
East Asia	2020	2.789466	65.43494	0.717709
East Asia	2021	2.913817	68.23699	0.725705
Eastern Europe	1990	0.646226	38.90806	0.66425
Eastern Europe	1991	0.673379	39.89102	0.671452
Eastern Europe	1992	0.702887	41.10358	0.678753

Eastern Europe	1993	0.781452	43.88814	0.683359
Eastern Europe	1994	0.845228	46.1382	0.686012
Eastern Europe	1995	0.825494	45.46704	0.689459
Eastern Europe	1996	0.781964	44.15657	0.692791
Eastern Europe	1997	0.73368	42.74623	0.695203
Eastern Europe	1998	0.73131	42.65373	0.69694
Eastern Europe	1999	0.793968	44.44776	0.698814
Eastern Europe	2000	0.829757	45.32298	0.701011
Eastern Europe	2001	0.834384	45.23396	0.703506
Eastern Europe	2002	0.87087	46.16112	0.706727
Eastern Europe	2003	0.884183	46.62697	0.712025
Eastern Europe	2004	0.846095	45.92388	0.719215
Eastern Europe	2005	0.897881	47.48603	0.726266
Eastern Europe	2006	0.875835	46.80655	0.732617
Eastern Europe	2007	0.879484	46.78934	0.738787
Eastern Europe	2008	0.914946	47.52805	0.74545
Eastern Europe	2009	0.911585	47.17396	0.75107
Eastern Europe	2010	0.95816	47.78034	0.756606
Eastern Europe	2011	0.966032	47.36292	0.761025
Eastern Europe	2012	1.002655	47.49475	0.765423
Eastern Europe	2013	1.049628	47.82912	0.770338
Eastern Europe	2014	1.158141	49.16047	0.775092
Eastern Europe	2015	1.314594	51.69763	0.779663
Eastern Europe	2016	1.437618	53.76496	0.784805
Eastern Europe	2017	1.489699	54.50574	0.790015
Eastern Europe	2018	1.509037	54.98036	0.794486
Eastern Europe	2019	1.515915	55.02678	0.798138
Eastern Europe	2020	1.535403	55.89468	0.800505
Eastern Europe	2021	1.56574	57.20158	0.802851
Eastern Sub-Saharan Africa	1990	3.090924	72.36761	0.233622
Eastern Sub-Saharan Africa	1991	3.14768	73.72488	0.23634
Eastern Sub-Saharan Africa	1992	3.213277	75.27063	0.238615
Eastern Sub-Saharan Africa	1993	3.279924	76.87881	0.240817
Eastern Sub-Saharan Africa	1994	3.340165	78.31964	0.242902
Eastern Sub-Saharan Africa	1995	3.397359	79.61606	0.245424
Eastern Sub-Saharan Africa	1996	3.44541	80.63185	0.248585
Eastern Sub-Saharan Africa	1997	3.497913	81.73999	0.252145
Eastern Sub-Saharan Africa	1998	3.566465	83.24844	0.255891
Eastern Sub-Saharan Africa	1999	3.607844	84.05988	0.259957
Eastern Sub-Saharan Africa	2000	3.644799	84.73709	0.264165
Eastern Sub-Saharan Africa	2001	3.64665	84.68715	0.268772
Eastern Sub-Saharan Africa	2002	3.706435	85.85405	0.273484
Eastern Sub-Saharan Africa	2003	3.773368	87.40167	0.278434
Eastern Sub-Saharan Africa	2004	3.834787	88.77366	0.283937

Eastern Sub-Saharan Africa	2005	3.87908	89.68862	0.28997
Eastern Sub-Saharan Africa	2006	3.917556	90.45652	0.296455
Eastern Sub-Saharan Africa	2007	3.938466	90.98455	0.303576
Eastern Sub-Saharan Africa	2008	3.977673	91.80875	0.310918
Eastern Sub-Saharan Africa	2009	4.033104	92.99874	0.318457
Eastern Sub-Saharan Africa	2010	4.114542	94.64031	0.326296
Eastern Sub-Saharan Africa	2011	4.186901	96.07778	0.334253
Eastern Sub-Saharan Africa	2012	4.253764	97.45339	0.341628
Eastern Sub-Saharan Africa	2013	4.348886	99.36415	0.349207
Eastern Sub-Saharan Africa	2014	4.437057	101.227	0.35701
Eastern Sub-Saharan Africa	2015	4.526316	103.0144	0.364907
Eastern Sub-Saharan Africa	2016	4.6212	105.0891	0.372759
Eastern Sub-Saharan Africa	2017	4.74496	107.6758	0.3805
Eastern Sub-Saharan Africa	2018	4.870165	110.2441	0.388355
Eastern Sub-Saharan Africa	2019	4.988122	112.6346	0.396345
Eastern Sub-Saharan Africa	2020	5.132	115.3563	0.40348
Eastern Sub-Saharan Africa	2021	5.224649	117.8063	0.409721
Global	1990	2.689984	69.12854	0.525529
Global	1991	2.715772	69.8249	0.530272
Global	1992	2.756532	70.81899	0.534637
Global	1993	2.805406	71.94572	0.538598
Global	1994	2.850416	73.01109	0.542389
Global	1995	2.918472	74.37048	0.546328
Global	1996	2.981544	75.74801	0.550703
Global	1997	3.050921	77.38963	0.555139
Global	1998	3.173216	80.22696	0.559381
Global	1999	3.298461	83.01323	0.563738
Global	2000	3.478083	86.1896	0.568003
Global	2001	3.593507	88.47526	0.571778
Global	2002	3.688762	90.55967	0.575688
Global	2003	3.782798	92.84082	0.579907
Global	2004	3.83287	94.26294	0.584379
Global	2005	3.891844	95.79192	0.588979
Global	2006	3.933736	96.84867	0.593757
Global	2007	4.006371	98.38683	0.598641
Global	2008	4.104543	100.4649	0.60337
Global	2009	4.187086	102.216	0.607759
Global	2010	4.281504	103.9692	0.612952
Global	2011	4.385663	106.0673	0.617759
Global	2012	4.457524	107.7465	0.621845
Global	2013	4.558628	109.9644	0.626343
Global	2014	4.642576	111.7297	0.630982
Global	2015	4.754306	114.2518	0.635441
Global	2016	4.848139	116.2942	0.640154

Global	2017	4.884785	116.8672	0.645564
Global	2018	4.912137	117.3141	0.651048
Global	2019	4.976728	118.6949	0.656576
Global	2020	5.019661	120.3698	0.661344
Global	2021	5.056003	122.0761	0.665821
High-income Asia Pacific	1990	2.067764	46.59604	0.767804
High-income Asia Pacific	1991	2.093255	46.69421	0.773333
High-income Asia Pacific	1992	2.079117	46.31062	0.778834
High-income Asia Pacific	1993	2.051139	45.81516	0.783967
High-income Asia Pacific	1994	2.013322	45.26422	0.789028
High-income Asia Pacific	1995	2.168167	48.47676	0.794099
High-income Asia Pacific	1996	2.151719	48.28697	0.798863
High-income Asia Pacific	1997	2.078485	47.18703	0.803128
High-income Asia Pacific	1998	2.050674	46.78298	0.806831
High-income Asia Pacific	1999	2.022299	46.35124	0.810469
High-income Asia Pacific	2000	1.964151	45.33767	0.814089
High-income Asia Pacific	2001	1.923531	44.66726	0.817545
High-income Asia Pacific	2002	1.900657	44.17023	0.82111
High-income Asia Pacific	2003	1.900363	44.10425	0.824636
High-income Asia Pacific	2004	1.908736	44.11635	0.828074
High-income Asia Pacific	2005	1.928665	44.38544	0.831062
High-income Asia Pacific	2006	1.930143	44.40435	0.833766
High-income Asia Pacific	2007	1.938399	44.57669	0.836546
High-income Asia Pacific	2008	1.951322	44.80927	0.83928
High-income Asia Pacific	2009	1.965937	45.09917	0.841771
High-income Asia Pacific	2010	2.003419	45.76181	0.844682
High-income Asia Pacific	2011	2.035962	46.31802	0.847722
High-income Asia Pacific	2012	2.032921	46.44501	0.850693
High-income Asia Pacific	2013	2.003696	46.15121	0.853671
High-income Asia Pacific	2014	1.977338	45.88683	0.856612
High-income Asia Pacific	2015	1.946699	45.52267	0.859603
High-income Asia Pacific	2016	1.950933	45.30517	0.862733
High-income Asia Pacific	2017	1.954997	44.79997	0.86587
High-income Asia Pacific	2018	1.971786	44.49305	0.869073
High-income Asia Pacific	2019	1.977969	44.25361	0.872219
High-income Asia Pacific	2020	1.922553	43.28133	0.874437
High-income Asia Pacific	2021	1.966349	43.85411	0.876767
High-income North America	1990	2.973784	81.26211	0.76566
High-income North America	1991	3.04098	82.98042	0.76805
High-income North America	1992	3.141529	85.38418	0.77155
High-income North America	1993	3.268942	88.35131	0.774978
High-income North America	1994	3.333052	90.11737	0.778207
High-income North America	1995	3.408627	92.25212	0.781348
High-income North America	1996	3.477262	94.41095	0.784105

High-income North America	1997	3.588712	97.71779	0.786348
High-income North America	1998	3.788288	102.9365	0.788569
High-income North America	1999	4.254672	113.8384	0.791196
High-income North America	2000	4.804872	124.3994	0.794997
High-income North America	2001	5.094076	129.835	0.799275
High-income North America	2002	5.242324	132.4545	0.802961
High-income North America	2003	5.337361	134.4432	0.805821
High-income North America	2004	5.353787	134.6632	0.807991
High-income North America	2005	5.433182	136.0487	0.808887
High-income North America	2006	5.530193	137.9207	0.809721
High-income North America	2007	5.61119	138.9403	0.812486
High-income North America	2008	5.738293	141.2965	0.817084
High-income North America	2009	5.892182	143.9885	0.82247
High-income North America	2010	6.196163	149.8339	0.827814
High-income North America	2011	6.86469	164.1951	0.832061
High-income North America	2012	7.249488	172.9017	0.835669
High-income North America	2013	7.754185	184.7763	0.839059
High-income North America	2014	8.111004	193.1615	0.842341
High-income North America	2015	8.398	200.0118	0.845877
High-income North America	2016	8.677038	204.909	0.849423
High-income North America	2017	8.850774	206.3899	0.85296
High-income North America	2018	8.932888	205.8681	0.856316
High-income North America	2019	9.098413	207.5744	0.859749
High-income North America	2020	9.169229	208.2061	0.861874
High-income North America	2021	9.258811	209.7956	0.863465
North Africa and Middle East	1990	8.652996	190.6778	0.437421
North Africa and Middle East	1991	8.599519	191.5308	0.445921
North Africa and Middle East	1992	8.619585	192.8418	0.453971
North Africa and Middle East	1993	8.765917	196.3262	0.462102
North Africa and Middle East	1994	8.88358	199.166	0.470394
North Africa and Middle East	1995	8.956213	200.803	0.478553
North Africa and Middle East	1996	9.052504	203.3965	0.486463
North Africa and Middle East	1997	9.147202	206.1799	0.49398
North Africa and Middle East	1998	9.206257	208.085	0.501445
North Africa and Middle East	1999	9.25422	210.2174	0.508956
North Africa and Middle East	2000	9.275658	211.0216	0.516824
North Africa and Middle East	2001	9.433614	214.9779	0.524091
North Africa and Middle East	2002	9.567828	218.1624	0.531149
North Africa and Middle East	2003	9.6793	221.0713	0.538292
North Africa and Middle East	2004	9.767822	223.6962	0.545678
North Africa and Middle East	2005	9.902156	227.1639	0.553047
North Africa and Middle East	2006	10.104	231.5486	0.560089
North Africa and Middle East	2007	10.32954	235.5199	0.566516
North Africa and Middle East	2008	10.71178	242.7975	0.572339

North Africa and Middle East	2009	11.164	251.7642	0.577062
North Africa and Middle East	2010	11.42668	257.4942	0.581849
North Africa and Middle East	2011	11.65257	261.9233	0.587593
North Africa and Middle East	2012	11.91735	266.895	0.594112
North Africa and Middle East	2013	12.08575	268.8883	0.601127
North Africa and Middle East	2014	12.57233	277.083	0.608419
North Africa and Middle East	2015	13.14352	287.2853	0.615777
North Africa and Middle East	2016	13.45121	291.9683	0.623142
North Africa and Middle East	2017	13.59025	293.0999	0.630561
North Africa and Middle East	2018	13.85778	298.1809	0.637918
North Africa and Middle East	2019	14.23288	304.4636	0.645095
North Africa and Middle East	2020	14.50241	308.9224	0.651673
North Africa and Middle East	2021	14.65149	311.8313	0.658225
Oceania	1990	3.550896	95.7931	0.391195
Oceania	1991	3.681073	98.78812	0.394419
Oceania	1992	3.779251	101.0755	0.397572
Oceania	1993	3.877158	103.4666	0.401113
Oceania	1994	3.972802	105.7958	0.404892
Oceania	1995	4.101323	108.5726	0.408507
Oceania	1996	4.24566	111.9462	0.412437
Oceania	1997	4.331117	114.1126	0.415893
Oceania	1998	4.415413	115.8142	0.419225
Oceania	1999	4.579402	119.838	0.422521
Oceania	2000	4.696427	122.7317	0.42507
Oceania	2001	4.740539	123.8259	0.427004
Oceania	2002	4.819923	125.7441	0.428535
Oceania	2003	4.82126	126.0158	0.43006
Oceania	2004	4.806149	125.9412	0.431581
Oceania	2005	4.847788	127.6119	0.433152
Oceania	2006	4.943725	129.9311	0.434584
Oceania	2007	4.961968	130.4073	0.436102
Oceania	2008	4.973689	130.8445	0.437392
Oceania	2009	4.941008	129.9848	0.438808
Oceania	2010	5.003467	131.1821	0.440752
Oceania	2011	5.09536	132.7871	0.442618
Oceania	2012	5.134119	133.4716	0.444524
Oceania	2013	5.213369	134.7704	0.446594
Oceania	2014	5.317397	136.5571	0.449364
Oceania	2015	5.385039	138.0449	0.452397
Oceania	2016	5.44125	138.721	0.455454
Oceania	2017	5.525455	139.7338	0.458378
Oceania	2018	5.584563	140.413	0.460818
Oceania	2019	5.639985	141.9461	0.463334
Oceania	2020	5.554066	140.9203	0.465535

Oceania	2021	5.578789	140.7292	0.467445
South Asia	1990	1.074491	33.15289	0.319797
South Asia	1991	1.099467	33.70672	0.325854
South Asia	1992	1.142024	34.62395	0.331941
South Asia	1993	1.175018	35.44999	0.338013
South Asia	1994	1.219532	36.55685	0.344323
South Asia	1995	1.284712	37.97674	0.350726
South Asia	1996	1.319574	39.04928	0.357287
South Asia	1997	1.354136	40.34961	0.363719
South Asia	1998	1.379371	41.44854	0.370288
South Asia	1999	1.377601	42.03117	0.376979
South Asia	2000	1.397471	42.91399	0.383388
South Asia	2001	1.45343	44.50527	0.389533
South Asia	2002	1.501438	46.11779	0.395194
South Asia	2003	1.559338	47.84203	0.400983
South Asia	2004	1.611453	49.36709	0.407181
South Asia	2005	1.659122	50.97638	0.413987
South Asia	2006	1.677353	51.83364	0.421367
South Asia	2007	1.730089	53.51227	0.429262
South Asia	2008	1.75191	54.78517	0.437012
South Asia	2009	1.759208	55.798	0.445197
South Asia	2010	1.787069	57.02199	0.454076
South Asia	2011	1.810827	58.00906	0.463339
South Asia	2012	1.840413	59.09158	0.473081
South Asia	2013	1.951626	61.43141	0.483434
South Asia	2014	2.047191	63.20355	0.494084
South Asia	2015	2.095403	64.89548	0.504891
South Asia	2016	2.123069	65.74084	0.515311
South Asia	2017	2.175288	66.85783	0.525128
South Asia	2018	2.246015	68.40709	0.534477
South Asia	2019	2.332827	70.2316	0.543246
South Asia	2020	2.34672	71.91587	0.550586
South Asia	2021	2.359905	72.96649	0.557865
Southeast Asia	1990	1.846246	50.44858	0.464104
Southeast Asia	1991	1.880109	51.34518	0.47175
Southeast Asia	1992	1.934734	52.72072	0.47949
Southeast Asia	1993	1.991194	54.21394	0.487349
Southeast Asia	1994	2.046207	55.62089	0.495348
Southeast Asia	1995	2.110123	57.27727	0.503351
Southeast Asia	1996	2.179898	59.0632	0.511233
Southeast Asia	1997	2.229049	60.37571	0.518664
Southeast Asia	1998	2.29946	62.12842	0.524439
Southeast Asia	1999	2.406681	64.98281	0.529668
Southeast Asia	2000	2.472858	66.8087	0.534567

Southeast Asia	2001	2.523557	68.1673	0.53901
Southeast Asia	2002	2.623815	70.785	0.543479
Southeast Asia	2003	2.711369	73.1777	0.548051
Southeast Asia	2004	2.79728	75.50258	0.552707
Southeast Asia	2005	2.894017	77.90423	0.557391
Southeast Asia	2006	2.979688	79.97954	0.562302
Southeast Asia	2007	3.033987	81.34963	0.567654
Southeast Asia	2008	3.126048	83.67424	0.573208
Southeast Asia	2009	3.201092	85.6313	0.578497
Southeast Asia	2010	3.277038	87.53834	0.584268
Southeast Asia	2011	3.32885	89.03268	0.590415
Southeast Asia	2012	3.360801	90.13479	0.59678
Southeast Asia	2013	3.421967	91.82895	0.603145
Southeast Asia	2014	3.489078	93.72444	0.609439
Southeast Asia	2015	3.569876	95.73097	0.615689
Southeast Asia	2016	3.664469	97.90705	0.62184
Southeast Asia	2017	3.743396	99.48771	0.627978
Southeast Asia	2018	3.845882	101.4615	0.634096
Southeast Asia	2019	3.980486	104.232	0.640107
Southeast Asia	2020	3.982725	104.2704	0.64507
Southeast Asia	2021	4.113745	106.9224	0.649777
Southern Latin America	1990	7.839686	165.3849	0.587308
Southern Latin America	1991	8.120433	169.4913	0.592119
Southern Latin America	1992	8.380326	173.4851	0.59808
Southern Latin America	1993	8.345032	172.2645	0.603589
Southern Latin America	1994	8.164622	168.6225	0.609392
Southern Latin America	1995	8.189283	168.6034	0.614373
Southern Latin America	1996	8.411357	172.9488	0.61926
Southern Latin America	1997	9.203809	186.8581	0.624756
Southern Latin America	1998	9.766595	196.3398	0.630349
Southern Latin America	1999	10.07398	201.1954	0.635748
Southern Latin America	2000	10.3335	204.6251	0.641237
Southern Latin America	2001	10.73856	212.7995	0.64604
Southern Latin America	2002	11.10899	219.8683	0.649826
Southern Latin America	2003	11.18981	221.1478	0.652552
Southern Latin America	2004	11.06353	218.6612	0.655719
Southern Latin America	2005	10.89987	216.5011	0.660519
Southern Latin America	2006	11.00272	218.6511	0.664386
Southern Latin America	2007	11.30071	224.6143	0.667393
Southern Latin America	2008	10.98667	219.0855	0.670689
Southern Latin America	2009	11.04252	220.0993	0.674152
Southern Latin America	2010	11.1695	220.8526	0.678761
Southern Latin America	2011	10.94912	215.6657	0.684134
Southern Latin America	2012	10.8594	214.6092	0.688428

Southern Latin America	2013	10.83488	214.1307	0.691716
Southern Latin America	2014	10.635	209.5839	0.69604
Southern Latin America	2015	10.47703	207.0497	0.703287
Southern Latin America	2016	10.55053	207.8192	0.710788
Southern Latin America	2017	10.26877	202.1247	0.717548
Southern Latin America	2018	10.10349	199.7263	0.724952
Southern Latin America	2019	10.11823	199.936	0.731134
Southern Latin America	2020	9.591388	192.0034	0.733963
Southern Latin America	2021	9.080212	183.1365	0.735985
Southern Sub-Saharan Africa	1990	4.07843	115.8058	0.506947
Southern Sub-Saharan Africa	1991	4.300933	121.4568	0.512378
Southern Sub-Saharan Africa	1992	4.763995	132.6776	0.517569
Southern Sub-Saharan Africa	1993	4.874723	133.5256	0.522773
Southern Sub-Saharan Africa	1994	5.328904	143.1519	0.528063
Southern Sub-Saharan Africa	1995	5.527935	146.4483	0.53338
Southern Sub-Saharan Africa	1996	6.080129	155.6769	0.53878
Southern Sub-Saharan Africa	1997	6.922596	173.9885	0.544134
Southern Sub-Saharan Africa	1998	7.248322	180.3872	0.549142
Southern Sub-Saharan Africa	1999	7.34881	182.0776	0.553885
Southern Sub-Saharan Africa	2000	7.851772	194.565	0.558491
Southern Sub-Saharan Africa	2001	8.047008	199.0178	0.562511
Southern Sub-Saharan Africa	2002	8.407717	208.8673	0.56605
Southern Sub-Saharan Africa	2003	8.807575	220.1786	0.569215
Southern Sub-Saharan Africa	2004	9.005399	226.8569	0.57239
Southern Sub-Saharan Africa	2005	9.212618	231.9251	0.576116
Southern Sub-Saharan Africa	2006	9.517468	238.0102	0.580685
Southern Sub-Saharan Africa	2007	9.72109	240.9071	0.585673
Southern Sub-Saharan Africa	2008	10.0119	246.9345	0.590405
Southern Sub-Saharan Africa	2009	10.29252	252.093	0.594585
Southern Sub-Saharan Africa	2010	10.46021	254.3002	0.598836
Southern Sub-Saharan Africa	2011	10.55491	254.8257	0.603347
Southern Sub-Saharan Africa	2012	10.68503	256.8386	0.608038
Southern Sub-Saharan Africa	2013	10.82419	259.0264	0.612844
Southern Sub-Saharan Africa	2014	11.12612	264.8564	0.617453
Southern Sub-Saharan Africa	2015	11.43349	270.7413	0.621838
Southern Sub-Saharan Africa	2016	11.53028	272.6037	0.625919
Southern Sub-Saharan Africa	2017	11.34418	267.8079	0.629762
Southern Sub-Saharan Africa	2018	11.30576	266.9636	0.633411
Southern Sub-Saharan Africa	2019	11.07164	262.2014	0.63694
Southern Sub-Saharan Africa	2020	11.26514	267.188	0.639882
Southern Sub-Saharan Africa	2021	11.12128	268.3308	0.6422
Tropical Latin America	1990	5.514091	148.6069	0.499588
Tropical Latin America	1991	5.458581	146.8251	0.50429
Tropical Latin America	1992	5.567383	149.7906	0.508057

Tropical Latin America	1993	5.728565	153.6128	0.511622
Tropical Latin America	1994	5.759941	154.728	0.51533
Tropical Latin America	1995	5.85206	156.8505	0.519291
Tropical Latin America	1996	6.305727	167.7652	0.523499
Tropical Latin America	1997	6.555818	174.0098	0.527841
Tropical Latin America	1998	6.771527	179.4406	0.532165
Tropical Latin America	1999	7.016422	184.6962	0.536659
Tropical Latin America	2000	7.265584	188.5712	0.541716
Tropical Latin America	2001	7.339627	188.9811	0.546932
Tropical Latin America	2002	7.351104	188.4301	0.552365
Tropical Latin America	2003	7.423893	189.1969	0.557691
Tropical Latin America	2004	7.448493	189.507	0.563269
Tropical Latin America	2005	7.260496	184.8748	0.568897
Tropical Latin America	2006	7.108829	181.2188	0.57472
Tropical Latin America	2007	7.122728	181.1761	0.580915
Tropical Latin America	2008	7.214502	182.6962	0.587354
Tropical Latin America	2009	7.393627	186.0258	0.593303
Tropical Latin America	2010	7.643988	189.465	0.599646
Tropical Latin America	2011	7.817656	192.2258	0.605957
Tropical Latin America	2012	7.698904	188.8309	0.611787
Tropical Latin America	2013	7.770331	189.7657	0.617402
Tropical Latin America	2014	7.777764	189.4911	0.622646
Tropical Latin America	2015	7.926751	192.066	0.6274
Tropical Latin America	2016	8.136357	196.0417	0.631533
Tropical Latin America	2017	8.065361	193.6485	0.635765
Tropical Latin America	2018	8.083075	193.2486	0.64018
Tropical Latin America	2019	8.14286	193.6444	0.644694
Tropical Latin America	2020	8.334511	198.9927	0.648603
Tropical Latin America	2021	8.12426	197.7917	0.652442
Western Europe	1990	2.134066	55.17373	0.7464
Western Europe	1991	2.072588	54.05481	0.75167
Western Europe	1992	2.024644	53.17605	0.757022
Western Europe	1993	2.030004	53.2332	0.762057
Western Europe	1994	2.045583	53.43573	0.766693
Western Europe	1995	2.078916	53.70682	0.770622
Western Europe	1996	2.109117	53.98037	0.77408
Western Europe	1997	2.135929	54.21488	0.777578
Western Europe	1998	2.231056	55.71643	0.780867
Western Europe	1999	2.321463	56.96529	0.784019
Western Europe	2000	2.519852	59.1796	0.7874
Western Europe	2001	2.622287	60.32388	0.790904
Western Europe	2002	2.714559	61.38179	0.794165
Western Europe	2003	2.762127	61.8801	0.796962
Western Europe	2004	2.741853	61.44746	0.799716

Western Europe	2005	2.740701	61.27411	0.802414
Western Europe	2006	2.753228	61.16952	0.805067
Western Europe	2007	2.793453	61.40314	0.807723
Western Europe	2008	2.86557	62.04091	0.810484
Western Europe	2009	2.865172	61.71892	0.81311
Western Europe	2010	2.929142	62.24591	0.816142
Western Europe	2011	2.988706	62.81043	0.819375
Western Europe	2012	3.065412	63.63305	0.822634
Western Europe	2013	3.123717	64.36934	0.825787
Western Europe	2014	3.20455	65.43507	0.82861
Western Europe	2015	3.306569	66.79879	0.831322
Western Europe	2016	3.301342	66.63633	0.834216
Western Europe	2017	3.28848	66.56891	0.837522
Western Europe	2018	3.309278	67.04832	0.840834
Western Europe	2019	3.304653	67.19317	0.844212
Western Europe	2020	3.318791	67.16484	0.84655
Western Europe	2021	3.227886	66.6411	0.848726
Western Sub-Saharan Africa	1990	4.672312	111.9861	0.2737
Western Sub-Saharan Africa	1991	4.792258	114.5912	0.277411
Western Sub-Saharan Africa	1992	4.942234	117.7693	0.281025
Western Sub-Saharan Africa	1993	5.095265	121.073	0.284432
Western Sub-Saharan Africa	1994	5.238956	124.2248	0.2877
Western Sub-Saharan Africa	1995	5.349468	126.7339	0.290983
Western Sub-Saharan Africa	1996	5.453605	129.2977	0.294618
Western Sub-Saharan Africa	1997	5.592813	132.7771	0.298302
Western Sub-Saharan Africa	1998	5.735584	136.4972	0.302026
Western Sub-Saharan Africa	1999	5.822318	139.0386	0.30581
Western Sub-Saharan Africa	2000	5.910121	141.0338	0.309781
Western Sub-Saharan Africa	2001	5.946691	142.6413	0.314028
Western Sub-Saharan Africa	2002	6.028787	143.9163	0.318881
Western Sub-Saharan Africa	2003	6.048507	144.4701	0.324233
Western Sub-Saharan Africa	2004	6.090517	145.172	0.330228
Western Sub-Saharan Africa	2005	6.14187	146.0252	0.33678
Western Sub-Saharan Africa	2006	6.248631	147.6679	0.343248
Western Sub-Saharan Africa	2007	6.281479	148.7335	0.35004
Western Sub-Saharan Africa	2008	6.372733	150.0388	0.356716
Western Sub-Saharan Africa	2009	6.460837	152.1044	0.363492
Western Sub-Saharan Africa	2010	6.599116	155.1511	0.370609
Western Sub-Saharan Africa	2011	6.7459	158.4182	0.377609
Western Sub-Saharan Africa	2012	6.899468	162.1815	0.384753
Western Sub-Saharan Africa	2013	7.053541	165.9642	0.39189
Western Sub-Saharan Africa	2014	7.172637	169.2631	0.399162
Western Sub-Saharan Africa	2015	7.288604	171.2358	0.406238
Western Sub-Saharan Africa	2016	7.455081	174.8393	0.412927

Western Sub-Saharan Africa	2017	7.626992	178.0342	0.419615
Western Sub-Saharan Africa	2018	7.793932	180.947	0.426437
Western Sub-Saharan Africa	2019	7.923207	183.1918	0.433384
Western Sub-Saharan Africa	2020	8.043986	185.5384	0.439822
Western Sub-Saharan Africa	2021	8.171752	188.7163	0.446023

Table S10

Decomposition analysis of high BMI-attributable CKD burden in global from 1990 to 2021.

location	Varname	Death			DALY		
		Both Val	Female Val	Male Val	Both Val	Female Val	Male Val
Global	Aging	72.03508	66.6342	80.20443	44.01641222	41.11830566	47.92431778
	Population	137.3634	136.9877	137.9865	124.9648009	124.2579958	125.8424934
	Epidemiological change	145.4541	143.9744	145.2547	121.3354793	116.835817	125.5730875
High SDI	Aging	104.1662	94.95628	125.4226	59.59289605	54.15640893	70.3778697
	Population	84.41119	76.06376	94.28961	71.112527	64.02997738	78.99111789
	Epidemiological change	166.1269	158.0849	167.5528	118.6372383	109.8405987	123.2021577
High-middle SDI	Aging	81.72755	78.04684	90.35332	50.19188842	47.10229819	55.44536622
	Population	87.34242	87.45952	87.17849	76.50783712	75.26863172	77.89815363
	Epidemiological change	80.45485	95.98323	57.64081	54.04168036	60.40865173	44.99474396
Middle SDI	Aging	104.6824	103.671	104.9901	68.36131474	69.72998544	66.55438744
	Population	171.2946	175.3052	167.3597	157.5618045	161.7794014	153.4160156
	Epidemiological change	157.1319	148.1808	167.4476	141.610831	133.1871898	150.63674
Low-middle SDI	Aging	44.49546	53.42261	34.60947	28.70304577	35.19453623	21.86449015
	Population	188.7062	194.617	182.8939	184.1778771	189.3744424	179.0729724
	Epidemiological change	187.3604	173.94	201.4556	185.7724907	173.069629	198.7789937
Low SDI	Aging	-6.62693	-1.35594	-12.1629	-8.354724494	-4.037256729	-12.92281324
	Population	169.9125	170.4678	169.431	171.7581719	169.752562	174.2186674
	Epidemiological change	80.3515	68.77231	92.48277	85.99131359	69.03239558	104.5790627

Table S11

Projections of high BMI-attributable CKD DALY burden by sex of all age groups from 2022-2035 globally.

year	Female			Male		
	mean	lower	upper	mean	lower	upper
2022	118.2774	116.0675	120.4873	130.2109	127.7683	132.6535
2023	120.0694	116.2445	123.8944	131.7192	127.7011	135.7374
2024	121.9048	116.0032	127.8063	133.2704	127.2253	139.3156
2025	123.7671	115.4098	132.1243	134.8523	126.4176	143.287
2026	125.6923	114.5256	136.8589	136.4841	125.3291	147.6392
2027	127.7086	113.3813	142.0359	138.1904	123.9925	152.3883
2028	129.8258	111.9964	147.6552	139.9951	122.4453	157.5448
2029	132.0088	110.3435	153.674	141.8577	120.6585	163.057
2030	134.236	108.404	160.0681	143.7619	118.6196	168.9043
2031	136.5351	106.1927	166.8776	145.7224	116.3359	175.1088
2032	138.9364	103.7181	174.1546	147.7603	113.8141	181.7065
2033	141.4605	100.9896	181.9314	149.9014	111.073	188.7298
2034	144.0783	97.97633	190.1804	152.1115	108.0825	196.1404
2035	146.7668	94.65194	198.8818	154.3752	104.8254	203.9251

Table S12

Projections of high BMI-attributable CKD death burden by sex of all age groups from 2022-2035 globally.

year	Female			Male		
	mean	lower	upper	mean	lower	upper
2022	4.831801	4.725827	4.937776	5.516071	5.401732	5.630411
2023	4.884311	4.700238	5.068384	5.557993	5.366726	5.749261
2024	4.937724	4.65474	5.220707	5.601443	5.312792	5.890094
2025	4.991465	4.592648	5.390283	5.646094	5.24376	6.048427
2026	5.046166	4.516012	5.57632	5.692096	5.161381	6.222811
2027	5.1022	4.425678	5.778722	5.740175	5.06692	6.41343
2028	5.160537	4.323299	5.997775	5.791352	4.962188	6.620516
2029	5.220417	4.208652	6.232181	5.844235	4.846471	6.841999
2030	5.28105	4.08131	6.48079	5.898515	4.719766	7.077264
2031	5.342098	3.940979	6.743217	5.953824	4.581734	7.325914
2032	5.403636	3.787484	7.019788	6.010428	4.432336	7.58852
2033	5.467694	3.622436	7.312951	6.06947	4.272651	7.86629
2034	5.53435	3.445798	7.622903	6.130333	4.10228	8.158386
2035	5.60307	3.257011	7.949128	6.1932	3.921259	8.465142

Table S13

Projections of high BMI-attributable CKD death burden for females with differences age groups from 2022-2035 globally.

	2022	2023	2024	2025	2026	2027	2028	2029	2030	2031	2032	2033	2034	2035
25-29	0.26 (0.24, 0.28)	0.27 (0.25, 0.29)	0.28 (0.25, 0.31)	0.28 (0.25, 0.33)	0.29 (0.25, 0.35)	0.3 (0.25, 0.37)	0.31 (0.25, 0.39)	0.32 (0.24, 0.42)	0.33 (0.24, 0.45)	0.34 (0.24, 0.48)	0.35 (0.23, 0.52)	0.36 (0.23, 0.56)	0.38 (0.22, 0.6)	0.39 (0.22, 0.65)
30-34	0.39 (0.37, 0.41)	0.4 (0.38, 0.43)	0.41 (0.38, 0.44)	0.43 (0.39, 0.47)	0.44 (0.39, 0.49)	0.45 (0.39, 0.52)	0.46 (0.39, 0.56)	0.48 (0.38, 0.59)	0.49 (0.38, 0.63)	0.51 (0.37, 0.68)	0.52 (0.37, 0.72)	0.54 (0.36, 0.78)	0.56 (0.36, 0.84)	0.58 (0.35, 0.9)
35-39	0.64 (0.62, 0.67)	0.66 (0.62, 0.69)	0.67 (0.62, 0.72)	0.68 (0.63, 0.75)	0.7 (0.63, 0.78)	0.72 (0.62, 0.83)	0.74 (0.62, 0.87)	0.76 (0.62, 0.93)	0.78 (0.62, 0.98)	0.81 (0.61, 1.05)	0.83 (0.6, 1.12)	0.86 (0.6, 1.2)	0.89 (0.59, 1.29)	0.92 (0.58, 1.39)
40-44	1.19 (1.15, 1.24)	1.21 (1.15, 1.27)	1.23 (1.15, 1.31)	1.25 (1.14, 1.36)	1.26 (1.13, 1.41)	1.29 (1.12, 1.47)	1.31 (1.1, 1.54)	1.34 (1.09, 1.62)	1.37 (1.08, 1.71)	1.4 (1.06, 1.82)	1.44 (1.05, 1.94)	1.49 (1.04, 2.07)	1.53 (1.03, 2.21)	1.58 (1.01, 2.37)
45-49	2.17 (2.09, 2.26)	2.22 (2.11, 2.33)	2.26 (2.12, 2.42)	2.3 (2.11, 2.51)	2.34 (2.1, 2.62)	2.38 (2.07, 2.73)	2.42 (2.04, 2.85)	2.46 (2, 2.98)	2.5 (1.97, 3.13)	2.54 (1.93, 3.29)	2.59 (1.88, 3.47)	2.64 (1.84, 3.67)	2.7 (1.81, 3.89)	2.77 (1.77, 4.14)
50-54	4.01 (3.86, 4.16)	4.08 (3.89, 4.28)	4.17 (3.9, 4.45)	4.26 (3.9, 4.64)	4.35 (3.89, 4.85)	4.44 (3.86, 5.09)	4.54 (3.83, 5.35)	4.64 (3.78, 5.63)	4.73 (3.73, 5.92)	4.82 (3.66, 6.24)	4.91 (3.58, 6.57)	4.99 (3.49, 6.93)	5.08 (3.4, 7.32)	5.17 (3.31, 7.74)

55-59	6.97 (6.72, 7.24)	7.02 (6.69, 7.36)	7.08 (6.63, 7.55)	7.16 (6.57, 7.8)	7.27 (6.5, 8.11)	7.4 (6.43, 8.47)	7.55 (6.37, 8.89)	7.71 (6.29, 9.36)	7.89 (6.22, 9.88)	8.07 (6.13, 10.45)	8.27 (6.03, 11.08)	8.46 (5.92, 11.75)	8.66 (5.8, 12.47)	8.85 (5.66, 13.24)
60-64	11.02 (10.62, 11.43)	11.11 (10.59, 11.65)	11.19 (10.49, 11.94)	11.27 (10.33, 12.27)	11.33 (10.14, 12.63)	11.4 (9.92, 13.05)	11.48 (9.69, 13.52)	11.6 (9.47, 14.07)	11.75 (9.26, 14.72)	11.94 (9.07, 15.46)	12.18 (8.88, 16.32)	12.45 (8.71, 17.28)	12.75 (8.53, 18.36)	13.07 (8.36, 19.55)
65-69	16.45 (15.85, 17.06)	16.66 (15.88, 17.47)	16.89 (15.82, 18.01)	17.12 (15.7, 18.63)	17.32 (15.5, 19.31)	17.5 (15.23, 20.03)	17.67 (14.91, 20.8)	17.82 (14.55, 21.62)	17.96 (14.16, 22.5)	18.1 (13.74, 23.43)	18.23 (13.3, 24.43)	18.41 (12.88, 25.56)	18.63 (12.47, 26.83)	18.93 (12.1, 28.3)
70-74	24.35 (23.48, 25.25)	24.52 (23.37, 25.71)	24.72 (23.16, 26.36)	24.96 (22.89, 27.17)	25.23 (22.57, 28.12)	25.54 (22.22, 29.23)	25.9 (21.86, 30.5)	26.29 (21.46, 31.9)	26.68 (21.03, 33.41)	27.05 (20.53, 35.01)	27.38 (19.97, 36.68)	27.69 (19.37, 38.45)	28 (18.74, 40.32)	28.29 (18.09, 42.3)
75-79	37.76 (36.41, 39.16)	37.95 (36.18, 39.8)	38.1 (35.7, 40.62)	38.26 (35.09, 41.65)	38.46 (34.41, 42.87)	38.71 (33.68, 44.3)	39.01 (32.92, 45.93)	39.38 (32.15, 47.78)	39.81 (31.38, 49.86)	40.31 (30.6, 52.19)	40.89 (29.83, 54.79)	41.55 (29.07, 57.69)	42.27 (28.3, 60.87)	43 (27.49, 64.29)
80-84	54.82 (52.86, 56.85)	55.79 (53.18, 58.5)	56.77 (53.19, 60.53)	57.65 (52.88, 62.76)	58.36 (52.21, 65.06)	58.86 (51.21, 67.36)	59.21 (49.97, 69.71)	59.51 (48.59, 72.21)	59.85 (47.17, 74.95)	60.26 (45.75, 78.01)	60.76 (44.32, 81.41)	61.37 (42.93, 85.2)	62.08 (41.56, 89.41)	62.92 (40.22, 94.07)
85-89	104.5 (100.75, 108.35)	105.41 (100.47, 110.53)	106.44 (99.74, 113.5)	107.67 (98.76, 117.2)	109.18 (97.68, 121.7)	111.01 (96.58, 127.04)	113.08 (95.42, 133.14)	115.2 (94.05, 139.78)	117.16 (92.34, 146.73)	118.81 (90.18, 153.8)	120.03 (87.56, 160.83)	121.01 (84.65, 168)	121.89 (81.6, 175.54)	122.88 (78.56, 183.72)
90-94	198.6 (191.46, 205.92)	201.31 (191.87, 211.1)	203.57 (190.74, 217.06)	205.3 (188.29, 223.48)	206.89 (185.09, 230.62)	208.67 (181.55, 238.8)	210.7 (177.79, 248.06)	213.03 (173.92, 258.48)	215.8 (170.07, 270.26)	219.18 (166.37, 283.72)	223.26 (162.86, 299.14)	227.9 (159.43, 316.42)	232.7 (155.77, 335.11)	237.24 (151.67, 354.7)
95+	346.68 (334.14, 359.54)	349.76 (333.3, 366.82)	353.25 (330.95, 376.7)	357.22 (327.59, 388.87)	362.01 (323.84, 403.56)	367.5 (319.71, 420.59)	372.9 (314.64, 439.05)	377.55 (308.22, 458.11)	381.31 (300.49, 477.55)	384.89 (292.15, 498.26)	388.92 (283.7, 521.11)	393.5 (275.26, 546.34)	398.76 (266.94, 574.27)	404.93 (258.87, 605.42)

Table S14

Projections of high BMI-attributable CKD death burden for males with differences age groups from 2022-2035 globally.

	2022	2023	2024	2025	2026	2027	2028	2029	2030	2031	2032	2033	2034	2035
25-29	0.38 (0.35, 0.4)	0.39 (0.36, 0.42)	0.4 (0.36, 0.44)	0.41 (0.36, 0.46)	0.42 (0.36, 0.48)	0.43 (0.36, 0.51)	0.44 (0.36, 0.54)	0.45 (0.35, 0.58)	0.47 (0.35, 0.61)	0.48 (0.34, 0.65)	0.49 (0.34, 0.7)	0.51 (0.33, 0.74)	0.52 (0.33, 0.8)	0.54 (0.32, 0.85)
30-34	0.5 (0.48, 0.52)	0.51 (0.48, 0.54)	0.52 (0.49, 0.56)	0.54 (0.49, 0.58)	0.55 (0.49, 0.61)	0.57 (0.49, 0.64)	0.58 (0.49, 0.68)	0.6 (0.49, 0.72)	0.61 (0.48, 0.76)	0.63 (0.48, 0.81)	0.65 (0.47, 0.86)	0.66 (0.47, 0.92)	0.68 (0.46, 0.98)	0.7 (0.45, 1.05)
35-39	0.85 (0.82, 0.89)	0.86 (0.82, 0.91)	0.88 (0.82, 0.93)	0.89 (0.82, 0.97)	0.91 (0.82, 1.01)	0.93 (0.82, 1.05)	0.96 (0.82, 1.11)	0.98 (0.82, 1.17)	1.01 (0.81, 1.23)	1.03 (0.81, 1.3)	1.06 (0.8, 1.38)	1.09 (0.79, 1.47)	1.12 (0.78, 1.56)	1.16 (0.77, 1.66)
40-44	1.47 (1.42, 1.53)	1.49 (1.42, 1.56)	1.51 (1.42, 1.6)	1.52 (1.41, 1.65)	1.54 (1.39, 1.7)	1.56 (1.37, 1.76)	1.58 (1.36, 1.83)	1.61 (1.34, 1.91)	1.64 (1.33, 2)	1.67 (1.31, 2.1)	1.72 (1.3, 2.22)	1.76 (1.29, 2.35)	1.81 (1.27, 2.5)	1.86 (1.26, 2.66)
45-49	2.4 (2.31, 2.49)	2.45 (2.34, 2.57)	2.5 (2.35, 2.66)	2.55 (2.35, 2.75)	2.59 (2.34, 2.86)	2.63 (2.32, 2.96)	2.66 (2.29, 3.08)	2.69 (2.25, 3.2)	2.73 (2.21, 3.32)	2.76 (2.16, 3.46)	2.8 (2.12, 3.62)	2.84 (2.07, 3.79)	2.89 (2.03, 3.99)	2.95 (1.99, 4.21)
50-54	4.44 (4.28, 4.61)	4.5 (4.3, 4.71)	4.57 (4.3, 4.85)	4.65 (4.3, 5.02)	4.74 (4.29, 5.23)	4.84 (4.27, 5.46)	4.94 (4.25, 5.71)	5.05 (4.22, 5.99)	5.15 (4.17, 6.28)	5.24 (4.11, 6.58)	5.32 (4.03, 6.89)	5.4 (3.95, 7.21)	5.48 (3.85, 7.55)	5.55 (3.75, 7.91)
55-59	7.51 (7.24, 7.79)	7.55 (7.21, 7.9)	7.6 (7.15, 8.06)	7.66 (7.08, 8.27)	7.74 (7, 8.53)	7.84 (6.92, 8.83)	7.95 (6.83, 9.18)	8.08 (6.75, 9.58)	8.23 (6.67, 10.03)	8.4 (6.58, 10.54)	8.59 (6.5, 11.11)	8.79 (6.42, 11.72)	8.99 (6.32, 12.38)	9.18 (6.2, 13.07)
60-64	11.99 (11.56, 12.43)	12.02 (11.48, 12.58)	12.04 (11.34, 12.78)	12.06 (11.15, 13.02)	12.09 (10.94, 13.32)	12.13 (10.71, 13.68)	12.2 (10.49, 14.09)	12.29 (10.27, 14.58)	12.41 (10.05, 15.13)	12.56 (9.85, 15.76)	12.73 (9.64, 16.46)	12.93 (9.44, 17.25)	13.16 (9.26, 18.14)	13.43 (9.08, 19.13)
65-69	17.31 (16.7, 17.94)	17.52 (16.74, 18.33)	17.72 (16.69, 18.8)	17.9 (16.54, 19.32)	18.03 (16.31, 19.87)	18.12 (16, 20.43)	18.18 (15.63, 21.01)	18.23 (15.23, 21.62)	18.28 (14.81, 22.29)	18.35 (14.39, 23.02)	18.44 (13.97, 23.85)	18.57 (13.56, 24.77)	18.74 (13.18, 25.82)	18.96 (12.81, 27)
70-74	26.67 (25.73, 27.64)	26.91 (25.71, 28.15)	27.16 (25.58, 28.81)	27.43 (25.36, 29.61)	27.74 (25.09, 30.56)	28.07 (24.79, 31.64)	28.44 (24.45, 32.85)	28.79 (24.05, 34.14)	29.11 (23.58, 35.49)	29.36 (23.02, 36.85)	29.55 (22.39, 38.22)	29.7 (21.69, 39.63)	29.83 (20.97, 41.1)	29.97 (20.25, 42.67)
75-79	41.82 (40.34, 43.33)	42.03 (40.16, 43.96)	42.24 (39.77, 44.8)	42.51 (39.29, 45.89)	42.84 (38.76, 47.2)	43.23 (38.17, 48.72)	43.66 (37.53, 50.43)	44.11 (36.85, 52.3)	44.59 (36.13, 54.36)	45.14 (35.39, 56.64)	45.76 (34.66, 59.17)	46.42 (33.91, 61.94)	47.08 (33.11, 64.87)	47.69 (32.22, 67.91)
80-84	65.99 (63.66, 68.38)	66.86 (63.88, 69.92)	67.75 (63.79, 71.85)	68.57 (63.38, 74.01)	69.23 (62.63, 76.27)	69.74 (61.57, 78.59)	70.15 (60.31, 81.03)	70.56 (58.94, 83.67)	71.09 (57.59, 86.66)	71.74 (56.25, 90.02)	72.49 (54.91, 93.74)	73.31 (53.55, 97.82)	74.2 (52.17, 102.24)	75.16 (50.79, 107.03)
85-89	130.02 (125.42, 134.72)	130.53 (124.7, 136.51)	131.25 (123.57, 139.2)	132.21 (122.2, 142.71)	133.47 (120.73, 147.04)	135.07 (119.25, 152.23)	136.96 (117.74, 158.2)	138.91 (116.04, 164.72)	140.75 (114.02, 171.57)	142.29 (111.57, 178.56)	143.53 (108.72, 185.62)	144.6 (105.63, 192.94)	145.7 (102.45, 200.76)	147.06 (99.37, 209.43)

90-94	243.08 (234.43, 251.91)	244.22 (233.28, 255.45)	245.15 (230.79, 260.03)	245.74 (227.12, 265.27)	246.21 (222.7, 271.26)	246.9 (217.97, 278.28)	248.06 (213.24, 286.55)	249.66 (208.54, 296.06)	251.76 (203.94, 306.91)	254.48 (199.53, 319.33)	257.9 (195.35, 333.53)	261.9 (191.31, 349.46)	266.1 (187.11, 366.66)	270.11 (182.52, 384.68)
95+	363.98 (350.84, 377.42)	364.03 (347.58, 380.95)	364.46 (343, 386.71)	365.33 (337.56, 394.47)	366.68 (331.6, 404.07)	368.46 (325.23, 415.35)	370.47 (318.43, 428.01)	372.23 (310.89, 441.46)	373.53 (302.55, 455.41)	374.72 (293.78, 470.26)	376.29 (285.01, 486.69)	378.65 (276.56, 505.27)	381.74 (268.4, 526.03)	385.67 (260.59, 549.27)

Table S15

Projections of high BMI-attributable CKD DALY burden for females with differences age groups from 2022-2035 globally.

	2022	2023	2024	2025	2026	2027	2028	2029	2030	2031	2032	2033	2034	2035
25-29	22.98 (21.99, 24.01)	23.79 (22.44, 25.2)	24.62 (22.79, 26.58)	25.49 (23.06, 28.14)	26.39 (23.25, 29.88)	27.34 (23.39, 31.81)	28.32 (23.47, 33.95)	29.36 (23.51, 36.31)	30.44 (23.49, 38.91)	31.58 (23.44, 41.77)	32.78 (23.34, 44.91)	34.04 (23.21, 48.38)	35.36 (23.04, 52.19)	36.77 (22.83, 56.39)
30-34	31.65 (30.54, 32.78)	32.66 (31.24, 34.12)	33.75 (31.86, 35.75)	34.92 (32.39, 37.63)	36.15 (32.83, 39.76)	37.43 (33.17, 42.15)	38.77 (33.42, 44.8)	40.16 (33.59, 47.73)	41.62 (33.69, 50.97)	43.15 (33.72, 54.54)	44.75 (33.68, 58.47)	46.43 (33.58, 62.8)	48.21 (33.42, 67.56)	50.07 (33.21, 72.81)
35-39	47.39 (45.76, 49.06)	48.2 (46.14, 50.34)	49.18 (46.44, 52.06)	50.36 (46.74, 54.23)	51.75 (47.06, 56.85)	53.32 (47.37, 59.89)	55.06 (47.69, 63.35)	56.95 (48, 67.24)	58.99 (48.27, 71.55)	61.13 (48.47, 76.3)	63.38 (48.58, 81.52)	65.74 (48.6, 87.26)	68.21 (48.54, 93.57)	70.82 (48.39, 100.51)
40-44	77.19 (74.56, 79.89)	78.57 (75.23, 82.03)	79.89 (75.47, 84.54)	81.15 (75.34, 87.36)	82.39 (74.93, 90.48)	83.69 (74.38, 93.99)	85.18 (73.8, 98)	86.98 (73.31, 102.67)	89.16 (72.97, 108.14)	91.74 (72.76, 114.46)	94.64 (72.61, 121.63)	97.87 (72.51, 129.69)	101.41 (72.43, 138.69)	105.22 (72.32, 148.63)
45-49	121.88 (117.74, 126.12)	125.1 (119.81, 130.6)	128.3 (121.22, 135.76)	131.36 (121.96, 141.41)	134.21 (122.08, 147.39)	136.87 (121.65, 153.7)	139.42 (120.8, 160.38)	141.88 (119.6, 167.47)	144.28 (118.08, 174.96)	146.65 (116.32, 182.96)	149.17 (114.45, 191.7)	152.05 (112.66, 201.49)	155.51 (111.07, 212.68)	159.7 (109.77, 225.6)
50-54	190.63 (184.18, 197.25)	195.08 (186.84, 203.63)	199.9 (188.88, 211.51)	204.97 (190.32, 220.63)	210.32 (191.33, 230.96)	215.99 (191.98, 242.52)	221.87 (192.25, 255.21)	227.74 (191.97, 268.79)	233.41 (191.04, 283.05)	238.75 (189.38, 297.87)	243.81 (187.08, 313.32)	248.72 (184.29, 329.56)	253.53 (181.08, 346.71)	258.26 (177.52, 364.81)
55-59	283.91 (274.31, 293.74)	286.92 (274.82, 299.48)	290.76 (274.74, 307.63)	295.48 (274.37, 318.04)	301.05 (273.87, 330.58)	307.54 (273.37, 345.32)	314.95 (272.92, 362.28)	323.02 (272.3, 381.24)	331.55 (271.37, 402.05)	340.61 (270.18, 424.93)	350.25 (268.75, 450.08)	360.32 (266.98, 477.42)	370.46 (264.6, 506.6)	380.35 (261.45, 537.27)
60-64	386.53 (373.46, 399.92)	390.49 (374.02, 407.58)	393.73 (372.04, 416.57)	396.44 (368.14, 426.71)	399.24 (363.21, 438.39)	402.73 (357.99, 452.19)	407.31 (352.95, 468.5)	413.12 (348.25, 487.56)	420.25 (343.97, 509.6)	428.68 (340.05, 534.8)	438.51 (336.49, 563.5)	449.74 (333.24, 595.91)	462.01 (330, 631.8)	475.05 (326.55, 671.02)
65-69	484.17 (467.82, 500.95)	492.41 (471.64, 513.96)	500.98 (473.38, 530.04)	509.22 (472.86, 548.1)	516.81 (470.16, 567.49)	523.53 (465.36, 587.82)	529.28 (458.64, 608.8)	534.14 (450.27, 630.39)	538.37 (440.66, 652.84)	542.81 (430.59, 677.18)	548.28 (420.72, 704.55)	555.33 (411.49, 735.81)	564.17 (402.98, 771.49)	574.93 (395.21, 812.1)
70-74	589.23 (569.33, 609.63)	595.37 (570.26, 621.42)	602 (568.85, 636.92)	609.26 (565.76, 655.76)	617.71 (561.96, 678.28)	627.58 (557.86, 704.65)	638.72 (553.48, 734.68)	650.41 (548.3, 767.61)	661.79 (541.68, 802.5)	672.44 (533.41, 838.9)	682.09 (523.4, 876.5)	690.6 (511.72, 915.04)	698.08 (498.63, 954.61)	704.87 (484.53, 995.62)
75-79	731.96 (707.23, 757.3)	738.81 (707.66, 771.14)	744.83 (703.81, 788.03)	751 (697.39, 808.33)	757.87 (689.47, 832.19)	765.54 (680.49, 859.55)	774.08 (670.77, 890.37)	783.39 (660.4, 924.55)	793.64 (649.6, 962.38)	805.6 (639.04, 1005.01)	819.57 (628.9, 1053.16)	835.35 (618.97, 1106.83)	852.02 (608.59, 1165.12)	868.47 (596.99, 1226.72)
80-84	835.36 (807.14, 864.3)	851.18 (815.28, 888.43)	867.13 (819.37, 917.43)	882.14 (819.14, 949.48)	895.31 (814.5, 983.12)	906.07 (805.4, 1017.35)	915.22 (793.07, 1052.72)	923.49 (778.5, 1089.9)	932.1 (762.93, 1130.27)	941.73 (747.02, 1174.84)	952.52 (730.92, 1224.01)	964.57 (714.72, 1278.05)	977.77 (698.4, 1337.07)	992.33 (682.12, 1401.66)
85-89	1233.38 (1191.69, 1276.12)	1248.26 (1195.6, 1302.91)	1264.58 (1194.92, 1337.95)	1282.67 (1191.08, 1380.6)	1303.32 (1185.68, 1431.15)	1327.1 (1179.65, 1490.09)	1353.22 (1172.61, 1556.54)	1379.79 (1163.15, 1628.44)	1405.11 (1150.08, 1703.88)	1427.78 (1132.58, 1781.23)	1446.86 (1110.24, 1859.25)	1463.62 (1084.5, 1939.31)	1479.26 (1056.6, 2022.86)	1495.7 (1028.14, 2112.69)

90-94	2026.25 (1957.69, 2096.53)	2055.17 (1968.38, 2145.22)	2080.43 (1965.74, 2201.2)	2101.93 (1951.79, 2262.45)	2123.64 (1931.91, 2331.95)	2148.26 (1909.55, 2412.13)	2175.76 (1885.35, 2502.69)	2206.15 (1859.74, 2603.72)	2240.01 (1833.43, 2716.31)	2278.76 (1807.6, 2842.87)	2323.41 (1782.84, 2985.65)	2372.65 (1758.05, 3143.78)	2423.18 (1730.81, 3313.68)	2472.03 (1699.25, 3491.8)
95+	3163.97 (3056.69, 3273.96)	3196.88 (3061.72, 3337.14)	3233.02 (3054.67, 3420.84)	3272.58 (3038.69, 3522.64)	3318.57 (3018.86, 3644.22)	3369.82 (2995.27, 3783.87)	3420.41 (2963.77, 3934.49)	3465.5 (2921.26, 4090.14)	3504.91 (2868.68, 4250.25)	3545.27 (2812.2, 4422.99)	3591.14 (2755.57, 4614.8)	3642.5 (2698.92, 4826.42)	3699.39 (2642.34, 5058.94)	3762.85 (2586.52, 5315.15)

Table S16

Projections of high BMI-attributable CKD DALY burden for males with differences age groups from 2022-2035 globally.

	2022	2023	2024	2025	2026	2027	2028	2029	2030	2031	2032	2033	2034	2035
25-29	30.62 (29.21, 32.08)	31.53 (29.69, 33.47)	32.48 (30.04, 35.08)	33.46 (30.29, 36.89)	34.47 (30.45, 38.92)	35.53 (30.54, 41.15)	36.62 (30.56, 43.61)	37.77 (30.52, 46.31)	38.96 (30.42, 49.26)	40.21 (30.27, 52.5)	41.51 (30.07, 56.03)	42.88 (29.83, 59.9)	44.32 (29.55, 64.12)	45.82 (29.23, 68.74)
30-34	38.25 (36.81, 39.72)	39.3 (37.56, 41.11)	40.44 (38.2, 42.79)	41.64 (38.74, 44.72)	42.89 (39.16, 46.91)	44.19 (39.46, 49.36)	45.53 (39.66, 52.08)	46.93 (39.77, 55.08)	48.38 (39.79, 58.38)	49.9 (39.73, 61.99)	51.48 (39.6, 65.95)	53.14 (39.4, 70.28)	54.87 (39.14, 75.02)	56.68 (38.82, 80.2)
35-39	57.76 (55.63, 59.95)	58.46 (55.89, 61.12)	59.34 (56.08, 62.75)	60.44 (56.27, 64.87)	61.82 (56.53, 67.51)	63.43 (56.82, 70.65)	65.22 (57.12, 74.23)	67.16 (57.4, 78.21)	69.2 (57.61, 82.57)	71.35 (57.74, 87.36)	73.58 (57.77, 92.59)	75.91 (57.7, 98.3)	78.35 (57.52, 104.54)	80.89 (57.26, 111.36)
40-44	88.85 (85.6, 92.19)	90.05 (86.12, 94.11)	91.09 (86.12, 96.29)	92.01 (85.7, 98.72)	92.91 (84.99, 101.45)	93.9 (84.14, 104.57)	95.1 (83.3, 108.21)	96.59 (82.56, 112.47)	98.48 (81.99, 117.49)	100.81 (81.61, 123.38)	103.54 (81.38, 130.15)	106.6 (81.21, 137.74)	109.91 (81.05, 146.12)	113.42 (80.83, 155.27)
45-49	128.83 (124.14, 133.65)	131.8 (126.08, 137.72)	134.77 (127.44, 142.45)	137.57 (128.14, 147.58)	140.1 (128.16, 152.96)	142.35 (127.57, 158.52)	144.35 (126.47, 164.24)	146.12 (124.92, 170.13)	147.73 (123.02, 176.24)	149.32 (120.9, 182.73)	151.07 (118.75, 189.87)	153.17 (116.7, 197.91)	155.79 (114.88, 207.12)	159.05 (113.34, 217.74)
50-54	206.36 (198.86, 214.05)	209.73 (200.64, 219.14)	213.51 (201.92, 225.66)	217.68 (202.77, 233.51)	222.28 (203.35, 242.66)	227.33 (203.74, 253.13)	232.71 (203.89, 264.75)	238.12 (203.57, 277.22)	243.27 (202.58, 290.19)	247.99 (200.81, 303.47)	252.24 (198.28, 317.01)	256.08 (195.12, 330.87)	259.57 (191.42, 345.07)	262.81 (187.29, 359.77)
55-59	301.29 (290.36, 312.51)	303.64 (290.5, 317.24)	306.79 (290.15, 324.23)	310.66 (289.39, 333.23)	314.99 (288.18, 343.86)	319.75 (286.59, 356.03)	325.17 (284.91, 369.94)	331.27 (283.21, 385.65)	338.02 (281.48, 403.2)	345.48 (279.76, 422.76)	353.72 (278.06, 444.53)	362.51 (276.22, 468.36)	371.43 (273.91, 493.76)	380.01 (270.83, 520.2)
60-64	411.34 (396.42, 426.65)	413.36 (395.47, 431.87)	414.66 (392.16, 438.21)	415.65 (387.2, 445.83)	417.1 (381.61, 455.32)	419.43 (375.94, 467.01)	422.95 (370.6, 481.17)	427.65 (365.62, 497.85)	433.4 (360.92, 516.96)	439.86 (356.19, 538.25)	446.99 (351.4, 561.75)	455.1 (346.78, 587.98)	464.25 (342.37, 617.14)	474.4 (338.09, 649.37)
65-69	499.29 (481.19, 517.88)	506.52 (484.6, 529.2)	513.69 (485.83, 542.88)	520.13 (484.53, 557.92)	525.46 (480.74, 573.62)	529.48 (474.57, 589.54)	532.39 (466.48, 605.68)	534.44 (456.92, 622.17)	536.17 (446.5, 639.54)	538.55 (436.12, 659)	542.15 (426.21, 681.33)	547.35 (417.08, 707.15)	554.16 (408.68, 736.65)	562.41 (400.82, 769.85)
70-74	630.67 (607.8, 654.14)	637.52 (609.94, 666.06)	644.38 (609.43, 680.99)	651.45 (606.87, 698.77)	659.37 (603.26, 719.79)	668.49 (599.17, 744.32)	678.57 (594.57, 771.98)	688.67 (588.79, 801.71)	697.89 (581.17, 832.44)	705.71 (571.48, 863.54)	711.87 (559.63, 894.63)	716.63 (546.07, 925.85)	720.34 (531.23, 957.55)	723.7 (515.78, 990.62)
75-79	794.15 (765.36, 823.71)	801.12 (766.47, 837)	807.97 (764.14, 853.87)	815.77 (759.95, 875.02)	824.33 (754.18, 899.86)	833.51 (747.08, 928.05)	843.06 (738.7, 959.12)	852.75 (729.06, 992.72)	862.82 (718.53, 1029.17)	874.14 (707.87, 1069.64)	887.18 (697.46, 1114.95)	901.63 (687.04, 1164.86)	916.26 (675.72, 1217.99)	929.85 (662.7, 1272.82)
80-84	977.47 (942.02, 1013.87)	992.21 (949.28, 1036.65)	1007.23 (952.58, 1064.46)	1021.35 (951.44, 1095.54)	1033.71 (945.74, 1128.45)	1044.32 (936.03, 1162.79)	1054.12 (923.63, 1199.23)	1063.88 (909.57, 1238.51)	1075.05 (895.26, 1282.32)	1087.36 (880.54, 1330.56)	1100.66 (865.28, 1383.23)	1114.6 (849.32, 1440.01)	1128.89 (832.52, 1500.64)	1143.86 (815.22, 1565.76)
85-89	1495.22 (1440.96, 1550.92)	1505.37 (1440.2, 1572.82)	1517.79 (1435.43, 1604.06)	1532.68 (1427.76, 1644.03)	1550.67 (1418.69, 1692.8)	1572.32 (1409.25, 1750.7)	1596.98 (1399.28, 1816.84)	1622.31 (1386.98, 1888.62)	1646.42 (1371.06, 1963.88)	1667.94 (1350.67, 2041.01)	1686.88 (1326.12, 2119.97)	1704.73 (1298.98, 2202.44)	1722.78 (1270.49, 2290.12)	1743.37 (1242.47, 2386.4)

90-94	2411.35 (2323.72, 2501.26)	2425.46 (2320.35, 2534.24)	2438.29 (2305.86, 2576.96)	2448.81 (2281.09, 2626.79)	2459.33 (2249.95, 2684.8)	2472.74 (2216.23, 2753.31)	2491 (2182.57, 2833.99)	2513.35 (2148.74, 2925.96)	2540.12 (2115.26, 3029.91)	2572.38 (2083.04, 3147.76)	2611.11 (2052.67, 3281.51)	2655.23 (2023.22, 3430.48)	2700.88 (1991.77, 3590.36)	2744.96 (1956.27, 3757.46)
95+	3354.87 (3232.57, 3480.42)	3361.84 (3215.83, 3513.01)	3371.48 (3188.12, 3563.54)	3384.02 (3152.05, 3630.24)	3400.25 (3110.59, 3712.19)	3419.76 (3064.87, 3807.98)	3441.83 (3015.55, 3915.91)	3462.51 (2960.09, 4031.09)	3480.34 (2898.13, 4151.56)	3498.62 (2833.01, 4281.3)	3521.47 (2768.27, 4425.72)	3551.72 (2706.27, 4588.82)	3588.3 (2646.15, 4770.12)	3631.71 (2588.19, 4971.37)
